# Users’ capabilities related to the electronic RHIS for newborn and stillbirth indicators: quantitative and qualitative findings of the IMPULSE study across 151 sites in the Central African Republic, Ethiopia, Tanzania, and Uganda

**DOI:** 10.7189/jogh.15.04239

**Published:** 2025-11-21

**Authors:** Ousman Mouhamadou, Lorenzo Giovanni Cora, Jacqueline Minja, Firehiwot Abathun, Rornald Muhumuza Kananura, Mary Ayele, Francesca Tognon, Giovanni Putoto, Johan Sæbø, Ilaria Mariani, Sara Geremia, Paolo Dalena, Donat Shamba, Marzia Lazzerini

**Affiliations:** 1 CUAMM Doctors with Africa, Bangui, Central African Republic; 2 Institute for Maternal and Child Health IRCCS Burlo Garofolo, Trieste, Italy; 3 Ifakara Health Institute, Ifakara, Tanzania; 4 CUAMM Doctors with Africa, Addis Ababa, Ethiopia; 5 Makerere University, Kampala, Uganda; 6 CUAMM Doctors with Africa, Padua, Italy; 7 University of Oslo, Oslo, Norway; 8 University of Trieste, Trieste, Italy; 9 London School of Hygiene & Tropical Medicine, London, United Kingdom

## Abstract

**Background:**

The electronic routine health information system (eRHIS) is crucial for policy and planning. However, its effectiveness depends on end-users’ capabilities in utilising it. Using a mixed-methods approach, we evaluated end-users’ data in the Central African Republic (CAR), Ethiopia, Tanzania, and Uganda in one of the first standardised cross-country assessment of eRHIS users’ capabilities focussed on newborn and stillbirth indicators.

**Methods:**

We collected data in 12 regions and 4 city administrations between November 2022 and July 2024 using the Every Newborn-Measurement Improvement for Newborn & Stillbirth Indicators (EN-MINI) Performance of Routine Information System Management (PRISM) tools. All data but staff opinions were collected through direct observation. We analysed quantitative questions and reported them as frequencies/normalised PRISM scores, both on the overall sample and by country. We analysed qualitative data using thematic analysis.

**Results:**

We included end-users of the eRHIS from 151 sites (56 data offices, 95 facilities). Their capabilities in utilising the eRHIS varied and were mainly higher in Uganda, followed by Tanzania, Ethiopia, and the CAR. End-users’ capabilities also varied by type of abilities, being in general higher for track report completeness (with Tanzania, Uganda, Ethiopia, and CAR having 6/10, 5/10, 4/10, and 2/10 indicators at >80%, respectively), compared to skills in data analysis and visualisation (with only Uganda showing 2/6 indicators >80% for both domains and the other countries having no indicators at >80%). Practical skills scores were low in all countries, particularly on plotting, problem-solving, and use of information. ‘Champion/good performer’ emerged in each country, with staff at higher health system levels showing the highest capabilities. End-users’ suggestions to improve the eRHIS (n = 127) were focussed on technical/software improvements (n = 73, 57.5%) and functionalities for data quality checks and data analysis (n = 36, 28.3%).

**Conclusions:**

Our findings suggest several common gaps in end-users’ capabilities in utilising the eRHIS, particularly in the CAR, and in all countries at facility levels.

Every year, an estimated 2.3 million newborns die during the first 28 days of life, with 1.9 million being stillborn; nearly all (98%) of these deaths occur in low- and middle-income countries (LMICs) [1,2]. The Every Newborn Action Plan set a target of 12 or fewer late gestation stillbirths per 1000 total births by 2030 [3]. According to the latest estimates, if current trends persist, 56 countries will not meet this target, with the countries the furthest away from the goal being those in sub-Saharan Africa and South Asia, where stillbirth rates are highest and data availability is the lowest [3–7].

Accelerating change requires improvements in the routine data collection of newborn and stillbirth data, including improving the use of existing electronic routine health information systems (eRHISs) [3]. Currently, more than 80 countries worldwide covering an estimated 3.2 billion people (40% of the world’s population) use the District Health Information System 2 (DHIS2) as their eRHIS platform [8]. The DHIS2 is offered free of charge as a global public good; however, as with every electronic data collection system, users need to know how to utilise its functionality to the fullest.

The critical role of end-users’ capabilities in utilising eRHISs has been underscored in systematic reviews that highlighted many underlying determinants contributing to their performance [9,10]. A recent multi-country review focussed on LMICs observed a need for combining interventions targeting behavioural, technical, organisational, and other factors to improve the performance of eRHISs, underling how the culture of data quality and use, and empowerment of the staff are key factors for an overall improvement [9]. A 2018 systematic review described how organisational and behavioural factors, information systems, capacity building, and economic issues play a critical role in data quality and use in LMICs, while pointing out a lack of literature describing system design barriers [10].

Despite the critical role of end-users’ capabilities in utilising the RHIS, we found few multi-country studies, particularly those conducted in African settings. Moreover, users’ capabilities – a different construct from more studied factors like software usability or infrastructure – have been rarely reported with an objective metric, with research most often relying on self-assessment. For example, a recent nationwide cross-sectional study in Tanzania reporting on over 2600 members of district health management teams found that only about half self-reported being trained on DHIS2 data analysis [11]. Another recent cross-sectional study among 378 health professionals in Ethiopia, also based on participants’ self-assessment, found that the level of good RHIS utilisation among health professionals was low, with lack of self-confidence and empowerment, complexity of RHIS formats, and poor organisational support significantly reducing RHIS utilisation [12].

IMProving qUaLity and uSE of newborn indicator (IMPULSE) is a collaborative project led by the London School of Hygiene and Tropical Medicine (LSHTM) in United Kingdom, Doctors with Africa *Collegio Universitario Aspiranti Medici Missionari* (*CUAMM*), the Ifakara Health Institute in Tanzania, and Makerere University in Uganda, and the World Health Organization (WHO) Collaborating Centre for Maternal and Child Health in Italy. Its goal is to improve the quality and use of neonatal indicators in the Central African Republic (CAR), Ethiopia, Tanzania, and Uganda.

Phase 1 of the IMPULSE project focussed on conducting a baseline assessment of newborn and stillbirth data quality and use, and related input and process factors, as per the Performance of Routine Information System Management (PRISM) Framework [13] (Figure S1 in the **Online Supplementary Document**). This evaluation – the first of its kind in the four countries included in phase 1 of the project – will contribute as a baseline assessment critical for identifying priorities for interventions to be developed in Phase 2.

A part of a research theme in this Journal, this work specifically focusses on capabilities of users from the four countries to utilise the functions of the eRHIS, the DHIS2, and Excel (only for facilities in the CAR) for newborn and stillbirth indicators.

## METHODS

### Study design and participants

This was a cross-sectional study based on quantitative and qualitative methodological approaches. We followed the STROBE guidelines for cross-sectional studies [14] and the SRQR guidelines for qualitative research [15] when reporting our findings (Tables S1 and S2 in the **Online Supplementary Document**).

We conducted this research in 12 regions and 4 city administrations in the CAR, Ethiopia, Tanzania, and Uganda (Tables S3 and S4 in the **Online Supplementary Document**). To select regions, we used a balance of the following three criteria: heterogeneity, *i.e.* we selected regions with different characteristics, including underperforming for maternal and neonatal mortality and/or hard to reach areas/humanitarian settings; travel – we preferred regions where the implementing agency, Doctors with Africa *CUAMM*, had an office/project that could facilitate co-ordination, or regions that were easy to reach; we chose regions requested for prioritisation by the local ministry of health.

The inclusion criteria (**Table 1**; Table S5 in the **Online Supplementary Document**) focussed on comprehensive emergency obstetric and newborn care health facilities with newborn essential care with or without neonatal inpatient care. We strived to include different levels and types of facilities, including all data offices receiving data from the selected facilities. We also prioritised higher-level facilities with higher numbers of deliveries. For practicality, we sampled health centres within the same district as the larger facilities. One national hospital or the largest facility was assessed in each studied country.

**Table 1:** Sample size by major characteristics, n (%)

	CAR	Ethiopia		Tanzania		Uganda	
**Facility type**							
Central/regional health data office	1 (4.7)	5 (14.3)		4 (8.7)		0 (0)	
District/subnational health data office	6 (28.6)	6 (17.1)		14 (30.4)		20 (40.8)	
First level of referral health facility	7 (33.3)	11 (31.4)		9 (19.6)		12 (24.5)	
Second level of referral hospital	3 (14.3)	10 (28.6)		14 (30.4)		12 (24.5)	
Third level of referral hospital	4 (19)	3 (8.6)		5 (10.9)		5 (10.2)	
**Setting**							
Rural	1 (7.1)	6 (25)		20 (71.4)		16 (55.2)	
Urban	13 (92.9)	18 (75)		8 (28.6)		13 (44.8)	
**Managing authority**							
Public	12 (85.8)	18 (75)		20 (71.4)		22 (75.9)	
NGO/not-for-profit	0 (0)	0 (0)		1 (3.6)		5 (17.2)	
Private-for-profit	1 (7.1)	4 (16.7)		2 (7.1)		0 (0)	
Mission/faith-based/CBO	1 (7.1)	2 (8.3)		4 (14.3)		2 (6.9)	
Other	0 (0)	0 (0)		1 (3.6)		0 (0)	
**Regions**							
Bangui City Administration	4 (19.0)						
Health region 1	4 (19.0)						
Health region 2	6 (28.6)						
Health region 7	7 (33.3)						
Addis Ababa City Administration		5 (14.3)					
Amhara and Gambella		5 (14.3)					
Oromia		12 (34.3)					
South Ethiopia and Sidama		13 (37.1)					
Dar es Salaam City Administration				3 (6.5)			
Iringa				16 (34.8)			
Shinyanga				13 (28.3)			
Simiyu				14 (30.4)			
Kampala City Administration						1 (2.0)	
Karamoja						16 (32.7)	
Lango						15 (30.6)	
West Nile						17 (34.7)	

CBO – community-based organisation, NGO – nongovernmental organisation

We excluded sites in areas with ongoing conflicts or where the road conditions were not safe enough to allow for the assessment to be performed (five health facilities and one subnational office in Ethiopia and one health facility in Uganda). Due to internal security constraints in the CAR, we focussed the assessment on Bangui city and Health Regions 1, 2, and 7 only, and we included seven basic emergency obstetric and neonatal care healthcare facilities to increase the sample (Tables S6 and S7 in the **Online Supplementary Document**).

### Data collection tools

Data collection took place between November 2022 and July 2024 using the open-access Every Newborn-Measurement Improvement for Newborn & Stillbirth Indicators (EN-MINI) PRISM tools [16], a tested adaptation of the standard PRISM tools in use for over twenty years that includes ready-to-use digital data collection tools [17]. The IMPULSE team contributed to making the EN-MINI tool, version 2, available as a global good, and testing it in plot.

Here we utilised the eRHIS Usability EN-MINI-PRISM tool 3.2, which is specific to sites where an eRHIS is in use, as well as select questions from the Organizational and Behavioural Assessment EN-MINI-PRISM tool 6, which, *via* paper-based assessments that favour the cross of information from different sections of the survey, focusses on testing capacities critical to the use of an eRHIS [18]. Although not directly proposed in the PRISM manual as a data analysis plan [18], we decided on this approach to more deeply explore users’ capabilities (**Table 2**).

**Table 2:** Type of indicators utilised and related description by tool

	Number of indicators	Description of the indicators		
**Tool 3.2**				
Reporting capabilities				
*Track report completeness using eRHIS*	2	Software produces a report on the number and percentage of reports received out of the total number of expected reports.		
*Capacity to generates summary reports*	8	Software generates summary reports for the aggregate levels and time periods.		
Calculation capabilities				
*Ability to calculate coverage indicators*	30	User can calculate coverage for three predefined newborn indicators.		
Data analysis capabilities				
*Major causes neonatal mortality*	2	User can generate major causes of institution-based (inpatient, emergency) mortality.		
*Major causes neonatal morbidity*	2	User can generate major morbidity diagnoses for inpatient and outpatient services.		
*Data disaggregation*	2	User can show age and sex disaggregated data for the selected indicators.		
*Visualisation capabilities*	6	Percentage of staff able to use the data visualisation features of the eRHIS to analyse and present data in graphs and maps.		
**Tool 6**				
Practical skills				
*Calculating indicators*	8	User can calculate newborn indicators related to a simulated scenario by hand.		
*Plots/charts*	3	User can produce by hand plots and charts related to simulated newborn data.		
*Problem solving*	8	User can interpret simulated graphs and extract meaningful information for guidance.		
*Use of information*	13	User can describe simulated scenarios, pointing out problematic aspects and findings.		

eRHIS – electronic routine health information system

### Data quality assurance

Quality assurance procedures have been extensively reported elsewhere [19]. Data was collected by teams of three to nine trained operators guided by an experienced study coordinator (FA, JM, MA, MKR, or OM). Data were directly entered into an open data kit-based secure-digital platform SurveyCTO, version 2.71 (Dobility, Inc., Washington, DC, USA), which included checks for data completeness and plausibility. We used a shared regular monitoring and evaluation Excel file reviewed by a supervisor (FT) to keep track of data timeliness, completeness, and sample size.

We assigned scores according to rules pre-defined within the PRISM manual, allowing for objective and unbiased data collection, thus preventing bias due to the subjectivity of self-assessment. Independent data analysts (IM, PD, SG) ran four rounds of interim analyses within the first month of data collection. Any discrepancy in the data was discussed during weekly meetings or *via* real-time communication. Additionally, in the data analysis phase, results of analyses were cross-checked against results of the EN-MINI-PRISM automated analysis tool [18]. We also held a data validation workshop in each country to discuss the findings of the IMPULSE study phase 1 and their implications.

### Data analysis

We followed a pre-defined plan of analysis developed according to the indications of the PRISM User’s Kit 2019 [18]. We conducted analyses both on the overall sample and stratified by country, facility type (facilities with first-level referral *vs*. second-level referral *vs*. third-level referral; district data offices *vs*. regional data offices; region *vs*. region). Specific to the lattermost analyses, we looked at city administrations separately from the rest of the country’s regions. We could not perform multi-way stratification analysis due to the reduced sample size in each category. These analyses were carried out in R, version 4.3.1 (R Foundation for Statistical Computing, Vienna, Austria). 

To determine practical suggestions for eRHIS improvement, we explored the following research question through the qualitative thematic analysis of the survey’s open-ended questions: ‘Describe any improvement you would like to see in the eRHIS’. We tabulated all responses, and when one end-user reported more than one comment, we separated different comments. We calculated the relative frequencies of suggestions in each theme using the overall number of extracted comments (n = 127) as the denominator. We classified answers without any specific suggestion as ‘missing suggestion’. Three researchers (LGC, OM, ML) identified major themes, main themes, and sub-themes through a three-step inductive process: sub-themes were first defined by identifying common keywords in the comments (*e.g.* ‘software improvements’, ‘training staff’), after which main themes and sub-themes were created to allow for an accordingly detailed categorisation. When keywords could not be identified due to the format of the answer, we deduced the improvement desired by the respondent. Examples of key comments from end-users were also extracted and selected in agreement among authors. The three analysts resolved any disagreements through discussion.

### Ethical aspects

The data collection process aligned with General Data Protection Regulation (GDPR) regulations. Any information that could disclose participants’ identity was not collected in order to ensure anonymity. In detail, we collected data through password-protected tablets or phones using the EN-MINI-PRISM, version 2 open data kit forms, which was then uploaded to a secure encrypted server hosted by SurveyCTO through a community license. We collected the region and site name and the participants’ role, but did not gather any individual identifiers. Data were downloaded in-country, checked to ensure they did not include any key informant identifiers, and securely transferred to the study group. Paper documents were stored in locked filing cabinets. Most data were collected by direct observation or by registering anonymous respondents’ opinions and scores obtained by participants in pre-defined tests. Before starting data collection, the staff were presented with the objectives and methods of the study, queried for their consent to participate in the survey, and explained their rights to decline participation in an information sheet, after which they were asked to provide written consent before responding to the survey.

## RESULTS

### Sample characteristics

The sample included 151 sites (56 data offices; 95 facilities): 21 from the CAR, 35 from Ethiopia, 46 from Tanzania, and 49 from Uganda. Most of the health data offices district/subnational (n = 46, 82.1%). There were 17 (17.9%) third-level, 39 (41.1%) second-level, and 39 (41.1%) first-level referral facilities.

### Capabilities observed

**Figure 1:**
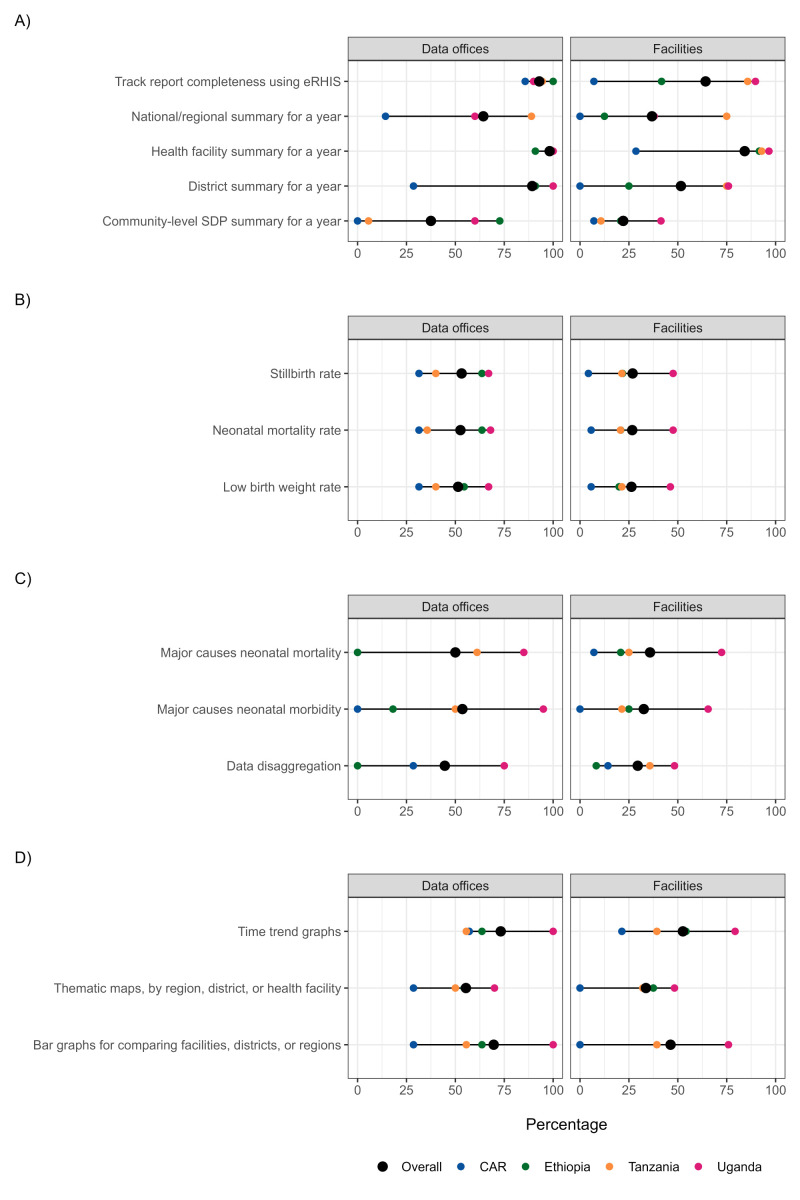
Capabilities of the eRHIS users (n = 151 sites). **Panel A.** Generating reports. **Panel B.** Calculating coverage. **Panel C.** Data analysis. **Panel D.** Data visualisation. Each dot in the figure represents the value for one country. When two values overlap, one dot may not be visible. CAR – Central African Republic, SDP – service delivery point.

The users’ capabilities we observed here varied in frequencies both by country and across the four key domains explored – generating reports, calculating coverage, data analysis, and data visualisation. However, some pattern of distribution could be observed (**Figure 1**). Data analysis capabilities were focalised in the users’ abilities in generating and showing specific key indicators (*e.g.* preterm birth, birth asphyxia, sepsis, low birthweight, retinopathy of prematurity, *etc.*).

When data were examined by country (Table S8–11 in the **Online Supplementary Document**), users from the CAR (**Figure 1**) showed the lowest frequencies across all four domains, while those from Uganda tended to show the highest. The domain on capabilities in generating reports had the highest frequencies (**Figure 1**, Panel A), with all countries showing high rates on tracking report completeness at data offices level (ranging between 85.7% in the CAR and 100% in Ethiopia). The capabilities domains pertinent to calculating coverage (**Figure 1**, Panel B) and to data analyses (**Figure 1**, Panel C) showed the lowest overall frequencies, with all countries showing frequencies on all indicators below 75%, expect Uganda on the indicators regarding data analysis at data offices level (ranging between 75% and 95%). Users’ capabilities were overall significantly less frequent at facility level compared to data office level, in particular in the domains of calculating coverage (**Figure 1**, panel B). The pattern of distribution of staff performance in each country was somewhat similar between sites level, suggesting a sensible structural difference in the users’ capabilities between countries, with the CAR showing major gaps. Results highlighted low users’ capabilities for newborn data specific tasks (**Figure 1**, Panel B).

### Practical skills test

**Figure 2:**
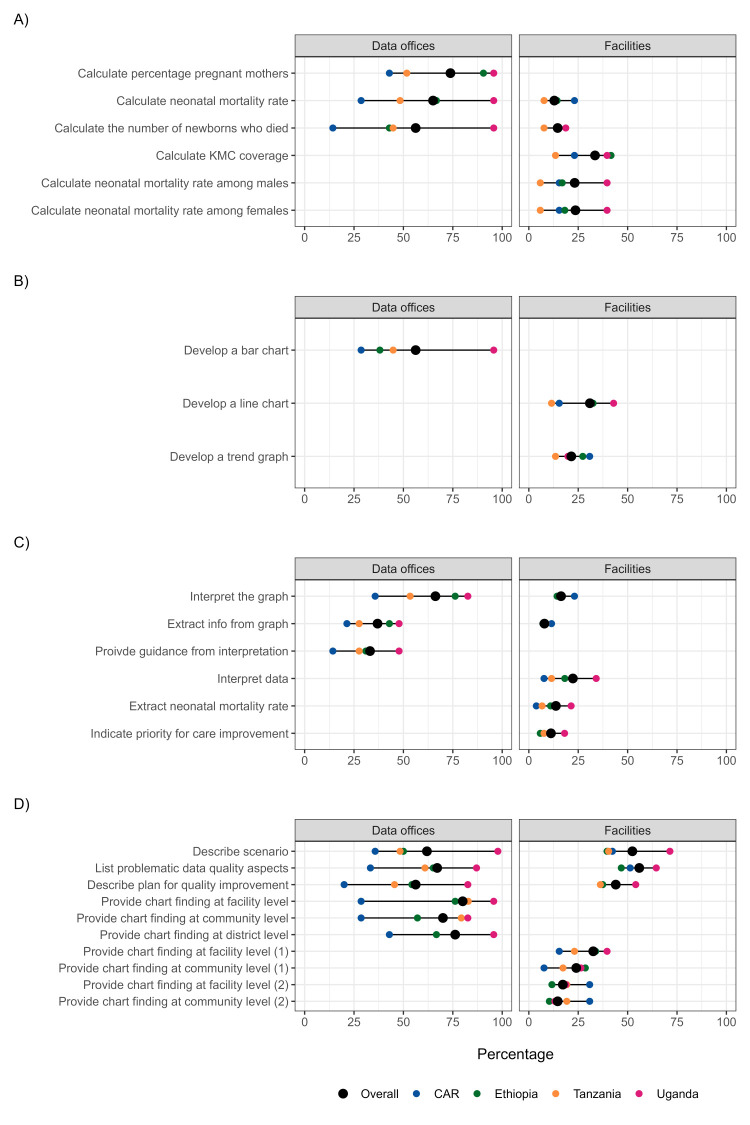
Practical skills (n = 313 respondents). **Panel A.** Calculating indicators. **Panel B.** Plots/charts. **Panel C.** Problem solving. **Panel D.** Use of information. ‘Provide chart finding at facility level’ and ‘provide chart finding at community level’ are divided based on the pre-defined scenario the set of questions they refer to. An empty space indicates that the specific indicator was not collected at that specific site level. Each dot in the figure represents the value for one country. When two values overlap, one dot may not be visible. CAR – Central African Republic, KMC – kangaroo mother care.

The practical skills test, assessed in 313 respondents (**Figure 2**) – 20 from the CAR, 98 from Ethiopia, 81 from Tanzania and 114 from Uganda – showed a similar pattern as observed for capabilities (**Figure 1**). When data were looked at by country, users from the CAR showed the lowest frequencies, while those from Uganda tended to show the highest frequencies, across all four domains. Practical skills were significantly less available at facility level compared to data office level, with all indicators measured at facility level, in any of the four domains explored, showing an overall frequency below 50% for the vast majority, and all countries consistently showing low frequencies, except few indicators describing the use of information (**Figure 2**, Panel D). At data office level, overall frequencies were almost always below 80%; however, all practical skills were available in over 80% of users in Uganda aside two indicators for problem solving. Similarly to the pattern of distribution observed for users’ capabilities, also the practical skill tests showed low staff performance at the facility level for the calculation of indicators and the production of plots, and a lack of skills in providing findings from charts and data interpretation. The presence of a “champion”, being Uganda, suggests, once again, a noticeable difference in structure between countries linked to the skills of the eRHIS users. Detailed data are available in Table S12 in the **Online Supplementary Document**.

We designed a list of area of overall good staff performance in each country vs. priority area for intervention to identify cross-country strength and weaknesses (**Table 3**). Results were selected by identifying the highest and lowest reported indicators in terms of percentages across the assessed areas of competence. The problem-solving practical skills at facility level was a priority area for intervention in all countries, with reported percentages never reaching above 50%. Conversely, generating health facility summary reports was a skill positively reported across all four countries and at any site level, with performances never being below 90% (aside for CAR at facility level).

**Table 3:** Good staff performance area and priority area for intervention, by country

Country	Facility type	Area of competence	Indicator (%)
**Practical skills observed**			
Good staff performance, by country			
*CAR*	Data offices	Generating reports	Health facility summary for a year (100)
*CAR*	Facilities	Generating reports	Generating health facility summary reports for a year (28.6)
*Ethiopia*	Data offices	Generating reports	Track report completeness using eRHIS (100)
*Ethiopia*	Facilities	Generating reports	Generating health facility summary reports for a year (91.7)
*Tanzania*	Data offices	Generating reports	Generating health facility summary reports for a year (100)
*Tanzania*	Facilities	Generating reports	Generating health facility summary reports for a year (92.9)
*Uganda*	Data offices	Generating reports	Generating health facility summary reports for a year (100)
*Uganda*	Data offices	Generating reports	Generating district summary reports for a year (100)
*Uganda*	Data offices	Generating reports	Use of time trend graphs (100)
*Uganda*	Data offices	Generating reports	Use of bar graphs (100)
*Uganda*	Facilities	Generating reports	Generating health facility summary reports for a year (100)
Priority areas for intervention, by country			
*CAR*	Data offices	Generating reports	Generating community-level summary reports for a year (0)
*CAR*	Data offices	Data analysis	Use of data analysis for major causes of neonatal mortality (0)
*CAR*	Data offices	Data analysis	Use of data analysis for major causes of neonatal morbidity (0)
*CAR*	Facilities	Generating reports	Generating national/regional summary reports for a year (0)
*CAR*	Facilities	Generating reports	Generating district summary reports for a year (0)
*CAR*	Facilities	Data analysis	Use of data analysis for major causes of neonatal morbidity (0)
*CAR*	Facilities	Data visualisation	Use of thematic maps (0)
*CAR*	Facilities	Data visualisation	Use of bar graphs (0)
*Ethiopia*	Data offices	Data analysis	Use of data analysis for major causes of neonatal mortality (0)
*Ethiopia*	Data offices	Data analysis	Use of data analysis for data disaggregation (0)
*Ethiopia*	Facilities	Data analysis	Use of data analysis for data disaggregation (8.3)
*Tanzania*	Data offices	Generating reports	Generating community-level summary reports for a year (5.6)
*Tanzania*	Facilities	Generating reports	Generating community-level summary reports for a year (10.7)
*Uganda*	Data offices	Generating reports	Generating community-level summary reports for a year (60.0)
*Uganda*	Facilities	Generating reports	Generating community-level summary reports for a year (41.4)
**Practical skills**			
Good staff performance, by country			
*CAR*	Data offices	Calculating indicators	Calculate percentage pregnant mothers (42.9)
*CAR*	Data offices	Use of information	Provide chart finding at district level (42.9)
*CAR*	Facilities	Use of information	List problematic data quality aspects (51.3)
*Ethiopia*	Data offices	Calculating indicators	Calculate percentage pregnant mothers (90.5)
*Ethiopia*	Facilities	Use of information	List problematic data quality aspects (46.8)
*Tanzania*	Data offices	Problem solving	Provide chart finding at facility level (82.8)
	Facilities	Use of information	List problematic data quality aspects (55.8)
*Uganda*	Data offices	Use of information	Describe scenario (97.8)
	Facilities	Use of information	Describe scenario (71.4)
Priority area for intervention, by country			
*CAR*	Data offices	Calculating indicators	Calculate the number of newborns who died (14.3)
*CAR*	Data offices	Problem solving	Provide guidance from interpretation (14.3)
*CAR*	Facilities	Problem solving	Extract neonatal mortality rate (3.8)
*Ethiopia*	Data offices	Problem solving	Provide guidance from interpretation (14.3)
*Ethiopia*	Facilities	Problem solving	Indicate priority for care improvement (5.8)
*Tanzania*	Data offices	Problem solving	Extract info from graph (27.6)
*Tanzania*	Data offices	Problem solving	Provide guidance from interpretation (27.6)
*Tanzania*	Facilities	Calculating indicators	Calculate neonatal mortality rate among males (5.8)
*Tanzania*	Facilities	Calculating indicators	Calculate neonatal mortality rate among females (5.8)
*Uganda*	Data offices	Problem solving	Extract info from graph (47.8)
*Uganda*	Data offices	Problem solving	Provide guidance from interpretation (47.8)
*Uganda*	Facilities	Problem solving	Extract info from graph (7.7)

CAR – Central African Republic, eRHIS – electronic routine health information system

### Users’ perspective

**Figure 3:**
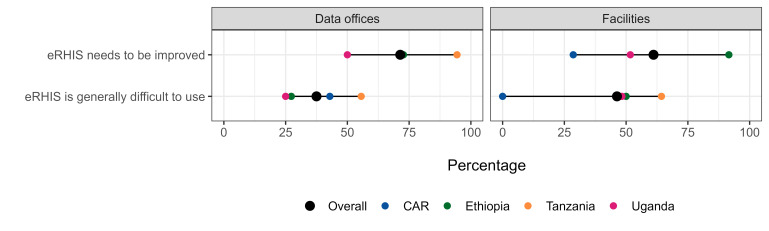
Users’ perspective regarding the eRHIS (n = 151 respondents) In the image each dot represents the value for one country. When two values overlap, one dot may not be visible. CAR – Central African Republic, eRHIS – electronic routine health information system.

Users’ perspective varied by country and by health system level (**Figure 3**). Overall, about two thirds of users either at data office level (71.4%) or at facility level (61.1%) stated that the eRHIS needs to be improved, with the higher frequencies being reported in Ethiopia at facility level (91.6%) and in Tanzania at data office level (94.4%).

Overall, about one third of users at data office (37.5%) and half at facility level (46.3%) stated that the eRHIS is generally difficult to use, with the higher frequencies being reported again in Tanzania, both at facility level (64.3%) and at data office level (55.6%) (Table S13 in the **Online Supplementary Document**). Results at data office level were in line with the users’ capabilities and practical skill tests results: better performing countries rate the eRHIS as generally not hard to use yet demand some improvements. At facility level, users in the CAR – the worst performing overall in terms of users’ capabilities – tended to undervalue the difficulty in utilising the eRHIS and did not express a strong need for improvements.

### Subgroup analysis by site level

When data were analysed by site level and when they were disaggregated and compared across different levels (facilities at first level of referral *vs*. second level of referral *vs*. third level of referral; district data offices *vs*. regional data offices), a large within country variability was observed (Figures S2.1–3.4 and Tables S14–19 in th**e ****Online Supplementary Document**).

In all countries, for most indicators staff in sites of higher level (*i.e.* staff working in 3rd level facilities and in regional health data office compared to the lower levels - identified with either a triangle or a rhombus - had higher capabilities in the use of the eRHIS. Results on practical skills tests on the other hand did not show a clear pattern of distribution across sites.

These subgroup analyses also highlighted the existence of ‘champion/good performer’ in each county, at least on some specific capabilities. This included staff at regional health offices showing high capabilities in the CAR, Ethiopia, Tanzania, and Uganda, with staff at district health data offices in Uganda presenting high capabilities.

The users’ perspective regarding the eRHIS improvements and difficulty of use did not present a clear structure by site level (Figures S4.1–4.4 in the **Online Supplementary Document**). In general, users at higher-level sites tend to rate the eRHIS easier to use, yet still declared a higher need for improvements.

### Subgroup analysis by region

We again observed strong within-country variability with some ‘champions/good performers’ in terms of the users’ capabilities of utilising the eRHIS. Specifically, the sites assessed in Bangui City Administration, Addis Ababa City Administration, Dar es Salaam City Administration, and the West Nile Region were ‘champions/good performers’ within the context of their corresponding country (Tables S20–24 in the **Online Supplementary Document**).

### End-users’ suggestions for improving the eRHIS

Out of 127 comments extracted as responses to the open text question ‘Describe any improvement you would like to see in the eRHIS’ (Tables S25 and S26 in the **Online Supplementary Document**), the major theme emerged was improved technical aspects (n = 109, 85.2%), with two subthemes: technical/software improvement (n = 73, 57.5%) and improved functionalities for data quality checks/data analysis (n = 36, 28.3%). An additional key theme emerged related to staff capacity strengthening (n = 14, 11%). Examples of significant suggestions include:

− ‘Data visualisation should be user friendly’;

− ‘All indicators should be defined clearly in system’;

− ‘Timely mentorship on system updates in case of any’;

− ‘Trainings should be done and supply of computers in lower facilities’.

## DISCUSSION

This study adds to existing literature by providing new evidence on service users’ capabilities in utilising the eRHIS (namely, the DHIS2) in four countries in Africa. It highlights several common gaps in users’ capabilities, although with substantial variation among countries, with staff in Uganda nearly always showing the highest level of capabilities, staff in Ethiopia and Tanzania showing variable levels of capabilities, and staff in the CAR showing in general low capabilities. Variations were also observed by type of capabilities and among different levels of the health system, with data office staff usually performing better than facility staff and regional data offices performing better than lower level ones. Common major gaps included capabilities on calculating coverage for newborns/stillbirth indicators and practical skills on plotting, problem solving, and use of information, particularly for staff at facility level. These findings align with previous studies on the DHIS2 in other countries [20,21]. The existence of ‘champions/good performers’ in each county, at least on some specific capabilities, suggests that good capabilities are achievable, in principle [22].

The common finding across four countries of end-users lacking capabilities on utilising the eRHIS may be considered a system design factor hampering data quality or data use connected to many other aspects of the health system. A recent systematic review of 316 studies identified a paucity of research describing system design factors hampering data quality and/or data use for decision making [20]. Another review published by the MEASURE Evaluation [23] described multiple facilitators that strongly influence the ability of individuals and organisations to use data effectively, such as system design, lack of data use culture, low individual commitment and motivation, and lack of organisational structure. Similarly, the WHO Quality of care Network [24] – a large project established in 11 African countries specifically aiming at improving the quality of maternal and newborn care – recognised four major systems required to sustain implementation of quality of care: on-site support, data systems, learning systems, and programme management for quality of care. Several other studies [25,26] suggested the importance of local ownership and accountability for designing strategies for improving local capacities, resources, and data use.

Some of our findings, such as the low capacity to produce thematic maps, may be explained by context factors such as existing DHIS2 functionalities and local set-up. For example, in all countries included in the study, the facilities configured in DHIS2 did not yet have geographical coordinates allowing them to produce maps. More specific to CAR, the DHIS2 has recently been installed at District Offices, so it was available but not yet fully operational at the time data were collected.

Other system factors that can affect staff capabilities in using the eRHIS can include availability of human and physical resources and high staff turn-over, governance and leadership factors (*e.g.* on training plans), existence of effective feedback system and supervision, and behavioural factors. While indicator we could not conduct an analysis to see how different domains (*e.g.* managerial vs competences) associated with each other due to the absence of a composite synthetic, we noticed a pattern of distribution of findings across countries, with Uganda, Ethiopia, and Tanzania always performing better than the CAR.

Overall, our findings strongly call for action to increase health workforces’ capabilities in utilising the eRHIS for newborn and stillbirth data, and the related health system factors directly affecting it. Notably, workforces’ retention and capacity development are crucial aspects for effective health system, as emphasised by the WHO Global Strategy on Human Resources for Health: Workforce 2030 [27]. The WHO estimates a projected shortfall of 10 million health workers by 2030, mostly in LMICs [28]. In response to this crisis, the World Health Assembly called member states ‘to implement the Working for Health 2022–2030 Action Plan and integrate, as appropriate, its objectives and actions for workforce planning and financing, education and employment, and protection and performance within their health and care workforce strategies, investment plans and programmes at national and subnational levels in line with previous resolutions’ [29]. In relation to training, it called member states ‘to engage at the national, regional and global levels to undertake and accelerate work on building a health and care workforce through training programmes and using best available educational and training facilities, online platforms and hybrid learning opportunities; and to increase the absorption of trained staff into health and care systems through sustainable employment practices’ [29]. Online training holds great promise for overcoming some of the associated resource constraints, with experiences emerging from similar settings which could be built upon [30].

These findings should be used by ministries of health, as well as by international technical and implementing agencies such as the WHO, UNICEF, and others, in designing workforce and digital strategy interventions, as presented in a review published in 2018 [31] and a study conducted in 2019 in the Eastern Mediterranean Region [32]. At national levels, they should be translated into concrete next steps (*e.g.* digital training programs, supervisory reforms) tailored to the observed needs of each setting. Increasing capability of end users should plausibly improve eRHIS data use for quality improvement purposes, thus contributing to improving newborn health outcomes. At international level, in times of digital transition, end-user capabilities need to be carefully considered when thinking about introducing new systems, since clearly inadequate user capacity, together with other factors such as heavy workload, can undermine the promise of digital transformation of health systems.

Many underlying factors can affect end-users’ capabilities on the eRHIS, including, well designed training and supervision, capacity monitoring, and a supportive environment. Overall, the users’ capabilities and level of training expressed in the practical skills were, in fact, higher for higher level sites, indicating that strong organisationally functioning structures allow the users to fully utilise their skills. This is noticeable upon analysing the users’ suggestions, where most respondents expressed the need to have more user-friendly software which can be further adapted to the needs of the site (*e.g.* working offline or producing maps with geographical data), rather than a need for improvement in physical and human resources. More implementation studies should help us understand how to deliver interventions (*e.g.* decentralised mentorship models or modular online training) and other support systems to improve effectiveness in terms of good competences in a sustainable format. The detailed findings discussed here are a first step towards the design of tailored setting-specific materials.

Notably, the need for capacity strengthening on the use of eRHISs is not unique to LMICs. A recent nationwide survey in Finland across over 3000 nurses identified three profiles labelled as low, moderate, and high competence groups, with a younger age, recent graduation year, and sufficient orientation being associated with nurses belonging to a high or moderate competence group [33]. Since higher competence has been shown to significantly associate with higher perceived eRHIS usefulness and staff motivation for users to continue to use the technology [34], increasing users’ capabilities could contribute to higher usefulness of the eRHIS in terms of supporting the nurses' work tasks and promoting the quality of care.

This study also highlights a lack of predefined methodologies to analyse in depth users’ capabilities on the eRHIS and underlying causes. The PRISM formulas [18], although useful in exploring many indicators, lack linkage between indicators, favouring a general overview of the studied setting without deeply exploring causality between analysed factors, and do not accomodate a composite summary indicator, thus hampering multivariate analyses. Future research should aim to identify comprehensive and meaningful indicators that can summarise the performance of a health worker, as well as other major domains reflecting key characteristics of the setting, such as physical resources, and training, to allow for a comprehensive and synthetic description.

The EN-MINI-PRISM tool 3.2 did not collect sociodemographic information regarding end-users; however, the standard operating procedures for data collection indicated to include key end users, as we reported above. We utilised PRISM standardised tools and data collection procedures, allowing us to compare data over time and between settings. We also used objective and comparable metrics to test the actual skills of the end users, rather than self-assessed perceptions that might have exposed our results to social desirability and subjective perceptions [35]. Most importantly, this study provides data for action, which can be utilised to inform policies at national and global level to improve the effectiveness of the eRHIS to the benefit of both newborns and staff.

We acknowledge that the results of this IMPULSE study cannot be generalised to different study settings, but note that the standardised data collection and analysis procedures allow for replicability in other settings. Although the limited sample size in the CAR and in Ethiopia due to security reasons, the data collected in over 150 sites allows for a broad overview of the users’ abilities related to the eRHIS, specific to newborn and stillbirth indicators, and according to a predefined PRISM framework [18] and suggest the existence of some common actionable gaps.

## CONCLUSIONS

Given the many gaps presented in terms of users’ capabilities towards the eRHIS across the four countries, improvements are necessary to guarantee that the functionalities of the eRHIS are used at its best. Setting-specific interventions are required to improve users’ capabilities in both data analysis (*e.g.* plotting charts, calculating newborn and stillbirth indicators, calculating coverage indicators) and data reporting (*e.g.* generating report, data visualisation). Particular attention should be given to the actual use of data, as this a key aspect for improving quality of care. Without trustworthy and accurate data, improvements on maternal and newborn health are heavily hindered. Within the context of the considered African settings, the DHIS2 is already a well-developed and established system, but lacks ‘polishing’ in the very last steps of the data management process, leading to poor or even non-existent use of data. Targeted investments would open up the possibility of meaningful improvements that could also be adapted to different settings for better maternal and newborn quality of care globally.

## Additional material


Online Supplementary Document

## References

[1] Chan GJ Goddard FGB Hunegnaw BM Mohammed Y Hunegnaw M Haneuse S Estimates of Stillbirths, Neonatal Mortality, and Medically Vulnerable Live Births in Amhara, Ethiopia. JAMA Netw Open. 2022;5:e2218534. 10.1001/jamanetworkopen.2022.18534 35749113 PMC9233235

[2] United Nations Inter-Agency Group for Child Mortality Estimation. Levels and trends in child mortality. New York, USA: United Nations Children's Fund; 2024. Available: https://data.unicef.org/resources/levels-and-trends-in-child-mortality-2024/. Accessed: 12 August 2024.

[3] World Health Organization. Every Newborn: an action plan to end preventable deaths. Geneva, Switzerland: World Health Organization; 2014. Available: https://www.who.int/initiatives/every-newborn-action-plan. Accessed: 12 August 2024.

[4] World Health Organization. Newborn mortality. 14 March 2024. Available: https://www.who.int/news-room/fact-sheets/detail/newborn-mortality. Accessed: 12 August 2024.

[5] United Nations. Transforming our world: the 2030 Agenda for Sustainable Development. 2015. Available: https://sdgs.un.org/2030agenda. Accessed: 12 August 2024.

[6] World Health Organization. Global Strategy for Women’s, Children’s and Adolescents’ Health (2016–2030): early childhood development. 10 May 2018. Available: https://apps.who.int/gb/ebwha/pdf_files/WHA71/A71_19Rev1-en.pdf. Accessed: 12 August 2024.

[7] Countdown to 2030 Collaboration Countdown to 2030: tracking progress towards universal coverage for reproductive, maternal, newborn, and child health. Lancet. 2018;391:1538–48. 10.1016/S0140-6736(18)30104-1 29395268

[8] Main page. dhis2. 2025. Available: https://dhis2.org/

[9] Lemma S Janson A Persson LÅ Wickremasinghe D Källestål C Improving quality and use of routine health information system data in low- and middle-income countries: A scoping review. PLoS One. 2020;15:e0239683. 10.1371/journal.pone.0239683 33031406 PMC7544093

[10] Kumar M Gotz D Nutley T Smith JB Research gaps in routine health information system design barriers to data quality and use in low- and middle-income countries: A literature review. Int J Health Plann Manage. 2018;33:e1–9. 10.1002/hpm.2447 28766742

[11] Simba D Sukums F Kumalija C Asiimwe SE Pothepragada SK Githendu PW Perceived Usefulness, Competency, and Associated Factors in Using District Health Information System Data Among District Health Managers in Tanzania: Cross-sectional Study. JMIR Form Res. 2022;6:e29469. 10.2196/29469 35604763 PMC9171597

[12] Mekuria S Adem HA Ayele BH Musa I Enyew DB Routine health information system utilization and associated factors among health professionals in public health facilities in Dire Dawa, eastern Ethiopia: A cross-sectional study. Digit Health. 2023;9:20552076231203914. 10.1177/20552076231203914 37808236 PMC10552451

[13] Aqil A Lippeveld T Hozumi D PRISM framework: a paradigm shift for designing, strengthening and evaluating routine health information systems. Health Policy Plan. 2009;24:217–28. 10.1093/heapol/czp010 19304786 PMC2670976

[14] von Elm E Altman DG Egger M Pocock SJ Gøtzsche PC Vandenbroucke JP The Strengthening the Reporting of Observational Studies in Epidemiology (STROBE) statement: guidelines for reporting observational studies. J Clin Epidemiol. 2008;61:344-9. 10.1016/j.jclinepi.2007.11.008 18313558

[15] O’Brien BC Harris IB Beckman TJ Reed DA Cook DA Standards for reporting qualitative research: a synthesis of recommendations. Acad Med. 2014;89:1245–51. 10.1097/ACM.0000000000000388 24979285

[16] Data for Impact. EWEN-MINSMI and EN-MINI Tools for Routine Health Information Systems. Available: https://www.data4impactproject.org/resources/en-mini-tools/. Accessed: 14 August 2024.

[17] Data for Impact. Every Newborn-Measurement Improvement for Newborn & Stillbirth Indicators EN-MINI-PRISM Tools for Routine Health Information Systems Internet. Chapel Hill, NC, USA: Data for Impact; 2023. Available: https://www.data4impactproject.org/wp-content/uploads/2022/02/EN-MINI-PRISM_Tools_1-6_220223.pdf. Accessed: 18 August 2024.

[18] MEASURE Evaluation. PRISM: Performance of Routine Information System Management Series. Available: https://www.measureevaluation.org/prism.html. Accessed: 18 August 2024.

[19] Mariani I Abathun E Mouhamadou O Minja J Kanamura RM Tognon F Organizational and management factors and related end-users’ perspectives relevant to newborn and stillbirth data at different levels of the health system: findings of the IMPULSE study in Uganda, Ethiopia, Tanzania and Central African Republic. J Glob Health. Forthcoming 2025. 10.1016/j.jclinepi.2007.11.008 41264542 PMC12634022

[20] Chrysantina A, Sæb J. Assessing User-Designed Dashboards: A Case for Developing Data Visualization Competency. In: Nielsen P, Kimaro HC, editors. Information and Communication Technologies for Development. Strengthening Southern-Driven Cooperation as a Catalyst for ICT4D. 15th IFIP WG 9.4 International Conference on Social Implications of Computers in Developing Countries; 2019 May 1-3; Daar es Salaam, Tanzania. Cham, Switzerland: Springer; 2019. p. 448-459.

[21] Byrne E Sæbø JI Routine use of DHIS2 data: a scoping review. BMC Health Serv Res. 2022;22:1234. 10.1186/s12913-022-08598-8 36203141 PMC9535952

[22] Asah FN Creating a “Community of Information Practice” for improved routine health data management in Resource Constrained Setting: The case of Mbingo Primary Healthcare facility, South Africa. Electron J Inf Syst Dev Ctries. 2021;87:e12178. 10.1002/isd2.12178

[23] MEASURE Evaluation. Barriers to Use of Health Data in Low- and Middle-Income Countries: A Review of the Literature [working paper]. MEASURE Evaluation: Chapel Hill, NC, USA: 2018. Available: https://www.measureevaluation.org/resources/publications/wp-18-211.html. Accessed: 18 August 2024.

[24] World Health Organization. Report of the 2nd meeting of the network for improving quality of care for maternal, newborn & child health. Geneva, Switzerland: World Health Organization; 2019. Available: https://cdn.who.int/media/docs/default-source/mca-documents/qoc/quality-of-care-network-documents/2nd-network-meeting-report—final-with-cover.pdf?sfvrsn=b3bc0499_1. Accessed: 18 August 2024.

[25] Kousiakis S, Sanner TA. Knowledge Mobilization through Co-Creation. gpekix. 23 June 2023. Available: https://www.gpekix.org/blog/knowledge-mobilization-through-co-creation. Accessed: 18 August 2024.

[26] Longworth GR Erikowa-Orighoye O Anieto EM Agnello DM Zapata-Restrepo JR Masquillier C Conducting co-creation for public health in low and middle-income countries: a systematic review and key informant perspectives on implementation barriers and facilitators. Global Health. 2024;20:9. 10.1186/s12992-024-01014-2 38233942 PMC10795424

[27] World Health Organization. Global strategy on human resources for health: Workforce 2030. Geneva, Switzerland: World Health Organization; 2020. Available: https://www.who.int/publications/i/item/9789241511131. Accessed: 18 August 2024.

[28] World Health Organization. Health workforce. 2025. Available: https://www.who.int/health-topics/health-workforce#tab=tab_1. Accessed: 18 August 2024.

[29] World Health Organization. WHA 75.17: Human Resources for Health. 28 May 2022. Available: https://apps.who.int/gb/ebwha/pdf_files/WHA75/A75_R17-en.pdf. Accessed: 18 August 2024.

[30] Chrysantina A Sæbø J Kaasbøll JJ Introducing online training for health staff: An institutional perspective. Electron J Inf Syst Dev Ctries. 2022;88:e12233. 10.1002/isd2.12233

[31] Dehnavieh R Haghdoost A Khosravi A Hoseinabadi F Rahimi H Poursheikhali A The District Health Information System (DHIS2): A literature review and meta-synthesis of its strengths and operational challenges based on the experiences of 11 countries. Health Inf Manag. 2019;48:62–75. 10.1177/1833358318777713 29898604

[32] Sahay S Rashidian A Doctor HV Challenges and opportunities of using DHIS2 to strengthen health information systems in the Eastern Mediterranean Region: A regional approach. Electron J Inf Syst Dev Ctries. 2020;86:e12108. 10.1002/isd2.12108

[33] Kaihlanen AM Elovainio M Virtanen L Kinnunen UM Vehko T Saranto K Nursing informatics competence profiles and perceptions of health information system usefulness among registered nurses: A latent profile analysis. J Adv Nurs. 2023;79:4022-33. 10.1111/jan.15718 37243421

[34] Board on Health Sciences Policy; Institute of Medicine. National Academies of Sciences, Engineering, and Medicine. Global Health Risk Framework: Resilient and Sustainable Health Systems to Respond to Global Infectious Disease Outbreaks: Workshop Summary. Washington, D.C., USA: National Academies Press (US); 2016. 27308691

[35] Van de Mortel TF Faking It: Social Desirability Response Bias in Self-report Research. Aust J Adv Nurs. 2008;25:40–8.

