# Supplementary material for: Users’ capabilities related to the electronic RHIS for newborn and stillbirth indicators: quantitative and qualitative findings of the IMPULSE study across 151 sites in the Central African Republic, Ethiopia, Tanzania, and Uganda
Source: J Glob Health. 2025 Nov 21;15:04239. doi: 10.7189/jogh.15.04239 (PMC12634022; doi:10.7189/jogh.15.04239)
Supplement: Online Supplementary Document [file jogh-15-04239-s001.pdf]

Supplment to: Mouhamadou O, Cora LG, Minja J, Abathun F, Kananura RM, Ayele M, Tognon F, Putoto G, Sæbø J, Mariani I, Geremia S, Dalena P, Shamba D, Lazzerini M. Users' capabilities related to the electronic RHIS for newborn and stillbirth indicators: quantitative and qualitative findings of the IMPULSE study across 151 sites in the Central African Republic, Ethiopia, Tanzania, and Uganda. J Glob Health. 2025;15:04239.

Online Supplementary Document

Table of contents

TABLES

Table S1. The Strengthening the Reporting of Observational Studies (STROBE) Checklist 1
Table S2. Standards for Reporting Qualitative Research (SRQR) 2
Table S3. Distribution of the facilities in the different regions 3
Table S4. Characteristics of the regions 4
Table S5. Sampling criteria 5
Table S6. Distribution of the facilities in the different countries 6
Table S7. Characteristics of the sample 7
Table S8. Reporting capabilities 8
Table S9. Calculation capabilities 11
Table S10. Data analysis capabilities 15
Table S11. Visualization capabilities 16
Table S12. Practical skills useful for the use of the eRHIS 18
Table S13. Users' perspective regarding the eRHIS 21
Table S14. Reporting capabilities by facility type 22
Table S15. Calculation capabilities by facility type 25
Table S16. Data analysis capabilities by facility type 33
Table S17. Visualization capabilities by facility type 35
Table S18. Practical skills useful for the use of the eRHIS by facility type 37
Table S19. Users' perspective regarding the eRHIS by facility type 46
Table S20. Reporting capabilities by region 48
Table S21. Calculation capabilities by region 52
Table S22. Data analysis capabilities by region 55
Table S23. Visualization capabilities by region 58
Table S24. Users' perspective by region 61
Table S25. Open question 63
Table S26. Significant examples of comments for each major team emerged in the thematic analysis 73

FIGURES

Figure S1. PRISM conceptual framework 75
Figure S2.1. Capabilities of the eRHIS users in Central African Republic, stratified by facility type 76
Figure S2.2. Capabilities of the eRHIS users in Ethiopia, stratified by facility type 77
Figure S2.3. Capabilities of the eRHIS users in Tanzania, stratified by facility type 78
Figure S2.4. Capabilities of the eRHIS users in Uganda, stratified by facility type 79
Figure S3.1. Practical skills in Central African Republic, stratified by facility type 80
Figure S3.2. Practical skills in Ethiopia, stratified by facility type 81
Figure S3.3. Practical skills in Tanzania, stratified by facility type 82
Figure S3.4. Practical skills in Uganda, stratified by facility type 83
Figure S4.1. Users' perspective regarding the eRHIS in Central African Republic, stratified by facility type 84
Figure S4.2. Users' perspective regarding the eRHIS in Ethiopia, stratified by facility type 84
Figure S4.3. Users' perspective regarding the eRHIS in Tanzania, stratified by facility type 84
Figure S4.4. Users' perspective regarding the eRHIS in Uganda, stratified by facility type 84

Table S1. The Strengthening the Reporting of Observational Studies (STROBE) Checklist

	**Item No**	**Recommendation**	**Pages**
**Title and abstract**	1	(*b*) Provide in the abstract an informative and balanced summary of what was done and what was found	1-3
Introduction			
Background/rationale	2	Explain the scientific background and rationale for the investigation being reported	3-4
Objectives	3	State specific objectives, including any prespecified hypotheses	4
Methods			
Study design	4	Present key elements of study design early in the paper	5
Setting	5	Describe the setting, locations, and relevant dates, including periods of recruitment, exposure, follow-up, and data collection	5
Participants	6	(*a*) Give the eligibility criteria, and the sources and methods of selection of participants	5
Variables	7	Clearly define all outcomes, exposures, predictors, potential confounders, and effect modifiers. Give diagnostic criteria, if applicable	5
Data sources/ measurement	8*	For each variable of interest, give sources of data and details of methods of assessment (measurement). Describe comparability of assessment methods if there is more than one group	6-7
Bias	9	Describe any efforts to address potential sources of bias	6-7
Study size	10	Explain how the study size was arrived at	5
Quantitative variables	11	Explain how quantitative variables were handled in the analyses. If applicable, describe which groupings were chosen and why	8-9
Statistical methods	12	(*a*) Describe all statistical methods, including those used to control for confounding	8-9
Statistical methods	12	(*b*) Describe any methods used to examine subgroups and interactions	8-9
Results			
Participants	13*	(a) Report numbers of individuals at each stage of study—eg numbers potentially eligible, examined for eligibility, confirmed eligible, included in the study, completing follow-up, and analysed	9
Descriptive data	14*	(a) Give characteristics of study participants (eg demographic, clinical, social) and information on exposures and potential confounders	9
Outcome data	15*	Report numbers of outcome events or summary measures	
Main results	16	(*a*) Give unadjusted estimates and, if applicable, confounder-adjusted estimates and their precision (eg, 95% confidence interval). Make clear which confounders were adjusted for and why they were included	9-13
Other analyses	17	Report other analyses done—eg analyses of subgroups and interactions, and sensitivity analyses	12-13
Discussion			
Key results	18	Summarise key results with reference to study objectives	15-18
Limitations	19	Discuss limitations of the study, taking into account sources of potential bias or imprecision. Discuss both direction and magnitude of any potential bias	16
Interpretation	20	Give a cautious overall interpretation of results considering objectives, limitations, multiplicity of analyses, results from similar studies, and other relevant evidence	15-18
Generalisability	21	Discuss the generalisability (external validity) of the study results	17-18
Other information			
Funding	22	Give the source of funding and the role of the funders for the present study and, if applicable, for the original study on which the present article is based	19

Table S2. Standards for Reporting Qualitative Research (SRQR)*

Title and abstract	Page/line no(s).
**Title** - Concise description of the nature and topic of the study Identifying the study as qualitative or indicating the approach (e.g., ethnography, grounded theory) or data collection methods (e.g., interview, focus group) is recommended	1
**Abstract** - Summary of key elements of the study using the abstract format of the intended publication; typically includes background, purpose, methods, results, and conclusions	3
**Introduction**	
**Problem formulation** - Description and significance of the problem/phenomenon studied; review of relevant theory and empirical work; problem statement	4
**Purpose or research question** - Purpose of the study and specific objectives or questions	4-5
**Methods**	
**Qualitative approach and research paradigm** - Qualitative approach (e.g., ethnography, grounded theory, case study, phenomenology, narrative research) and guiding theory if appropriate; identifying the research paradigm (e.g., postpositivist, constructivist/ interpretivist) is also recommended; rationale**	5
**Researcher characteristics and reflexivity** - Researchers' characteristics that may influence the research, including personal attributes, qualifications/experience, relationship with participants, assumptions, and/or presuppositions; potential or actual interaction between researchers' characteristics and the research questions, approach, methods, results, and/or transferability	5
**Context** - Setting/site and salient contextual factors; rationale**	5
**Sampling strategy** - How and why research participants, documents, or events were selected; criteria for deciding when no further sampling was necessary (e.g., sampling saturation); rationale**	5
**Data collection methods** - Types of data collected; details of data collection procedures including (as appropriate) start and stop dates of data collection and analysis, iterative process, triangulation of sources/methods, and modification of procedures in response to evolving study findings; rationale**	6
**Data collection instruments and technologies** - Description of instruments (e.g., interview guides, questionnaires) and devices (e.g., audio recorders) used for data collection; if/how the instrument(s) changed over the course of the study	6-7
**Units of study** - Number and relevant characteristics of participants, documents, or events included in the study; level of participation (could be reported in results)	7-8
**Techniques to enhance trustworthiness** - Techniques to enhance trustworthiness and credibility of data analysis (e.g., member checking, audit trail, triangulation); rationale**	8
**Data processing** - Methods for processing data prior to and during analysis, including transcription, data entry, data management and security, verification of data integrity, data coding, and anonymization/de-identification of excerpts	8
**Data analysis** - Process by which inferences, themes, etc., were identified and developed, including the researchers involved in data analysis; usually references a specific paradigm or approach; rationale**	8-9
**Ethical issues pertaining to human subjects** - Documentation of approval by an appropriate ethics review board and participant consent, or explanation for lack thereof; other confidentiality and data security issues	9
**Results/findings**	
**Synthesis and interpretation** - Main findings (e.g., interpretations, inferences, and themes); might include development of a theory or model, or integration with prior research or theory	9-15
**Links to empirical data** - Evidence (e.g., quotes, field notes, text excerpts, photographs) to substantiate analytic findings	14
**Discussion**	
**Integration with prior work, implications, transferability, and contribution(s) to the field -** Short summary of main findings; explanation of how findings and conclusions connect to, support, elaborate on, or challenge conclusions of earlier scholarship; discussion of scope of application/generalizability; identification of unique contribution(s) to scholarship in a discipline or field	15-17
**Limitations** - Trustworthiness and limitations of findings	16
**Other**	
**Conflicts of interest** - Potential sources of influence or perceived influence on study conduct and conclusions; how these were managed	19
**Funding** - Sources of funding and other support; role of funders in data collection, interpretation, and reporting	19

*The authors created the SRQR by searching the literature to identify guidelines, reporting standards, and critical appraisal criteria for qualitative research; reviewing the reference lists of retrieved sources; and contacting experts to gain feedback. The SRQR aims to improve the transparency of all aspects of qualitative research by providing clear standards for reporting qualitative research.

**The rationale should briefly discuss the justification for choosing that theory, approach, method, or technique rather than other options available, the assumptions and limitations implicit in those choices, and how those choices influence study conclusions and transferability. As appropriate, the rationale for several items might be discussed together.

O'Brien BC, Harris IB, Beckman TJ, Reed DA, Cook DA. Standards for reporting qualitative research: a synthesis of recommendations. *Academic Medicine*, Vol. 89, No. 9 / Sept 2014. Available:
DOI: 10.1097/ACM.0000000000000388
http://www.equator-network.org/reporting-guidelines/srqr/

Table S3. Distribution of the facilities in the different regions

**Data offices**	Central African Republic			Ethiopia			Tanzania			Uganda			Overall
	Region name	N	%	Region name	N	%	Region name	N	%	Region name	N	%	
	N = 7			N = 11			N = 18			N = 20			
	Bangui C.A.	1	14.3	Addis Ababa C.A.	2	18.2	Dar es Salaam C.A.	1	5.6	Karamoja	8	40	
	Health region 1	1	14.3	Amhara-Gambella	2	18.2	Iringa	6	33.3	Lango	6	30	
	Health region 2	2	28.6	Oromia	3	27.3	Shinyanga	5	27.8	West-Nile	6	30	
	Health region 7	3	42.9	South Ethiopia-Sidama	4	36.4	Simiyu	6	33.3				
Overall		7	13		11	19.6		18	32.1		20	35.7	56
**Facilities**	Central African Republic			Ethiopia			Tanzania			Uganda			Overall
	Region name	N	%	Region name	N	%	Region name	N	%	Region name	N	%	
	N = 7			N = 11			N = 18			N = 20			
	Bangui C.A.	3	21.4	Addis Ababa C.A.	3	12.5	Dar es Salaam C.A.	2	7.1	Kampala C.A.	1	3.4	
	Health region 1	3	21.4	Amhara-Gambella	3	12.5	Iringa	10	35.7	Karamoja	8	27.6	
	Health region 2	4	28.6	Oromia	9	37.5	Shinyanga	8	28.6	Lango	9	31	
	Health region 7	4	28.6	South Ethiopia-Sidama	9	37.5	Simiyu	8	28.6	West-Nile	11	37.9	
Overall		14	14.7		24	25.3		28	29.5		29	30.5	95
Overall		21	13.9		35	23.2		46	30.5		49	32.5	151

C.A. – city administration

Table S4. Characteristics of the regions

**Setting**	Central African Republic				
	Bangui C.A.	Health region 1	Health region 2	Health region 7	Overall
Urban	3	3	3	4	13
Rural	0	0	1	0	1
Overall					14
	Ethiopia				
	Addis Ababa C.A.	Oromia	Amhara -Gambella	South Ethiopia -Sidama	Overall
Urban	3	6	3	6	18
Rural	0	3	0	3	6
Overall					24
	Tanzania				
	Dar es Salaam C.A.	Iringa	Shinyanga	Simiyu	Overall
Urban	2	3	2	1	8
Rural	0	7	6	7	20
Overall					28
	Uganda				
	Kampala C.A.	Lango	Karamoja	West-Nile	Overall
Urban	1	3	1	8	13
Rural	0	6	7	3	16
Overall					29

C.A. – city administration

Table S5. Sampling criteria

**Facility type**	**Criteria**
Health facilities	
3rd level of referral and/or National	1
In each region:	
3rd level of referral (Regional)	1
2nd level of referral (Subnational / District) Public	2
2nd level of referral (Subnational / District) Not For Profit *	1
2nd level of referral (Subnational / District) Private *	1
1st level of referral (Primary Hospital / Health Centre with CEmONC) Public	3
1st level of referral (Primary Hospital / Health Centre with CEmONC) Not For Profit *	1
1st level of referral (Primary Hospital / Health Centre with CEmONC) Private *	1-2
Data offices	
District /Subnational Health Office	all data offices related to selected facilities
Regional Health office	yes
Central Ministry of Health	yes

Only CEmONC facilities, except for CAR, where BEmONC facilities were included; * if existing and allowing
BEmONC - basic emergency obstetric and neonatal care, CAR – Central African Republic, CEmONC - comprehensive emergency obstetric and newborn care

Table S6. Distribution of the facilities in the different countries

**Data offices**		Central African Republic		Ethiopia		Tanzania		Uganda		Overall	
		N	%	N	%	N	%	N	%	N	%
	Central/regional health data office	1	4.7	5	14.3	4	8.7	0	0	10	17.9
	District/subnational health data office	6	28.6	6	17.1	14	30.4	20	40.8	46	82.1
	Overall	7	33.3	11	31.4	18	39.1	20	40.8	56	100
**Facilities**		Central African Republic		Ethiopia		Tanzania		Uganda		Overall	
	First level of referral health facility	7	33.3	11	31.4	9	19.6	12	24.5	39	41.1
	Second level of referral hospital	3	14.3	10	28.6	14	30.4	12	24.5	39	41.1
	Third level of referral hospital	4	19	3	8.6	5	10.9	5	10.2	17	17.9
	Overall	14	66.6	24	68.6	28	60.9	29	59.2	95	100
Overall		21	13.9	35	23.2	46	30.5	49	32.5	151	100

Table S7. Characteristics of the sample

**Setting**	Central African Republic		Ethiopia		Tanzania		Uganda		Overall	
	N	%	N	%	N	%	N	%	N	%
	N = 14		N = 24		N = 28		N = 29		N = 95	
Rural	1	7.1	6	25	20	71.4	16	55.2	43	45.3
Urban	13	92.9	18	75	8	28.6	13	44.8	52	54.7
Overall	14	14.7	24	25.3	28	29.5	29	30.5	95	100

**Managing authority**	Central African Republic		Ethiopia		Tanzania		Uganda		Overall	
	N	%	N	%	N	%	N	%	N	%
	N = 14		N = 24		N = 28		N = 29		N = 95	
Public	12	85.8	18	75	21	75	22	75.9	73	76.8
Private	1	7.1	5	20.8	3	10.7	0	0	9	9.5
Not for profit	1	7.1	1	4.2	4	14.3	7	24.1	13	13.7
Overall	14	14.7	24	25.3	28	29.5	29	30.5	95	100

Table S8. Reporting capabilities

Track report completeness using eRHIS	Facility type	Outcome	Central African Republic		Ethiopia		Tanzania		Uganda		Overall	
			N	%	N	%	N	%	N	%	N	%
			N = 21		N = 35		N = 46		N = 49		N = 151	
	Data offices	Yes	6	85.7	11	100	17	94.4	18	90	52	92.9
		No	1	14.3	0	0	1	5.6	2	10	4	7.1
	Facilities	Yes	1	7.1	10	41.7	24	85.7	26	89.7	61	64.2
		No	0	0	7	29.2	3	10.7	1	3.4	11	11.6
		NA	13	92.9	7	29.1	1	3.6	2	6.9	23	24.2

Capacity to generate summary reports	Facility type	Outcome	Central African Republic		Ethiopia		Tanzania		Uganda		Overall	
			N	%	N	%	N	%	N	%	N	%
			N = 21		N = 35		N = 46		N = 49		N = 151	
National/regional summary for a month	Data offices	Yes	1	14.3	6	54.5	16	88.9	12	60	35	62.5
		No	4	57.1	4	36.4	1	5.6	7	35	16	28.6
		NA	2	28.6	1	9.1	1	5.6	1	5	5	8.9
National/regional summary for a quarter	Data offices	Yes	1	14.3	7	63.6	16	88.9	12	60	36	64.3
		No	4	57.1	3	27.3	1	5.6	7	35	15	26.8
		NA	2	28.6	1	9.1	1	5.6	1	5	5	8.9
National/regional summary for a year	Data offices	Yes	1	14.3	7	63.6	16	88.9	12	60	36	64.3
		No	4	57.1	3	27.3	1	5.6	7	35	15	26.8
		NA	2	28.6	1	9.1	1	5.6	1	5	5	8.9
District summary for a month	Data offices	Yes	2	28.6	10	90.9	18	100	20	100	50	89.3
		No	5	71.4	1	9.1	0	0.0	0	0	6	10.7
		NA	0	0.0	0	0.0	0	0.0	0	0	0	0.0
District summary for a quarter	Data offices	Yes	2	28.6	10	90.9	18	100	20	100	50	89.3
		No	5	71.4	1	9.1	0	0.0	0	0	6	10.7
		NA	0	0.0	0	0.0	0	0.0	0	0	0	0.0
District summary for a year	Data offices	Yes	2	28.6	10	90.9	18	100	20	100	50	89.3
		No	5	71.4	1	9.1	0	0.0	0	0	6	10.7
		NA	0	0.0	0	0.0	0	0.0	0	0	0	0.0
Health facility summary for a month	Data offices	Yes	7	100	10	90.9	18	100	20	100	55	98.2
		No	0	0.0	1	9.1	0	0.0	0	0	1	1.8
		NA	0	0.0	0	0.0	0	0.0	0	0	0	0.0
Health facility summary for a quarter	Data offices	Yes	7	100	10	90.9	18	100	20	100	55	98.2
		No	0	0.0	1	9.1	0	0.0	0	0	1	1.8
		NA	0	0.0	0	0.0	0	0.0	0	0	0	0.0
Health facility summary for a year	Data offices	Yes	7	100	10	90.9	18	100	20	100	55	98.2
		No	0	0.0	1	9.1	0	0.0	0	0	1	1.8
		NA	0	0.0	0	0.0	0	0.0	0	0	0	0.0
Community-level SDP summary for a month	Data offices	Yes	0	0.0	7	63.6	1	5.6	10	50	18	32.1

		No	7	100	2	18.2	6	33.3	9	45	24	42.9
		NA	0	0.0	2	18.2	11	61.1	1	5	14	25.0
Community-level SDP summary for a quarter	Data offices	Yes	0	0.0	8	72.7	1	5.6	12	60	21	37.5
		No	7	100	1	9.1	6	33.3	7	35	21	37.5
		NA	0	0.0	2	18.2	11	61.1	1	5	14	25.0
Community-level SDP summary for a year	Data offices	Yes	0	0.0	8	72.7	1	5.6	12	60	21	37.5
		No	7	100.0	1	9.1	6	33.3	7	35	21	37.5
		NA	0	0.0	2	18.2	11	61.1	1	5	14	25.0
National/regional summary for a month	Facilities	Yes	0	0.0	3	12.5	21	75.0	11	37.9	35	36.8
		No	1	7.1	0	0.0	6	21.4	16	55.2	23	24.2
		NA	13	92.9	21	87.5	1	3.6	2	6.9	37	38.9
National/regional summary for a quarter	Facilities	Yes	0	0.0	3	12.5	21	75.0	11	37.9	35	36.8
		NO	1	7.1	0	0.0	6	21.4	16	55.2	23	24.2
		NA	13	92.9	21	87.5	1	3.6	2	6.9	37	38.9
National/regional summary for a year	Facilities	Yes	0	0.0	3	12.5	21	75.0	11	37.9	35	36.8
		No	1	7.1	0	0.0	6	21.4	16	55.2	23	24.2
		NA	13	92.9	21	87.5	1	3.6	2	6.9	37	38.9
District summary for a month	Facilities	Yes	0	0.0	6	25.0	21	75.0	22	75.9	49	51.6
		No	1	7.1	1	4.2	6	21.4	4	13.8	12	12.6
		NA	13	92.9	17	70.8	1	3.6	3	10.3	34	35.8
District summary for a quarter	Facilities	Yes	0	0.0	6	25.0	21	75.0	22	75.9	49	51.6
		No	1	7.1	1	4.2	6	21.4	4	13.8	12	12.6
		NA	13	92.9	17	70.8	1	3.6	3	10.3	34	35.8
District summary for a year	Facilities	Yes	0	0.0	6	25.0	21	75.0	22	75.9	49	51.6
		No	1	7.1	1	4.2	6	21.4	4	13.8	12	12.6
		NA	13	92.9	17	70.8	1	3.6	3	10.3	34	35.8
Health facility summary for a month	Facilities	Yes	4	28.6	23	95.8	27	96.4	28	96.6	82	86.3
		No	0	0.0	0	0.0	0	0.0	0	0.0	0	0.0
		NA	10	71.4	1	4.2	1	3.6	1	3.4	13	13.7
Health facility summary for a quarter	Facilities	Yes	4	28.6	22	91.7	27	96.4	28	96.6	81	85.3
		No	0	0.0	0	0.0	0	0.0	0	0.0	0	0.0
		NA	10	71.4	1	4.2	1	3.6	1	3.4	13	13.7
Health facility summary for a year	Facilities	Yes	4	28.6	22	91.7	26	92.9	28	96.6	80	84.2
		No	0	0.0	0	0.0	1	3.6	0	0.0	1	1.1
		NA	10	71.4	1	4.2	1	3.6	1	3.4	13	13.7
Community-level SDP summary for a month	Facilities	Yes	1	7.1	5	20.8	3	10.7	13	44.8	22	23.2
		No	0	0.0	7	29.2	10	35.7	14	48.3	31	32.6
		NA	13	92.9	12	50.0	15	53.6	2	6.9	42	44.2
Community-level SDP summary for a quarter	Facilities	Yes	1	7.1	5	20.8	3	10.7	17	58.6	26	27.4
		No	0	0.0	7	29.2	10	35.7	10	34.5	27	28.4
		NA	13	92.9	12	50.0	15	53.6	2	6.9	42	44.2
Community-level SDP summary for a year	Facilities	Yes	1	7.1	5	20.8	3	10.7	12	41.4	21	22.1
		No	0	0.0	7	29.2	10	35.7	15	51.7	32	33.7
		NA	13	92.9	12	50.0	15	53.6	2	6.9	42	44.2

**PRISM index**	**Facility type**	Central African Republic	Ethiopia	Tanzania	Uganda	Overall
		N = 21	N = 35	N = 46	N = 49	N = 151
Track report completeness using eRHIS	Data offices	85.70	100	94.4	90	92.9
Track report completeness using eRHIS	Facilities	7.10	41.7	85.7	89.7	64.2
**Capacity to generate summary reports**						
National/regional summary for a year	Data offices	14.3	63.6	88.9	60	64.3
District summary for a year	Data offices	28.6	90.9	100	100	89.3
Health facility summary for a year	Data offices	100	90.9	100	100	98.2
Community-level SDP summary for a year	Data offices	0.0	72.7	5.6	60	37.5
National/regional summary for a year	Facilities	0.0	12.5	75.0	37.9	36.8
District summary for a year	Facilities	0.0	25.0	75.0	75.9	51.6
Health facility summary for a year	Facilities	28.6	91.7	92.9	96.6	84.2
Community-level SDP summary for a year	Facilities	7.1	20.8	10.7	41.4	22.1
**Number of key indicators >80**		2	4	6	5	4
**Percentage of key indicators >80**		20	40	60	50	40

In yellow are marked the indicators that contributed to the calculation via average of the PRISM indexes

eRHIS – electronic routine health information system, NA – not answered, PRISM - performance of routine information system management, SDP – self-determination program

Table S9. Calculation capabilities

Percentage of staff demonstrating the use of data analysis features	Facility type	Outcome	Central African Republic		Ethiopia		Tanzania		Uganda		Overall	
			N	%	N	%	N	%	N	%	N	%
			N = 21		N = 35		N = 46		N = 49		N = 151	
Neonatal mortality rate - National	Data offices	Yes	1	14.3	4	36.4	6	33.3	9	45	20	35.7
		No	4	57.1	4	36.4	10	55.6	9	45	27	48.2
		NA	2	28.6	3	27.3	2	11.1	2	10	9	16.1
Neonatal mortality rate Neonatal mortality rate - Region	Data offices	Yes	1	14.3	7	63.6	6	33.3	13	65	27	48.2
		No	4	57.1	3	27.3	10	55.6	5	25	22	39.3
		NA	2	28.6	1	9.1	2	11.1	2	10	7	12.5
Neonatal mortality rate - District	Data offices	Yes	3	42.9	10	90.9	10	55.6	20	100	43	76.8
		No	4	57.1	1	9.1	8	44.4	0	0	13	23.2
Neonatal mortality rate - Health facility	Data offices	Yes	6	85.7	9	81.8	10	55.6	20	100	45	80.4
		No	1	14.3	2	18.2	8	44.4	0	0	11	19.6
Neonatal mortality rate - Community-level SDP	Data offices	Yes	0	0.0	5	45.5	0	0.0	6	30	11	19.6
		No	6	85.7	3	27.3	6	33.3	13	65	28	50.0
		NA	1	14.3	3	27.3	12	66.7	1	5	17	30.4
Stillbirth rate - National	Data offices	Yes	1	14.3	4	36.4	7	38.9	9	45	21	37.5
		No	4	57.1	4	36.4	9	50.0	8	40	25	44.6
		NA	2	28.6	3	27.3	2	11.1	3	15	10	17.9
Stillbirth rate - Region	Data offices	Yes	1	14.3	7	63.6	7	38.9	12	60	27	48.2
		No	4	57.1	3	27.3	9	50.0	6	30	22	39.3
		NA	2	28.6	1	9.1	2	11.1	2	10	7	12.5
Stillbirth rate - District	Data offices	Yes	3	42.9	10	90.9	11	61.1	20	100	44	78.6
		No	4	57.1	1	9.1	7	38.9	0	0	12	21.4
Stillbirth rate - Health facility	Data offices	Yes	6	85.7	9	81.8	11	61.1	20	100	46	82.1
		No	1	14.3	2	18.2	7	38.9	0	0	10	17.9
Stillbirth rate - Community-level SDP	Data offices	Yes	0	0.0	5	45.5	0	0.0	6	30	11	19.6
		No	6	85.7	3	27.3	6	33.3	13	65	28	50.0
		NA	1	14.3	3	27.3	12	66.7	1	5	17	30.4
Low birth weight rate - National	Data offices	Yes	1	14.3	3	27.3	7	38.9	9	45	20	35.7
		No	4	57.1	5	45.5	9	50.0	8	40	26	46.4
		NA	2	28.6	3	27.3	2	11.1	3	15	10	17.9
Low birth weight rate - Region	Data offices	Yes	1	14.3	6	54.5	7	38.9	12	60	26	46.4
		No	4	57.1	4	36.4	9	50.0	6	30	23	41.1
		NA	2	28.6	1	9.1	2	11.1	2	10	7	12.5
Low birth weight rate- District	Data offices	Yes	3	42.9	9	81.8	11	61.1	20	100	43	76.8

		No	4	57.1	2	18.2	7	38.9	0	0	13	23.2
Low birth weight rate - Health facility	Data offices	Yes	6	85.7	8	72.7	11	61.1	20	100	45	80.4
		No	1	14.3	3	27.3	3	16.7	0	0	7	12.5
Low birth weight rate - Community-level SDP	Data offices	Yes	0	0.0	4	36.4	0	0.0	6	30	10	17.9
		No	6	85.7	4	36.4	6	33.3	13	65	29	51.8
		NA	1	14.3	3	27.3	12	66.7	1	5	17	30.4
Neonatal mortality rate - National	Facilities	Yes	0	0.0	3	12.5	6	21.4	5	17.2	14	14.7
		No	1	7.1	1	4.2	21	75.0	20	69.0	43	45.3
		NA	13	92.9	20	83.3	1	3.6	4	13.8	38	40.0
Neonatal mortality rate - Region	Facilities	Yes	0	0.0	3	12.5	6	21.4	9	31.0	18	18.9
		No	1	7.1	1	4.2	21	75.0	17	58.6	40	42.1
		NA	13	92.9	20	83.3	1	3.6	3	10.3	37	38.9
Neonatal mortality rate - District	Facilities	Yes	0	0.0	4	16.7	6	21.4	19	65.5	29	30.5
		No	1	7.1	3	12.5	21	75.0	7	24.1	32	33.7
		NA	13	92.9	17	70.8	1	3.6	3	10.3	34	35.8
Neonatal mortality rate - Health facility	Facilities	Yes	4	28.6	14	58.3	11	39.3	25	86.2	54	56.8
		No	0	0.0	9	37.5	16	57.1	3	10.3	28	29.5
		NA	10	71.4	1	4.2	1	3.6	1	3.4	13	13.7
Neonatal mortality rate - Community-level SDP	Facilities	Yes	0	0.0	1	4.2	0	0.0	11	37.9	12	12.6
		No	1	7.1	9	37.5	12	42.9	15	51.7	37	38.9
		NA	13	92.9	14	58.3	16	57.1	3	10.3	46	48.4
Stillbirth rate - National	Facilities	Yes	0	0.0	3	12.5	6	21.4	5	17.2	14	14.7
		No	1	7.1	1	4.2	21	75.0	20	69.0	43	45.3
		NA	13	92.9	20	83.3	1	3.6	4	13.8	38	40.0
Stillbirth rate - Region	Facilities	Yes	0	0.0	3	12.5	6	21.4	9	31.0	18	18.9
		No	1	7.1	1	4.2	21	75.0	17	58.6	40	42.1
		NA	13	92.9	20	83.3	1	3.6	3	10.3	37	38.9
Stillbirth rate - District	Facilities	Yes	0	0.0	4	16.7	6	21.4	19	65.5	29	30.5
		No	1	7.1	3	12.5	21	75.0	7	24.1	32	33.7
		NA	13	92.9	17	70.8	1	3.6	3	10.3	34	35.8
Stillbirth rate - Health facility	Facilities	Yes	3	21.4	15	62.5	12	42.9	25	86.2	55	57.9
		No	0	0.0	8	33.3	15	53.6	3	10.3	26	27.4
		NA	11	78.6	1	4.2	1	3.6	1	3.4	14	14.7
Stillbirth rate - Community-level SDP	Facilities	Yes	0	0.0	1	4.2	0	0.0	11	37.9	12	12.6
		No	1	7.1	9	37.5	12	42.9	14	48.3	36	37.9
		NA	13	92.9	14	58.3	16	57.1	4	13.8	47	49.5
Low birth weight rate - National	Facilities	Yes	0	0.0	3	12.5	6	21.4	5	17.2	14	14.7
		No	1	7.1	1	4.2	21	75.0	20	69.0	43	45.3
		NA	13	92.9	20	83.3	1	3.6	4	13.8	38	40.0
Low birth weight rate - Region	Facilities	Yes	0	0.0	3	12.5	6	21.4	9	31.0	18	18.9
		No	1	7.1	1	4.2	21	75.0	17	58.6	40	42.1
		NA	13	92.9	20	83.3	1	3.6	3	10.3	37	38.9

Low birth weight rate - District	Facilities	Yes	0	0.0	4	16.7	6	21.4	19	65.5	29	30.5
		No	1	7.1	3	12.5	21	75.0	7	24.1	32	33.7
		NA	13	92.9	17	70.8	1	3.6	3	10.3	34	35.8
Low birth weight rate - Health facility	Facilities	Yes	4	28.6	13	54.2	12	42.9	24	82.8	53	55.8
		No	0	0.0	10	41.7	15	53.6	4	13.8	29	30.5
		NA	10	71.4	1	4.2	1	3.6	1	3.4	13	13.7
Low birth weight rate - Community-level SDP	Facilities	Yes	0	0.0	1	4.2	0	0.0	10	34.5	11	11.6
		No	1	7.1	9	37.5	12	42.9	15	51.7	37	38.9
		NA	13	92.9	14	58.3	16	57.1	4	13.8	47	49.5

**PRISM index**	**Facility type**	Central African Republic	Ethiopia	Tanzania	Uganda	Overall
		N = 21	N = 35	N = 46	N = 49	N = 151
Neonatal mortality rate - National	Data offices	14.3	36.4	33.3	45.0	35.7
Neonatal mortality rate - Region	Data offices	14.3	63.6	33.3	65.0	48.2
Neonatal mortality rate - District	Data offices	42.9	90.9	55.6	100.0	76.8
Neonatal mortality rate - Health facility	Data offices	85.7	81.8	55.6	100.0	80.4
Neonatal mortality rate - Community-level SDP	Data offices	0.0	45.5	0.0	30.0	19.6
Stillbirth rate - National	Data offices	14.3	36.4	38.9	45.0	37.5
Stillbirth rate - Region	Data offices	14.3	63.6	38.9	60.0	48.2
Stillbirth rate - District	Data offices	42.9	90.9	61.1	100.0	78.6
Stillbirth rate - Health facility	Data offices	85.7	81.8	61.1	100.0	82.1
Stillbirth rate - Community-level SDP	Data offices	0.0	45.5	0.0	30.0	19.6
Low birth weight rate- National	Data offices	14.3	27.3	38.9	45.0	35.7
Low birth weight rate - Region	Data offices	14.3	54.5	38.9	60.0	46.4
Low birth weight rate - District	Data offices	42.9	81.8	61.1	100.0	76.8
Low birth weight rate - Health facility	Data offices	85.7	72.7	61.1	100.0	80.4
Low birth weight rate - Community-level SDP	Data offices	0.0	36.4	0.0	30.0	17.9
Neonatal mortality rate - National	Facilities	0.0	12.5	21.4	17.2	14.7
Neonatal mortality rate - Region	Facilities	0.0	12.5	21.4	31.0	18.9
Neonatal mortality rate - District	Facilities	0.0	16.7	21.4	65.5	30.5
Neonatal mortality rate - Health facility	Facilities	28.6	58.3	39.3	86.2	56.8
Neonatal mortality rate - Community-level SDP	Facilities	0.0	4.2	0.0	37.9	12.6
Stillbirth rate - National	Facilities	0.0	12.5	21.4	17.2	14.7
Stillbirth rate - Region	Facilities	0.0	12.5	21.4	31.0	18.9
Stillbirth rate- District	Facilities	0.0	16.7	21.4	65.5	30.5
Stillbirth rate - Health facility	Facilities	21.4	62.5	42.9	86.2	57.9

Stillbirth rate - Community-level SDP	Facilities	0.0	4.2	0.0	37.9	12.6
Low birth weight rate - National	Facilities	0.0	12.5	21.4	17.2	14.7
Low birth weight rate - Region	Facilities	0.0	12.5	21.4	31.0	18.9
Low birth weight rate - District	Facilities	0.0	16.7	21.4	65.5	30.5
Low birth weight rate - Health facility	Facilities	28.6	54.2	42.9	82.8	55.8
Low birth weight rate - Community-level SDP	Facilities	0.0	4.2	0.0	34.5	11.6

**Aggregated PRISM index**	**Facility type**	Central African Republic	Ethiopia	Tanzania	Uganda	Overall
Neonatal mortality rate	Data offices	31.4	63.6	35.6	68.0	52.1
Stillbirth rate	Data offices	31.4	63.6	40.0	67.0	53.2
Low birth weight rate	Data offices	31.4	54.5	40.0	67.0	51.4
Neonatal mortality rate	Facilities	5.7	20.8	20.7	47.6	26.7
Stillbirth rate	Facilities	4.3	21.7	21.4	47.6	26.9
Low birth weight rate	Facilities	5.7	20.0	21.4	46.2	26.3
**Number of key indicators >80**		0	0	0	0	0
**Percentage of key indicators >80**		0	0	0	0	0

In yellow are marked the indicators that contributed to the calculation via average of the PRISM indexes

NA – not answered, PRISM - performance of routine information system management, SDP – self-determination program

Table S10. Data analysis capabilities

Data analysis features	Facility type	Outcome	Central African Republic		Ethiopia		Tanzania		Uganda		Overall	
			N	%	N	%	N	%	N	%	N	%
			N = 21		N = 35		N = 46		N = 49		N = 151	
Major causes neonatal mortality	Data offices	Yes	0	0	0	0	11	61.1	17	85	28	50.0
		No	7	100	11	100	7	38.9	3	15	28	50.0
	Facilities	Yes	1	7.1	5	20.8	7	25.0	21	72.4	34	35.8
		No	3	21.4	17	70.8	20	71.4	7	24.1	47	49.5
		NA	10	71.4	2	8.3	1	3.6	1	3.4	14	14.7
Major causes neonatal morbidity	Data offices	Yes	0	0	2	18.1	9	50.0	19	95	30	53.6
		No	7	100	9	81.9	8	44.4	1	5	25	44.6
		NA	0	0	0	0	1	5.6	0	0	1	1.8
	Facilities	Yes	0	0.0	6	25.0	6	21.4	19	65.5	31	32.6
		No	3	21.4	16	66.7	21	75.0	9	31.0	49	51.6
		NA	11	78.6	2	8.3	1	3.6	1	3.4	15	15.8
Data disaggregation	Data offices	Yes	2	28.5	0	0	8	44.4	15	75	25	44.6
		No	5	71.5	11	100	10	55.6	5	25	31	55.4
	Facilities	Yes	2	14.3	2	8.3	10	35.7	14	48.3	28	29.5
		No	1	7.1	20	83.3	17	60.7	14	48.3	52	54.7
		NA	11	78.6	2	8.3	1	3.6	1	3.4	15	15.8

PRISM index	Facility type	Central African Republic	Ethiopia	Tanzania	Uganda	Overall
		N = 21	N = 35	N = 46	N = 49	N = 151
Major causes neonatal mortality	Data offices	0	0	61.1	85	50.0
Major causes neonatal mortality	Facilities	7.1	20.8	25.0	72.4	35.8
Major causes neonatal morbidity	Data offices	0	18.1	50.0	95	53.6
Major causes neonatal morbidity	Facilities	0.0	25.0	21.4	65.5	32.6
Data disaggregation	Data offices	28.5	0	44.4	75	44.6
Data disaggregation	Facilities	14.3	8.3	35.7	48.3	29.5
**Number of key indicators >80**		0	0	0	2	0
**Percentage of key indicators >80**		0	0	0	33.3	0

In yellow are marked the indicators that contributed to the calculation via average of the PRISM indexes

NA – not answered, PRISM - performance of routine information system management

Table S11. Visualization capabilities

Percentage of staff able to use the data visualization features of the eRHIS to analyse and present data in graphs and maps	Facility type	Outcome	Central African Republic		Ethiopia		Tanzania		Uganda		Overall	
			N	%	N	%	N	%	N	%	N	%
			N = 21		N = 25		N = 46		N = 49		N = 151	
Indicator 1 - Time trend graphs	Data offices	Yes	4	57.1	7	63.6	10	55.6	20	100	41	73.2
		No	3	42.9	4	36.4	8	44.4	0	0	15	26.8
Indicator 1 - Bar graphs for comparing facilities, districts, or regions	Data offices	Yes	2	28.6	7	63.6	10	55.6	20	100	39	69.6
		No	4	57.1	4	36.4	8	44.4	0	0	16	28.6
		NA	1	14.3	0	0.0	0	0.0	0	0	1	1.8
Indicator 1 - Thematic maps, by region, district, or health facility	Data offices	Yes	2	28.6	6	54.5	9	50.0	14	70	31	55.4
		No	4	57.1	3	27.3	9	50.0	6	30	22	39.3
		NA	1	14.3	2	18.2	0	0.0	0	0	3	5.4
Indicator 2 - Time trend graphs	Data offices	Yes	4	57.1	4	36.4	10	55.6	20	100	38	67.9
		No	3	42.9	7	63.6	8	44.4	0	0	18	32.1
Indicator 2 - Bar graphs for comparing facilities, districts, or regions	Data offices	Yes	2	28.6	4	36.4	10	55.6	20	100	36	64.3
		No	4	57.1	7	63.6	8	44.4	0	0	19	33.9
		NA	1	14.3	0	0.0	0	0.0	0	0	1	1.8
Indicator 2 - Thematic maps, by region, district, or health facility	Data offices	Yes	2	28.6	4	36.4	9	50.0	14	70	29	51.8
		No	4	57.1	6	54.5	9	50.0	6	30	25	44.6
		NA	1	14.3	1	9.1	0	0.0	0	0	2	3.6
Indicator 1 - Time trend graphs	Facilities	Yes	3	21.4	13	54.2	11	39.3	23	79.3	50	52.6
		No	0	0.0	10	41.7	16	57.1	5	17.2	31	32.6
		NA	11	78.6	1	4.2	1	3.6	1	3.4	14	14.7
Indicator 1 - Bar graphs for comparing facilities, districts, or regions	Facilities	Yes	0	0.0	11	45.8	11	39.3	22	75.9	44	46.3
		No	0	0.0	12	50.0	16	57.1	5	17.2	33	34.7
		NA	14	100.0	1	4.2	1	3.6	2	6.9	18	18.9
Indicator 1 - Thematic maps, by region, district, or health facility	Facilities	Yes	0	0.0	9	37.5	9	32.1	14	48.3	32	33.7
		No	0	0.0	14	58.3	17	60.7	13	44.8	44	46.3
		NA	14	100.0	1	4.2	2	7.1	2	6.9	19	20.0
Indicator 2 - Time trend graphs	Facilities	Yes	2	14.3	11	45.8	11	39.3	23	79.3	47	49.5
		No	1	7.1	10	41.7	16	57.1	5	17.2	32	33.7
		NA	11	78.6	3	12.5	1	3.6	1	3.4	16	16.8

Indicator 2 - Bar graphs for comparing facilities, districts, or regions	Facilities	Yes	0	0.0	9	37.5	12	42.9	22	75.9	43	45.3
		No	0	0.0	12	50.0	15	53.6	5	17.2	32	33.7
		NA	14	100.0	3	12.5	1	3.6	2	6.9	20	21.1
Indicator 2 - Thematic maps, by region, district, or health facility	Facilities	Yes	0	0.0	8	33.3	7	25.0	13	44.8	28	29.5
		No	0	0.0	13	54.2	18	64.3	14	48.3	45	47.4
		NA	14	100.0	3	12.5	3	10.7	2	6.9	22	23.2

**PRISM index**	**Facility type**	Central African Republic	Ethiopia	Tanzania	Uganda	Overall
		N = 21	N = 25	N = 46	N = 49	N = 151
Indicator 1 - Time trend graphs	Data offices	57.1	63.6	55.6	100.0	73.2
Indicator 1 - Bar graphs for comparing facilities, districts, or regions	Data offices	28.6	63.6	55.6	100.0	69.6
Indicator 1 - Thematic maps, by region, district, or health facility	Data offices	28.6	54.5	50.0	70.0	55.4
Indicator 1 - Time trend graphs	Facilities	21.4	54.2	39.3	79.3	52.6
Indicator 1 - Bar graphs for comparing facilities, districts, or regions	Facilities	0.0	45.8	39.3	75.9	46.3
Indicator 1 - Thematic maps, by region, district, or health facility	Facilities	0.0	37.5	32.1	48.3	33.7
**Number of key indicators >80**		0	0	0	2	0
**Percentage of key indicators >80**		0	0	0	33.3	0

In yellow are marked the indicators that contributed to the calculation via average of the PRISM indexes

eRHIS – electronic routine health information system, NA – not answered, PRISM - performance of routine information system management

Table S12. Practical skills useful for the use of the eRHIS

	**Facility type**	Central African Republic		Ethiopia		Tanzania		Uganda		Overall	
		Observed score	Max score	Observed score	Max score	Observed score	Max score	Observed score	Max score	Observed score	Max score
		N = 20		N = 98		N = 81		N = 114		N = 313	
Calculate the percentage of pregnant mothers in the district attending ANC in the current period	Data offices	3	7	19	21	15	29	22	23	59	80
What is the neonatal mortality rate?	Data offices	2	7	14	21	14	29	22	23	52	80
Calculate the number of newborns who died.	Data offices	1	7	9	21	13	29	22	23	45	80
Calculate the KMC coverage – the percentage of newborns receiving KMC among those eligible (target group) in the facility catchment area	Facilities	3	13	32	77	7	52	36	91	78	233
What is the neonatal mortality rate among male babies?	Facilities	2	13	13	77	3	52	36	91	54	233
What is the neonatal mortality rate among female babies?	Facilities	2	13	14	77	3	52	36	91	55	233
What is the neonatal mortality rate?	Facilities	3	13	11	77	4	52	12	91	30	233
calculate the number of newborns who died	Facilities	2	13	11	77	4	52	17	91	34	233
Develop a bar chart depicting the distribution across the maternal ages, of newborns with a low birthweight at the four facilities in Coast District.	Data offices	2	7	8	21	13	29	22	23	45	80
Develop a line graph depicting the trend over one year of KMC coverage among eligible babies born at Bwari Health Center.	Facilities	2	13	25	77	6	52	39	91	72	233
Develop a trend graph (a line graph) depicting the coverage of KMC, by year.	Facilities	4	13	21	77	7	52	18	91	50	233
Interpret the graph above:	Data offices	5	14	32	42	31	58	38	46	106	160
Among the districts shown in the above graph, which attained the target coverage rate (80%) by the end of 2021?	Data offices	3	14	18	42	16	58	22	46	59	160

What guidance could you provide to districts and programs based on these data?	Data offices	2	14	13	42	16	58	22	46	53	160
What do the data above tell you about the neonatal deaths among different birth weight groups in the Kateria City hospital?	Facilities	2	26	28	154	12	104	62	182	104	466
Calculate the neonatal mortality rate in Kateria City hospital during January to March 2021	Facilities	1	26	17	154	7	104	39	182	64	466
For Kateria City hospital to lower their neonatal mortality rate, which birthweight category should they prioritize their focus on?	Facilities	2	26	9	154	8	104	33	182	52	466
Interpret the graph above:	Facilities	6	26	22	154	17	104	31	182	76	466
What aspects of the graph stand out? Is there a trend, or an irregularity? If yes or no, explain the reasons for your answer	Facilities	3	26	11	154	9	104	14	182	37	466
Describe how Dr. Akram and his team defined the data quality problem in this scenario	Data offices	5	14	21	42	28	58	45	46	99	160
	Facilities	11	26	61	154	42	104	130	182	244	466
List potential reasons for the data quality problem encountered:	Data offices	7	21	41	63	53	87	60	69	161	240
	Facilities	20	39	108	231	87	156	176	273	391	699
Describe what major activities/actions Dr. Akram and his team may have included in the district action plan to improve data quality:	Data offices	7	35	57	105	66	145	95	115	225	400
	Facilities	29	65	144	385	94	260	246	455	513	1165
Provide at least one use of the chart findings at the facility level	Data offices	2	7	16	21	24	29	22	23	64	80
Provide at least one use of the chart findings at the community level	Data offices	2	7	12	21	23	29	19	23	56	80
Provide at least one use of the chart findings at the district level	Data offices	3	7	14	21	22	29	22	23	61	80
Provide at least one use of the graph findings at the facility level	Facilities	2	13	26	77	12	52	36	91	76	233
Provide at least one use of the graph findings at the community level	Facilities	1	13	22	77	9	52	24	91	56	233

Provide at least one use of the chart findings at the facility level	Facilities	4	13	9	77	10	52	17	91	40	233
Provide at least one use of the chart findings at the community level	Facilities	4	13	8	77	10	52	12	91	34	233

PRISM index	Facility type	Central African Republic	Ethiopia	Tanzania	Uganda	Overall
		N = 20	N = 98	N = 81	N = 114	N = 313
Calculate percentage pregnant mothers	Data offices	42.9	90.5	51.7	95.7	73.8
Calculate neonatal mortality rate	Data offices	28.6	66.7	48.3	95.7	65.0
Calculate the number of newborns who died	Data offices	14.3	42.9	44.8	95.7	56.3
Calculate KMC coverage	Facilities	23.1	41.6	13.5	39.6	33.5
Calculate neonatal mortality rate among males	Facilities	15.4	16.9	5.8	39.6	23.2
Calculate neonatal mortality rate among females	Facilities	15.4	18.2	5.8	39.6	23.6
Calculate neonatal mortality rate	Facilities	23.1	14.3	7.7	13.2	12.9
Calculate the number of newborns who died	Facilities	15.4	14.3	7.7	18.7	14.6
Develop a bar chart	Data offices	28.6	38.1	44.8	95.7	56.3
Develop a line chart	Facilities	15.4	32.5	11.5	42.9	30.9
Develop a trend graph	Facilities	30.8	27.3	13.5	19.8	21.5
Interpret the graph	Data offices	35.7	76.2	53.4	82.6	66.3
Extract info from graph	Data offices	21.4	42.9	27.6	47.8	36.9
Provide guidance from interpretation	Data offices	14.3	31.0	27.6	47.8	33.1
Interpret data	Facilities	7.7	18.2	11.5	34.1	22.3
Extract neonatal mortality rate	Facilities	3.8	11.0	6.7	21.4	13.7
Indicate priority for care improvement	Facilities	7.7	5.8	7.7	18.1	11.2
Interpret the graph	Facilities	23.1	14.3	16.3	17.0	16.3
Extract info from graph	Facilities	11.5	7.1	8.7	7.7	7.9
Describe scenario	Data offices	35.7	50.0	48.3	97.8	61.9
Describe scenario	Facilities	42.3	39.6	40.4	71.4	52.4
List problematic data quality aspects	Data offices	33.3	65.1	60.9	87.0	67.1
List problematic data quality aspects	Facilities	51.3	46.8	55.8	64.5	55.9
Describe plan for quality improvement	Data offices	20.0	54.3	45.5	82.6	56.3
Describe plan for quality improvement	Facilities	44.6	37.4	36.2	54.1	44.0
Provide chart finding at facility level	Data offices	28.6	76.2	82.8	95.7	80.0
Provide chart finding at community level	Data offices	28.6	57.1	79.3	82.6	70.0
Provide chart finding at district level	Data offices	42.9	66.7	75.9	95.7	76.3
Provide chart finding at facility level (1)	Facilities	15.4	33.8	23.1	39.6	32.6
Provide chart finding at community level (1)	Facilities	7.7	28.6	17.3	26.4	24.0
Provide chart finding at facility level (2)	Facilities	30.8	11.7	19.2	18.7	17.2
Provide chart finding at community level (2)	Facilities	30.8	10.4	19.2	13.2	14.6
**Number of key indicators >80**		0	1	1	11	0
**Percentage of key indicators >80**		0	3.1	3.1	34.4	0

ANC – antenatal care, eRHIS – electronic routine health information system, KMC – kangaroo mother care, PRISM - performance of routine information system management

Table S13. Users' perspective regarding the eRHIS

Users' perspective	Facility type	Outcome	Central African Republic		Ethiopia		Tanzania		Uganda		Overall	
			N	%	N	%	N	%	N	%	N	%
			N = 21		N = 35		N = 46		N = 49		N = 151	
Rating given by respondent	Data offices	Easy to use	3	42.9	8	72.7	8	44.4	15	75	34	60.7
	Data offices	Moderate	3	42.9	3	27.2	10	55.6	5	25	21	37.5
	Data offices	Difficult to use	0	0	0	0	0	0.0	0	0	0	0.0
	Data offices	Not assessed	1	14.2	0	0	0	0.0	0	0	1	1.8
	Facilities	Easy to use	4	28.5	11	45.8	9	32.1	14	48.2	38	40.0
	Facilities	Moderate	0	0	9	37.5	11	39.3	14	48.2	34	35.8
	Facilities	Difficult to use	0	0	3	12.5	7	25.0	0	0	10	10.5
	Facilities	Not assessed	10	71.5	1	4.2	1	3.6	1	3.6	13	13.7
Opinion by respondent (need for improvement)	Data offices	Yes	5	71.4	8	72.7	17	94.4	10	50	40	71.4
	Data offices	No	1	14.3	2	18.1	1	5.6	10	50	14	25.0
	Data offices	Not assessed	1	14.3	1	9.1	0	0.0	0	0	2	3.6
	Facilities	Yes	4	28.6	22	91.6	17	60.7	15	51.7	58	61.1
	Facilities	No	0	0	0	0	10	35.7	13	44.8	23	24.2
	Facilities	Not assessed	10	71.4	2	8.4	1	3.6	1	3.5	14	14.7

PRISM index	Facility type	Outcome	Central African Republic	Ethiopia	Tanzania	Uganda	Overall
			N = 21	N = 35	N = 46	N = 49	N = 151
Rating given by respondent	Data offices	Easy to use	42.9	72.7	44.4	75	60.7
	Data offices	Moderate	42.9	27.2	55.6	25	37.5
	Data offices	Difficult to use	0	0	0.0	0	0.0
	Facilities	Easy to use	28.5	45.8	32.1	48.2	40.0
	Facilities	Moderate	0	37.5	39.3	48.2	35.8
	Facilities	Difficult to use	0	12.5	25.0	0	10.5
Opinion by respondent (need for improvement)	Data offices	Yes	71.4	72.7	94.4	50	71.4
	Data offices	No	14.3	18.1	5.6	50	25.0
	Facilities	Yes	28.6	91.6	60.7	51.7	61.1
	Facilities	No	0	0	35.7	44.8	24.2
**Number of key indicators >80**			0	1	1	0	0
**Percentage of key indicators >80**			0	10	10	0	0

In yellow are marked the indicators that contributed to the calculation via average of the PRISM indexes

eRHIS – electronic routine health information system, PRISM - performance of routine information system management

Table S14. Reporting capabilities by facility type

**Track report completeness using eRHIS**	**Facility type**	Central African Republic						Ethiopia						Tanzania						Uganda					
		Yes		No		NA		Yes		No		NA		Yes		No		NA		Yes		No		NA	
		N	%	N	%	N	%	N	%	N	%	N	%	N	%	N	%	N	%	N	%	N	%	N	%
		N = 21						N = 35						N = 46						N = 49					
	Third level of referral	1	25	0	0.0	3	75.0	2	66.7	0	0.0	1	33.3	5	100	0	0.0	0	0.0	4	80.0	0	0.0	1	20.0
	Second level of referral	0	0.0	0	0.0	3	100	6	60.0	2	20.0	2	20.0	11	78.6	2	14.3	1	7.1	12	100	0	0.0	0	0.0
	First level of referral	0	0.0	0	0.0	7	100	2	18.2	5	45.5	4	36.4	8	88.9	1	11.1	0	0.0	10	83.3	1	8.3	1	8.3
	District/subnational health data office	5	83.3	1	16.7	0	0.0	6	100	0	0.0	0	0.0	13	92.9	1	7.1	0	0.0	18	90.0	2	10.0	0	0.0
	Regional/provincial health office	1	100	0	0.0	0	0.0	5	100	0	0.0	0	0.0	4	100	0	0.0	0	0.0						

**Capacity to generate summary reports**	**Facility type**	Central African Republic						Ethiopia						Tanzania						Uganda					
		Yes		No		NA		Yes		No		NA		Yes		No		NA		Yes		No		NA	
		N	%	N	%	N	%	N	%	N	%	N	%	N	%	N	%	N	%	N	%	N	%	N	%
		N = 21						N = 35						N = 46						N = 49					
National/regional summary for a year	Third level of referral	0	0.0	1	25.0	3	75.0	1	33.3	0	0.0	2	66.7	5	100	0	0.0	0	0.0	4	80.0	1	20.0	0	0.0
	Second level of referral	0	0.0	0	0.0	3	100	2	20.0	0	0.0	8	80.0	10	71.4	3	21.4	1	7.1	2	16.7	9	75.0	1	8.3
	First level of referral	0	0.0	0	0.0	7	100	0	0.0	0	0.0	11	100	6	66.7	3	33.3	0	0.0	5	41.7	6	50.0	1	8.3
	District/subnational health data office	0	0.0	4	66.7	2	33.3	2	33.3	3	50.0	1	16.7	12	85.7	1	7.1	1	7.1	12	60.0	7	35.0	1	5.0
	Regional/provincial health office	1	100	0	0.0	0	0.0	5	100	0	0.0	0	0.0	4	100	0	0.0	0	0.0						
District summary for a year	Third level of referral	0	0.0	1	25.0	3	75.0	1	33.3	0	0.0	2	66.7	5	100	0	0.0	0	0.0	4	80.0	0	0.0	1	20.0
	Second level of referral	0	0.0	0	0.0	3	100	4	36.4	0	0.0	7	63.6	10	71.4	3	21.4	1	7.1	10	83.3	1	8.3	1	8.3
	First level of referral	0	0.0	0	0.0	7	100	1	10.0	1	10.0	8	80.0	6	66.7	3	33.3	0	0.0	8	66.7	3	25.0	1	8.3
	District/subnational health data office	1	16.7	5	83.3	0	0.0	6	100	0	0.0	0	0.0	14	100	0	0.0	0	0.0	20	100	0	0.0	0	0.0
	Regional/provincial health office	1	100	0	0.0	0	0.0	4	80.0	1	20.0	0	0.0	4	100	0	0.0	0	0.0						
Health facility summary for a year	Third level of referral	4	100.0	0	0.0	0	0.0	3	100	0	0.0	0	0.0	5	100	0	0.0	0	0.0	5	100	0	0.0	0	0.0
	Second level of referral	0	0.0	0	0.0	3	100	9	90.0	1	10.0	0	0.0	13	92.9	0	0.0	1	7.1	12	100	0	0.0	0	0.0
	First level of referral	0	0.0	0	0.0	7	100	10	90.9	0	0.0	1	9.1	8	88.9	1	11.1	0	0.0	11	91.7	0	0.0	1	8.3

	District/subnational health data office	6	100	0	0.0	0	0.0	5	83.3	1	16.7	0	0.0	14	100	0	0.0	0	0.0	20	100	0	0.0	0	0.0
	Regional/provincial health office	1	100	0	0.0	0	0.0	5	100	0	0.0	0	0.0	4	100	0	0.0	0	0.0						
Community-level SDP summary for a year	Third level of referral	1	25.0	0	0.0	3	75.0	0	0.0	1	33.3	2	66.7	1	20.0	2	40.0	2	40.0	2	40.0	3	60.0	0	0.0
	Second level of referral	0	0.0	0	0.0	3	100	1	10.0	3	30.0	6	60.0	1	7.1	5	35.7	8	57.1	6	50.0	5	41.7	1	8.3
	First level of referral	0	0.0	0	0.0	7	100	4	36.4	3	27.3	4	36.4	1	11.1	3	33.3	5	55.6	4	33.3	7	58.3	1	8.3
	District/subnational health data office	0	0.0	6	100	0	0.0	5	83.3	1	16.7	0	0.0	1	7.1	4	28.6	9	64.3	12	60.0	7	35.0	1	5.0
	Regional/provincial health office	0	0.0	1	100	0	0.0	3	60.0	0	0.0	2	40.0	0	0.0	2	50.0	2	50.0						

**PRISM Index**	**Facility type**	Central African Republic	Ethiopia	Tanzania	Uganda
		N = 21	N = 35	N = 46	N = 49
Track report completeness using eRHIS	Third level of referral	25.0	66.7	100.0	80.0
	Second level of referral	0.0	60.0	78.6	100.0
	First level of referral	0.0	18.2	88.9	83.3
	District/subnational health data office	83.3	100.0	92.9	90.0
	Regional/provincial health office	100.0	100.0	100.0	
Capacity to generate summary reports National/regional summary for a year	Third level of referral	0.0	33.3	100.0	80.0
	Second level of referral	0.0	20.0	71.4	16.7
	First level of referral	0.0	0.0	66.7	41.7
	District/subnational health data office	0.0	33.3	85.7	60.0
	Regional/provincial health office	100.0	100.0	100.0	
District summary for a year	Third level of referral	0.0	33.3	100.0	80.0
	Second level of referral	0.0	36.4	71.4	83.3
	First level of referral	0.0	10.0	66.7	66.7
	District/subnational health data office	16.7	100.0	100.0	100.0
	Regional/provincial health office	100.0	80.0	100.0	

Health facility summary for a year	Third level of referral	100.0	100.0	100.0	100.0
	Second level of referral	0.0	90.0	92.9	100.0
	First level of referral	0.0	90.9	88.9	91.7
	District/subnational health data office	100.0	83.3	100.0	100.0
	Regional/provincial health office	100.0	100.0	100.0	
Community-level SDP summary for a year - Facility type	Third level of referral	25.0	0.0	20.0	40.0
	Second level of referral	0.0	10.0	7.1	50.0
	First level of referral	0.0	36.4	11.1	33.3
	District/subnational health data office	0.0	83.3	7.1	60.0
	Regional/provincial health office	0.0	60.0	0.0	

In yellow are marked the indicators that contributed to the calculation via average of the PRISM indexes

eRHIS – electronic routine health information system, NA – not answered, PRISM - performance of routine information system management, SDP – self-determination program

Table S15. Calculation capabilities by facility type

Ability to calculate coverage indicators	Facility type	Central African Republic						Ethiopia						Tanzania						Uganda					
		Yes		No		NA		Yes		No		NA		Yes		No		NA		Yes		No		NA	
		N	%	N	%	N	%	N	%	N	%	N	%	N	%	N	%	N	%	N	%	N	%	N	%
		N = 21						N = 35						N = 46						N = 49					
**Neonatal mortality rate - National**	Third level of referral	0	0.0	1	25.0	3	75.0	1	33.3	0	0.0	2	66.7	2	40.0	3	60.0	0	0.0	1	20.0	2	40.0	2	40.0
	Second level of referral	0	0.0	0	0.0	3	100.0	2	20.0	1	10.0	7	70.0	3	21.4	10	71.4	1	7.1	0	0.0	11	91.7	1	8.3
	First level of referral	0	0.0	0	0.0	7	100.0	0	0.0	0	0.0	11	100.0	1	11.1	8	88.9	0	0.0	4	33.3	7	58.3	1	8.3
	District/subnational health data office	0	0.0	4	66.7	2	33.3	0	0.0	3	50.0	3	50.0	3	21.4	9	64.3	2	14.3	9	45.0	9	45.0	2	10.0
	Regional / provincial health office	1	100.0	0	0.0	0	0.0	4	80.0	1	20.0	0	0.0	3	75.0	1	25.0	0	0.0						
**Neonatal mortality rate - Region**	Third level of referral	0	0.0	1	25.0	3	75.0	1	33.3	0	0.0	2	66.7	2	40.0	3	60.0	0	0.0	4	80.0	0	0.0	1	20.0
	Second level of referral	0	0.0	0	0.0	3	100.0	2	20.0	1	10.0	7	70.0	3	21.4	10	71.4	1	7.1	1	8.3	10	83.3	1	8.3
	First level of referral	0	0.0	0	0.0	7	100.0	0	0.0	0	0.0	11	100.0	1	11.1	8	88.9	0	0.0	4	33.3	7	58.3	1	8.3
	District/subnational health data office	0	0.0	4	66.7	2	33.3	2	33.3	3	50.0	1	16.7	3	21.4	9	64.3	2	14.3	13	65.0	5	25.0	2	10.0
	Regional / provincial health office	1	100.0	0	0.0	0	0.0	5	100.0	0	0.0	0	0.0	3	75.0	1	25.0	0	0.0						
**Neonatal mortality rate - District**	Third level of referral	0	0.0	1	25.0	3	75.0	1	33.3	0	0.0	2	66.7	2	40.0	3	60.0	0	0.0	4	80.0	0	0.0	1	20.0
	Second level of referral	0	0.0	0	0.0	3	100.0	3	30.0	2	20.0	5	50.0	3	21.4	10	71.4	1	7.1	8	66.7	3	25.0	1	8.3
	First level of referral	0	0.0	0	0.0	7	100.0	0	0.0	1	9.1	10	90.9	1	11.1	8	88.9	0	0.0	7	58.3	4	33.3	1	8.3
	District/subnational health data office	2	33.3	4	66.7	0	0.0	5	83.3	1	16.7	0	0.0	7	50.0	7	50.0	0	0.0	20	100.0	0	0.0	0	0.0
	Regional / provincial health office	1	100.0	0	0.0	0	0.0	5	100.0	0	0.0	0	0.0	3	75.0	1	25.0	0	0.0						
**Neonatal mortality rate - Health facility**	Third level of referral	4	100.0	0	0.0	0	0.0	3	100.0	0	0.0	0	0.0	3	60.0	2	40.0	0	0.0	5	100.0	0	0.0	0	0.0
	Second level of referral	0	0.0	0	0.0	3	100.0	6	60.0	4	40.0	0	0.0	6	42.9	7	50.0	1	7.1	11	91.7	1	8.3	0	0.0

	First level of referral	0	0.0	0	0.0	7	100.0	5	45.5	5	45.5	1	9.1	2	22.2	7	77.8	0	0.0	9	75.0	2	16.7	1	8.3
	District/subnational health data office	5	83.3	1	16.7	0	0.0	4	66.7	2	33.3	0	0.0	7	50.0	7	50.0	0	0.0	20	100.0	0	0.0	0	0.0
	Regional / provincial health office	1	100.0	0	0.0	0	0.0	5	100.0	0	0.0	0	0.0	3	75.0	1	25.0	0	0.0						
**Neonatal mortality rate - Community-level SDP**	Third level of referral	0	0.0	1	25.0	3	75.0	0	0.0	1	33.3	2	66.7	0	0.0	3	60.0	2	40.0	1	20.0	3	60.0	1	20.0
	Second level of referral	0	0.0	0	0.0	3	100.0	1	10.0	3	30.0	6	60.0	0	0.0	5	35.7	9	64.3	6	50.0	5	41.7	1	8.3
	First level of referral	0	0.0	0	0.0	7	100.0	0	0.0	5	45.5	6	54.5	0	0.0	4	44.4	5	55.6	4	33.3	7	58.3	1	8.3
	District/subnational health data office	0	0.0	5	83.3	1	16.7	1	16.7	3	50.0	2	33.3	0	0.0	5	35.7	9	64.3	6	30.0	13	65.0	1	5.0
	Regional / provincial health office	0	0.0	1	100.0	0	0.0	4	80.0	0	0.0	1	20.0	0	0.0	1	25.0	3	75.0						
**Stillbirth rate - National**	Third level of referral	0	0.0	1	25.0	3	75.0	1	33.3	0	0.0	2	66.7	2	40.0	3	60.0	0	0.0	1	20.0	2	40.0	2	40.0
	Second level of referral	0	0.0	0	0.0	3	100.0	2	20.0	1	10.0	7	70.0	3	21.4	10	71.4	1	7.1	0	0.0	11	91.7	1	8.3
	First level of referral	0	0.0	0	0.0	7	100.0	0	0.0	0	0.0	11	100.0	1	11.1	8	88.9	0	0.0	4	33.3	7	58.3	1	8.3
	District/subnational health data office	0	0.0	4	66.7	2	33.3	0	0.0	3	50.0	3	50.0	4	28.6	8	57.1	2	14.3	9	45.0	8	40.0	3	15.0
	Regional / provincial health office	1	100.0	0	0.0	0	0.0	4	80.0	1	20.0	0	0.0	3	75.0	1	25.0	0	0.0						
**Stillbirth rate - Region**	Third level of referral	0	0.0	1	25.0	3	75.0	1	33.3	0	0.0	2	66.7	2	40.0	3	60.0	0	0.0	4	80.0	0	0.0	1	20.0
	Second level of referral	0	0.0	0	0.0	3	100.0	2	20.0	1	10.0	7	70.0	3	21.4	10	71.4	1	7.1	1	8.3	10	83.3	1	8.3
	First level of referral	0	0.0	0	0.0	7	100.0	0	0.0	0	0.0	11	100.0	1	11.1	8	88.9	0	0.0	4	33.3	7	58.3	1	8.3
	District/subnational health data office	0	0.0	4	66.7	2	33.3	2	33.3	3	50.0	1	16.7	4	28.6	8	57.1	2	14.3	12	60.0	6	30.0	2	10.0
	Regional / provincial health office	1	100.0	0	0.0	0	0.0	5	100.0	0	0.0	0	0.0	3	75.0	1	25.0	0	0.0						
**Stillbirth rate - District**	Third level of referral	0	0.0	1	25.0	3	75.0	1	33.3	0	0.0	2	66.7	2	40.0	3	60.0	0	0.0	4	80.0	0	0.0	1	20.0
	Second level of referral	0	0.0	0	0.0	3	100.0	3	30.0	2	20.0	5	50.0	3	21.4	10	71.4	1	7.1	8	66.7	3	25.0	1	8.3
	First level of referral	0	0.0	0	0.0	7	100.0	0	0.0	1	9.1	10	90.9	1	11.1	8	88.9	0	0.0	7	58.3	4	33.3	1	8.3
	District/subnational health data office	2	33.3	4	66.7	0	0.0	5	83.3	1	16.7	0	0.0	8	57.1	6	42.9	0	0.0	20	100.0	0	0.0	0	0.0

	Regional / provincial health office	1	100.0	0	0.0	0	0.0	5	100.0	0	0.0	0	0.0	3	75.0	1	25.0	0	0.0						
**Stillbirth rate - Health facility**	Third level of referral	3	75.0	0	0.0	1	25.0	3	100.0	0	0.0	0	0.0	3	60.0	2	40.0	0	0.0	5	100.0	0	0.0	0	0.0
	Second level of referral	0	0.0	0	0.0	3	100.0	7	70.0	3	30.0	0	0.0	6	42.9	7	50.0	1	7.1	11	91.7	1	8.3	0	0.0
	First level of referral	0	0.0	0	0.0	7	100.0	5	45.5	5	45.5	1	9.1	3	33.3	6	66.7	0	0.0	9	75.0	2	16.7	1	8.3
	District/subnational health data office	5	83.3	1	16.7	0	0.0	4	66.7	2	33.3	0	0.0	8	57.1	6	42.9	0	0.0	20	100.0	0	0.0	0	0.0
	Regional / provincial health office	1	100.0	0	0.0	0	0.0	5	100.0	0	0.0	0	0.0	3	75.0	1	25.0	0	0.0						
**Stillbirth rate - Community-level SDP**	Third level of referral	0	0.0	1	25.0	3	75.0	0	0.0	1	33.3	2	66.7	0	0.0	3	60.0	2	40.0	2	40.0	2	40.0	1	20.0
	Second level of referral	0	0.0	0	0.0	3	100.0	1	10.0	3	30.0	6	60.0	0	0.0	5	35.7	9	64.3	5	41.7	5	41.7	2	16.7
	First level of referral	0	0.0	0	0.0	7	100.0	0	0.0	5	45.5	6	54.5	0	0.0	4	44.4	5	55.6	4	33.3	7	58.3	1	8.3
	District/subnational health data office	0	0.0	5	83.3	1	16.7	1	16.7	3	50.0	2	33.3	0	0.0	5	35.7	9	64.3	6	30.0	13	65.0	1	5.0
	Regional / provincial health office	0	0.0	1	100.0	0	0.0	4	80.0	0	0.0	1	20.0	0	0.0	1	25.0	3	75.0						
**Low birth weight rate - National**	Third level of referral	0	0.0	1	25.0	3	75.0	1	33.3	0	0.0	2	66.7	2	40.0	3	60.0	0	0.0	1	20.0	2	40.0	2	40.0
	Second level of referral	0	0.0	0	0.0	3	100.0	2	20.0	1	10.0	7	70.0	3	21.4	10	71.4	1	7.1	0	0.0	11	91.7	1	8.3
	First level of referral	0	0.0	0	0.0	7	100.0	0	0.0	0	0.0	11	100.0	1	11.1	8	88.9	0	0.0	4	33.3	7	58.3	1	8.3
	District/subnational health data office	0	0.0	4	66.7	2	33.3	0	0.0	3	50.0	3	50.0	4	28.6	8	57.1	2	14.3	9	45.0	8	40.0	3	15.0
	Regional / provincial health office	1	100.0	0	0.0	0	0.0	3	60.0	2	40.0	0	0.0	3	75.0	1	25.0	0	0.0						
**Low birth weight rate - Region**	Third level of referral	0	0.0	1	25.0	3	75.0	1	33.3	0	0.0	2	66.7	2	40.0	3	60.0	0	0.0	4	80.0	0	0.0	1	20.0
	Second level of referral	0	0.0	0	0.0	3	100.0	2	20.0	1	10.0	7	70.0	3	21.4	10	71.4	1	7.1	1	8.3	10	83.3	1	8.3
	First level of referral	0	0.0	0	0.0	7	100.0	0	0.0	0	0.0	11	100.0	1	11.1	8	88.9	0	0.0	4	33.3	7	58.3	1	8.3
	District/subnational health data office	0	0.0	4	66.7	2	33.3	2	33.3	3	50.0	1	16.7	4	28.6	8	57.1	2	14.3	12	60.0	6	30.0	2	10.0
	Regional / provincial health office	1	100.0	0	0.0	0	0.0	4	80.0	1	20.0	0	0.0	3	75.0	1	25.0	0	0.0						
**Low birth weight rate- District**	Third level of referral	0	0.0	1	25.0	3	75.0	1	33.3	0	0.0	2	66.7	2	40.0	3	60.0	0	0.0	4	80.0		0.0	1	20.0

	Second level of referral	0	0.0	0	0.0	3	100.0	3	30.0	2	20.0	5	50.0	3	21.4	10	71.4	1	7.1	8	66.7	3	25.0	1	8.3
	First level of referral	0	0.0	0	0.0	7	100.0	0	0.0	1	9.1	10	90.9	1	11.1	8	88.9	0	0.0	7	58.3	4	33.3	1	8.3
	District/subnational health data office	2	33.3	4	66.7	0	0.0	5	83.3	1	16.7	0	0.0	8	57.1	6	42.9	0	0.0	20	100.0		0.0		0.0
	Regional / provincial health office	1	100.0	0	0.0	0	0.0	4	80.0	1	20.0	0	0.0	3	75.0	1	25.0	0	0.0						
**Low birth weight rate - Health facility**	Third level of referral	4	100.0	0	0.0	0	0.0	2	66.7	1	33.3	0	0.0	3	60.0	2	40.0	0	0.0	5	31.3	11	68.8	0	0.0
	Second level of referral	0	0.0	0	0.0	3	100.0	7	70.0	3	30.0	0	0.0	6	42.9	7	50.0	1	7.1	0	0.0	1	100.0	0	0.0
	First level of referral	0	0.0	0	0.0	7	100.0	4	36.4	6	54.5	1	9.1	3	33.3	6	66.7	0	0.0	8	66.7	3	25.0	1	8.3
	District/subnational health data office	5	83.3	1	16.7	0	0.0	4	66.7	2	33.3	0	0.0	8	57.1	6	42.9	0	0.0	20	100.0	0	0.0	0	0.0
	Regional / provincial health office	1	100.0	0	0.0	0	0.0	4	80.0	1	20.0	0	0.0	3	75.0	1	25.0	0	0.0						
**Low birth weight rate - Community-level SDP**	Third level of referral	0	0.0	1	25.0	3	75.0	0	0.0	1	33.3	2	66.7	0	0.0	3	60.0	2	40.0	1	20.0	3	60.0	1	20.0
	Second level of referral	0	0.0	0	0.0	3	100.0	1	10.0	3	30.0	6	60.0	0	0.0	5	35.7	9	64.3	5	41.7	5	41.7	2	16.7
	First level of referral	0	0.0	0	0.0	7	100.0	0	0.0	5	45.5	6	54.5	0	0.0	4	44.4	5	55.6	4	33.3	7	58.3	1	8.3
	District/subnational health data office	0	0.0	5	83.3	1	16.7	1	16.7	3	50.0	2	33.3	0	0.0	5	35.7	9	64.3	6	30.0	13	65.0	1	5.0
	Regional / provincial health office	0	0.0	1	100.0	0	0.0	3	60.0	1	20.0	1	20.0	0	0.0	1	25.0	3	75.0						

**PRISM index**	**Facility type**	Central African Republic	Ethiopia	Tanzania	Uganda
		N = 21	N = 35	N = 46	N = 49
**Neonatal mortality rate - National**	Third level of referral	0.0	33.3	40.0	20.0
	Second level of referral	0.0	20.0	21.4	0.0
	First level of referral	0.0	0.0	11.1	33.3
	District/subnational health data office	0.0	0.0	21.4	45.0
	Regional/provincial health office	100.0	80.0	75.0	

**Neonatal mortality rate - Region**	Third level of referral	0.0	33.3	40.0	80.0
	Second level of referral	0.0	20.0	21.4	8.3
	First level of referral	0.0	0.0	11.1	33.3
	District/subnational health data office	0.0	33.3	21.4	65.0
	Regional/provincial health office	100.0	100.0	75.0	
**Neonatal mortality rate - District**	Third level of referral	0.0	33.3	40.0	80.0
	Second level of referral	0.0	30.0	21.4	66.7
	First level of referral	0.0	0.0	11.1	58.3
	District/subnational health data office	33.3	83.3	50.0	100.0
	Regional/provincial health office	100.0	100.0	75.0	
**Neonatal mortality rate - Health facility**	Third level of referral	100.0	100.0	60.0	100.0
	Second level of referral	0.0	60.0	42.9	91.7
	First level of referral	0.0	45.5	22.2	75.0
	District/subnational health data office	83.3	66.7	50.0	100.0
	Regional/provincial health office	100.0	100.0	75.0	
**Neonatal mortality rate - Community-level SDP**	Third level of referral	0.0	0.0	0.0	20.0
	Second level of referral	0.0	10.0	0.0	50.0
	First level of referral	0.0	0.0	0.0	33.3
	District/subnational health data office	0.0	16.7	0.0	30.0
	Regional/provincial health office	0.0	80.0	0.0	
**Stillbirth rate - National**	Third level of referral	0.0	33.3	40.0	20.0
	Second level of referral	0.0	20.0	21.4	0.0

	First level of referral	0.0	0.0	11.1	33.3
	District/subnational health data office	0.0	0.0	28.6	45.0
	Regional/provincial health office	100.0	80.0	75.0	
**Stillbirth rate - Region**	Third level of referral	0.0	33.3	40.0	80.0
	Second level of referral	0.0	20.0	21.4	8.3
	First level of referral	0.0	0.0	11.1	33.3
	District/subnational health data office	0.0	33.3	28.6	60.0
	Regional/provincial health office	100.0	100.0	75.0	
**Stillbirth rate - District**	Third level of referral	0.0	33.3	40.0	80.0
	Second level of referral	0.0	30.0	21.4	66.7
	First level of referral	0.0	0.0	11.1	58.3
	District/subnational health data office	33.3	83.3	57.1	100.0
	Regional/provincial health office	100.0	100.0	75.0	
**Stillbirth rate - Health facility**	Third level of referral	75.0	100.0	60.0	100.0
	Second level of referral	0.0	70.0	42.9	91.7
	First level of referral	0.0	45.5	33.3	75.0
	District/subnational health data office	83.3	66.7	57.1	100.0
	Regional/provincial health office	100.0	100.0	75.0	
**Stillbirth rate - Community-level SDP**	Third level of referral	0.0	0.0	0.0	40.0
	Second level of referral	0.0	10.0	0.0	41.7
	First level of referral	0.0	0.0	0.0	33.3
	District/subnational health data office	0.0	16.7	0.0	30.0
	Regional/provincial health office	0.0	80.0	0.0	
**Low birth weight rate - National**	Third level of referral	0.0	33.3	40.0	20.0

	Second level of referral	0.0	20.0	21.4	0.0
	First level of referral	0.0	0.0	11.1	33.3
	District/subnational health data office	0.0	0.0	28.6	45.0
	Regional/provincial health office	100.0	60.0	75.0	
**Low birth weight rate - Region**	Third level of referral	0.0	33.3	40.0	80.0
	Second level of referral	0.0	20.0	21.4	8.3
	First level of referral	0.0	0.0	11.1	33.3
	District/subnational health data office	0.0	33.3	28.6	60.0
	Regional/provincial health office	100.0	80.0	75.0	
**Low birth weight rate- District**	Third level of referral	0.0	33.3	40.0	80.0
	Second level of referral	0.0	30.0	21.4	66.7
	First level of referral	0.0	0.0	11.1	58.3
	District/subnational health data office	33.3	83.3	57.1	100.0
	Regional/provincial health office	100.0	80.0	75.0	
**Low birth weight rate - Health facility**	Third level of referral	100.0	66.7	60.0	31.3
	Second level of referral	0.0	70.0	42.9	0.0
	First level of referral	0.0	36.4	33.3	66.7
	District/subnational health data office	83.3	66.7	57.1	100.0
	Regional/provincial health office	100.0	80.0	75.0	
**Low birth weight rate - Community-level SDP**	Third level of referral	0.0	0.0	0.0	20.0
	Second level of referral	0.0	10.0	0.0	41.7
	First level of referral	0.0	0.0	0.0	33.3
	District/subnational health data office	0.0	16.7	0.0	30.0

	Regional/provincial health office	0.0	60.0	0.0	

Aggregated PRISM index	Facility type	Central African Republic	Ethiopia	Tanzania	Uganda
		N = 21	N = 35	N = 46	N = 49
**Neonatal mortality rate**	Third level of referral	20.0	40.0	36.0	60.0
	Second level of referral	0.0	28.0	21.4	43.3
	First level of referral	0.0	9.1	11.1	46.7
	District/subnational health data office	23.3	40.0	28.6	68.0
	Regional/provincial health office	80.0	92.0	60.0	
**Stillbirth rate**	Third level of referral	15.0	40.0	36.0	64.0
	Second level of referral	0.0	30.0	21.4	41.7
	First level of referral	0.0	9.1	13.3	46.7
	District/subnational health data office	23.3	40.0	34.3	67.0
	Regional/provincial health office	80.0	92.0	60.0	
**Low birth weight rate**	Third level of referral	20.0	33.3	36.0	46.3
	Second level of referral	0.0	30.0	21.4	23.3
	First level of referral	0.0	7.3	13.3	45.0
	District/subnational health data office	23.3	40.0	34.3	67.0
	Regional/provincial health office	80.0	72.0	60.0	

In yellow are marked the indicators that contributed to the calculation via average of the PRISM indexes

NA – not answered, PRISM - performance of routine information system management, SDP – self-determination program

Table S16. Data analysis capabilities by facility type

Percentage of staff demonstrating the use of data analysis features	Facility type	Central African Republic						Ethiopia						Tanzania						Uganda					
		Yes		No		NA		Yes		No		NA		Yes		No		NA		Yes		No		NA	
		N	%	N	%	N	%	N	%	N	%	N	%	N	%	N	%	N	%	N	%	N	%	N	%
		N = 21						N = 35						N = 46						N = 49					
**Major causes neonatal mortality**	Third level of referral	1	25.0	3	75.0	0	0.0	1	33.3	2	66.7	0	0.0	4	80.0	1	20.0	0	0.0	4	80.0	1	20.0	0	0.0
	Second level of referral	0	0.0	0	0.0	3	100.0	3	30.0	6	60.0	1	10.0	1	7.1	12	85.7	1	7.1	10	83.3	2	16.7	0	0.0
	First level of referral	0	0.0	0	0.0	7	100.0	1	9.1	9	81.8	1	9.1	2	22.2	7	77.8	0	0.0	7	58.3	4	33.3	1	8.3
	District/subnational health data office	0	0.0	6	100.0	0	0.0	0	0.0	6	100.0	0	0.0	8	57.1	6	42.9	0	0.0	17	85.0	3	15.0	0	0.0
	Regional / provincial health office	0	0.0	1	100.0	0	0.0	0	0.0	5	100.0	0	0.0	3	75.0	1	25.0	0	0.0						
**Major causes neonatal morbidity**	Third level of referral	0	0.0	3	75.0	1	25.0	1	33.3	2	66.7	0	0.0	4	80.0	1	20.0	0	0.0	4	80.0	1	20.0	0	0.0
	Second level of referral	0	0.0	0	0.0	3	100.0	3	30.0	6	60.0	1	10.0	0	0.0	13	92.9	1	7.1	10	83.3	2	16.7	0	0.0
	First level of referral	0	0.0	0	0.0	7	100.0	2	18.2	8	72.7	1	9.1	2	22.2	7	77.8	0	0.0	5	41.7	6	50.0	1	8.3
	District/subnational health data office	0	0.0	6	100.0	0	0.0	1	16.7	5	83.3	0	0.0	6	42.9	7	50.0	1	7.1	19	95.0	1	5.0	0	0.0
	Regional / provincial health office	0	0.0	1	100.0	0	0.0	1	20.0	4	80.0	0	0.0	3	75.0	1	25.0	0	0.0						
**Data disaggregation**	Third level of referral	2	50.0	1	25.0	1	25.0	1	33.3	2	66.7	0	0.0	3	60.0	2	40.0	0	0.0	5	100.0	0	0.0	0	0.0
	Second level of referral	0	0.0	0	0.0	3	100.0	1	10.0	9	90.0	0	0.0	6	42.9	7	50.0	1	7.1	5	41.7	7	58.3	0	0.0
	First level of referral	0	0.0	0	0.0	7	100.0	0	0.0	9	81.8	2	18.2	1	11.1	8	88.9	0	0.0	4	33.3	7	58.3	1	8.3
	District/subnational health data office	1	100.0	0	0.0	0	0.0	0	0.0	6	100.0	0	0.0	6	42.9	8	57.1	0	0.0	15	75.0	5	25.0	0	0.0
	Regional / provincial health office	1	100.0	0	0.0	0	0.0	0	0.0	5	100.0	0	0.0	2	50.0	2	50.0	0	0.0						

PRISM index	Facility type	Central African Republic	Ethiopia	Tanzania	Uganda
		N = 21	N = 35	N = 46	N = 49

**Major causes neonatal mortality**	Third level of referral	25.0	33.3	80.0	80.0
	Second level of referral	0.0	30.0	7.1	83.3
	First level of referral	0.0	9.1	22.2	58.3
	District/subnational health data office	0.0	0.0	57.1	85.0
	Regional / provincial health office	0.0	0.0	75.0	
**Major causes neonatal morbidity**	Third level of referral	0.0	33.3	80.0	80.0
	Second level of referral	0.0	30.0	0.0	83.3
	First level of referral	0.0	18.2	22.2	41.7
	District/subnational health data office	0.0	16.7	42.9	95.0
	Regional / provincial health office	0.0	20.0	75.0	
**Data disaggregation**	Third level of referral	50.0	33.3	60.0	100.0
	Second level of referral	0.0	10.0	42.9	41.7
	First level of referral	0.0	0.0	11.1	33.3
	District/subnational health data office	100.0	0.0	42.9	75.0
	Regional / provincial health office	100.0	0.0	50.0	

In yellow are marked the indicators that contributed to the calculation via average of the PRISM indexes

NA – not answered

Table S17. Visualization capabilities by facility type

Percentage of staff able to use the data visualization features of the eRHIS to analyse and present data in graphs and maps	Facility type	Central African Republic						Ethiopia						Tanzania						Uganda					
		Yes		No		NA		Yes		No		NA		Yes		No		NA		Yes		No		NA	
		N	%	N	%	N	%	N	%	N	%	N	%	N	%	N	%	N	%	N	%	N	%	N	%
		N = 21						N = 35						N = 46						N = 49					
**Indicator 1 - Time trend graphs**	Third level of referral	3	75.0	0	0.0	1	25.0	2	66.7	1	33.3	0	0.0	3	60.0	2	40.0	0	0.0	5	100.0	0	0.0	0	0.0
	Second level of referral	0	0.0	0	0.0	3	100.0	6	60.0	4	40.0	0	0.0	5	35.7	8	57.1	1	7.1	10	83.3	2	16.7	0	0.0
	First level of referral	0	0.0	0	0.0	7	100.0	5	45.5	5	45.5	1	9.1	3	33.3	6	66.7	0	0.0	8	66.7	3	25.0	1	8.3
	District/subnational health data office	3	50.0	3	50.0	0	0.0	2	33.3	4	66.7	0	0.0	7	50.0	7	50.0	0	0.0	20	100.0	0	0.0	0	0.0
	Regional/provincial health office	1	100.0	0	0.0	0	0.0	5	100.0	0	0.0	0	0.0	3	75.0	1	25.0	0	0.0						
**Indicator 1 - Bar graphs for comparing facilities, districts, or regions**	Third level of referral	0	0.0	0	0.0	4	100.0	2	66.7	1	33.3	0	0.0	3	60.0	2	40.0	0	0.0	4	80.0	0	0.0	1	20.0
	Second level of referral	0	0.0	0	0.0	3	100.0	5	50.0	5	50.0	0	0.0	5	35.7	8	57.1	1	7.1	10	83.3	2	16.7	0	0.0
	First level of referral	0	0.0	0	0.0	7	100.0	4	36.4	6	54.5	1	9.1	3	33.3	6	66.7	0	0.0	8	66.7	3	25.0	1	8.3
	District/subnational health data office	1	16.7	4	66.7	1	16.7	2	33.3	4	66.7	0	0.0	7	50.0	7	50.0	0	0.0	20	100.0	0	0.0	0	0.0
	Regional/provincial health office	1	100.0	0	0.0	0	0.0	5	100.0	0	0.0	0	0.0	3	75.0	1	25.0	0	0.0						
**Indicator 1 - Thematic maps, by region, district, or health facility**	Third level of referral	0	0.0	0	0.0	4	100.0	2	66.7	1	33.3	0	0.0	2	40.0	3	60.0	0	0.0	4	80.0	0	0.0	1	20.0
	Second level of referral	0	0.0	0	0.0	3	100.0	3	30.0	7	70.0	0	0.0	4	28.6	8	57.1	2	14.3	7	58.3	5	41.7	0	0.0
	First level of referral	0	0.0	0	0.0	7	100.0	4	36.4	6	54.5	1	9.1	3	33.3	6	66.7	0	0.0	3	25.0	8	66.7	1	8.3
	District/subnational health data office	1	16.7	4	66.7	1	16.7	3	37.5	3	37.5	2	25.0	6	42.9	8	57.1	0	0.0	14	70.0	6	30.0	0	0.0
	Regional/provincial health office	1	100.0	0	0.0	0	0.0	3	100.0	0	0.0	0	0.0	3	75.0	1	25.0	0	0.0						

PRISM index	Facility type	Central African Republic	Ethiopia	Tanzania	Uganda
		n=21	n=35	n=46	n=49

**Indicator 1 - Time trend graphs**	Third level of referral	75.0	66.7	60.0	100.0
	Second level of referral	0.0	60.0	35.7	83.3
	First level of referral	0.0	45.5	33.3	66.7
	District/subnational health data office	50.0	33.3	50.0	100.0
	Regional/provincial health office	100.0	100.0	75.0	
**Indicator 1 - Bar graphs for comparing facilities, districts, or regions**	Third level of referral	0.0	66.7	60.0	80.0
	Second level of referral	0.0	50.0	35.7	83.3
	First level of referral	0.0	36.4	33.3	66.7
	District/subnational health data office	16.7	33.3	50.0	100.0
	Regional/provincial health office	100.0	100.0	75.0	
**Indicator 1 - Thematic maps, by region, district, or health facility**	Third level of referral	0.0	66.7	40.0	80.0
	Second level of referral	0.0	30.0	28.6	58.3
	First level of referral	0.0	36.4	33.3	25.0
	District/subnational health data office	16.7	37.5	42.9	70.0
	Regional/provincial health office	100.0	100.0	75.0	

In yellow are marked the indicators that contributed to the calculation via average of the PRISM indexes

eRHIS – electronic routine health information system, NA – not answered, PRISM - performance of routine information system management

Table S18. Practical skills useful for the use of the eRHIS by facility type

	Facility type	Central African Republic		Ethiopia		Tanzania		Uganda	
		Observed score	Max score	Observed score	Max score	Observed score	Max score	Observed score	Max score
		N = 20		N = 98		N = 81		N = 114	
**Calculate percentage pregnant mothers**	District/subnational health data office	3	6	9	11	11	24	22	23
	Regional/provincial health office	0	1	10	10	4	5		
**Calculate neonatal mortality rate**	District/subnational health data office	2	6	7	11	11	24	22	23
	Regional/provincial health office	0	1	7	10	3	5		
**Calculate the number of newborns who died**	District/subnational health data office	1	6	4	11	10	24	22	23
	Regional/provincial health office	0	1	5	10	3	5		
**Calculate KMC coverage**	Third level of referral	1	3	3	9	1	12	3	23
	Second level of referral	1	3	16	32	3	25	14	35
	First level of referral	1	7	13	36	3	15	19	33
**Calculate neonatal mortality rate among males**	Third level of referral	0	3	1	9	0	12	4	23
	Second level of referral	1	3	6	32	3	25	16	35
	First level of referral	1	7	6	36	0	15	16	33
**Calculate neonatal mortality rate among females**	Third level of referral	0	3	1	9	0	12	4	23
	Second level of referral	1	3	7	32	3	25	15	35
	First level of referral	1	7	6	36	0	15	17	33

**Calculate neonatal mortality rate**	Third level of referral	1	3	1	9	1	12	2	23
	Second level of referral	1	3	7	32	1	25	6	35
	First level of referral	1	7	3	36	2	15	4	33
**Calculate the number of newborns who died**	Third level of referral	0	3	2	9	1	12	2	23
	Second level of referral	1	3	5	32	2	25	8	35
	First level of referral	1	7	4	36	1	15	7	33
**Develop a bar chart**	District/subnational health data office	2	6	3	11	8	24	22	23
	Regional/provincial health office	0	1	5	10	5	5		
**Develop a line chart**	Third level of referral	1	3	4	9	0	12	3	23
	Second level of referral	1	3	12	32	5	25	15	35
	First level of referral	0	7	9	36	1	15	21	33
**Develop a trend graph**	Third level of referral	2	3	2	9	0	12	2	23
	Second level of referral	1	3	9	32	3	25	8	35
	First level of referral	1	7	10	36	4	15	8	33
**Interpret the graph**	District/subnational health data office	5	12	15	22	22	48	38	46
	Regional/provincial health office	0	2	17	20	9	10		
**Extract info from graph**	District/subnational health data office	3	12	9	22	12	48	22	46
	Regional/provincial health office	0	2	9	20	4	10		
**Provide guidance from interpretation**	District/subnational health data office	2	12	7	22	12	48	22	46

	Regional/provincial health office	0	2	6	20	4	10		
**Interpret data**	Third level of referral	0	6	5	18	0	24	3	46
	Second level of referral	2	6	14	64	7	50	25	70
	First level of referral	0	14	9	72	5	30	34	66
**Extract neonatal mortality rate**	Third level of referral	0	6	2	18	0	24	3	46
	Second level of referral	1	6	9	64	6	50	16	70
	First level of referral	0	14	6	72	1	30	20	66
**Indicate priority for care improvement**	Third level of referral	1	6	1	18	0	24	2	46
	Second level of referral	1	6	4	64	5	50	14	70
	First level of referral	0	14	4	72	3	30	17	66
**Interpret the graph**	Third level of referral	2	6	1	18	0	24	4	46
	Second level of referral	3	6	11	64	11	50	16	70
	First level of referral	1	14	10	72	6	30	11	66
**Extract info from graph**	Third level of referral	1	6	2	18	1	24	2	46
	Second level of referral	1	6	6	64	6	50	7	70
	First level of referral	1	14	3	72	2	30	5	66
**Describe scenario**	Third level of referral	2	6	10	18	16	24	23	46
	Second level of referral	3	6	24	64	15	50	56	70
	First level of referral	6	14	27	72	11	30	51	66
	District/subnational health data office	5	12	10	22	23	48	45	46
	Regional/provincial health office	0	2	11	20	5	10		

**List problematic data quality aspects**	Third level of referral	5	9	15	27	21	36	33	69
	Second level of referral	5	9	49	96	48	75	74	105
	First level of referral	10	21	44	108	18	45	69	99
	District/subnational health data office	7	18	20	33	43	72	60	69
	Regional/provincial health office	0	3	21	30	10	15		
**Describe plan for quality improvement**	Third level of referral	8	15	26	45	28	60	51	115
	Second level of referral	6	15	61	160	43	125	98	175
	First level of referral	15	35	57	180	23	75	97	165
	District/subnational health data office	7	30	30	55	51	120	95	115
	Regional/provincial health office	0	5	27	50	15	25		
**Provide chart finding at facility level**	District/subnational health data office	2	6	8	11	20	24	22	23
	Regional/provincial health office	0	1	8	10	4	5		
**Provide chart finding at community level**	District/subnational health data office	2	6	7	11	19	24	19	23
	Regional/provincial health office	0	1	5	10	4	5		
**Provide chart finding at district level**	District/subnational health data office	3	6	7	11	17	24	22	23
	Regional/provincial health office	0	1	7	10	5	5		
**Provide chart finding at facility level (1)**	Third level of referral	0	3	4	9	1	12	2	23
	Second level of referral	1	3	10	32	7	25	14	35
	First level of referral	1	7	12	36	4	15	20	33

**Provide chart finding at community level (1)**	Third level of referral	0	3	2	9	0	12	3	23
	Second level of referral	1	3	10	32	6	25	7	35
	First level of referral	0	7	10	36	3	15	14	33
**Provide chart finding at facility level (2)**	Third level of referral	0	3	3	9	0	12	2	23
	Second level of referral	2	3	3	32	7	25	8	35
	First level of referral	2	7	3	36	3	15	7	33
**Provide chart finding at community level (2)**	Third level of referral	0	3	1	9	0	12	2	23
	Second level of referral	2	3	4	32	7	25	8	35
	First level of referral	2	7	3	36	3	15	2	33

**PRISM index**	**Facility type**	Central African Republic	Ethiopia	Tanzania	Uganda
		N = 20	N = 98	N = 81	N = 114
**Calculate percentage pregnant mothers**	District/subnational health data office	50.0	81.8	45.8	95.7
	Regional/provincial health office	0.0	100.0	80.0	
**Calculate neonatal mortality rate**	District/subnational health data office	33.3	63.6	45.8	95.7
	Regional/provincial health office	0.0	70.0	60.0	
**Calculate the number of newborns who died**	District/subnational health data office	16.7	36.4	41.7	95.7
	Regional/provincial health office	0.0	50.0	60.0	
**Calculate KMC coverage**	Third level of referral	33.3	33.3	8.3	13.0
	Second level of referral	33.3	50.0	12.0	40.0

	First level of referral	14.3	36.1	20.0	57.6
**Calculate neonatal mortality rate among males**	Third level of referral	0.0	11.1	0.0	17.4
	Second level of referral	33.3	18.8	12.0	45.7
	First level of referral	14.3	16.7	0.0	48.5
**Calculate neonatal mortality rate among females**	Third level of referral	0.0	11.1	0.0	17.4
	Second level of referral	33.3	21.9	12.0	42.9
	First level of referral	14.3	16.7	0.0	51.5
**Calculate neonatal mortality rate**	Third level of referral	33.3	11.1	8.3	8.7
	Second level of referral	33.3	21.9	4.0	17.1
	First level of referral	14.3	8.3	13.3	12.1
**Calculate the number of newborns who died**	Third level of referral	0.0	22.2	8.3	8.7
	Second level of referral	33.3	15.6	8.0	22.9
	First level of referral	14.3	11.1	6.7	21.2
**Develop a bar chart**	District/subnational health data office	33.3	27.3	33.3	95.7
	Regional/provincial health office	0.0	50.0	100.0	
**Develop a line chart**	Third level of referral	33.3	44.4	0.0	13.0
	Second level of referral	33.3	37.5	20.0	42.9
	First level of referral	0.0	25.0	6.7	63.6
**Develop a trend graph**	Third level of referral	66.7	22.2	0.0	8.7
	Second level of referral	33.3	28.1	12.0	22.9
	First level of referral	14.3	27.8	26.7	24.2

**Interpret the graph**	District/subnational health data office	41.7	68.2	45.8	82.6
	Regional/provincial health office	0.0	85.0	90.0	
**Extract info from graph**	District/subnational health data office	25.0	40.9	25.0	47.8
	Regional/provincial health office	0.0	45.0	40.0	
**Provide guidance from interpretation**	District/subnational health data office	16.7	31.8	25.0	47.8
	Regional/provincial health office	0.0	30.0	40.0	
**Interpret data**	Third level of referral	0.0	27.8	0.0	6.5
	Second level of referral	33.3	21.9	14.0	35.7
	First level of referral	0.0	12.5	16.7	51.5
**Extract neonatal mortality rate**	Third level of referral	0.0	11.1	0.0	6.5
	Second level of referral	16.7	14.1	12.0	22.9
	First level of referral	0.0	8.3	3.3	30.3
**Indicate priority for care improvement**	Third level of referral	16.7	5.6	0.0	4.3
	Second level of referral	16.7	6.3	10.0	20.0
	First level of referral	0.0	5.6	10.0	25.8
**Interpret the graph**	Third level of referral	33.3	5.6	0.0	8.7
	Second level of referral	50.0	17.2	22.0	22.9
	First level of referral	7.1	13.9	20.0	16.7
**Extract info from graph**	Third level of referral	16.7	11.1	4.2	4.3
	Second level of referral	16.7	9.4	12.0	10.0
	First level of referral	7.1	4.2	6.7	7.6
**Describe scenario**	Third level of referral	33.3	55.6	66.7	50.0
	Second level of referral	50.0	37.5	30.0	80.0
	First level of referral	42.9	37.5	36.7	77.3

	District/subnational health data office	41.7	45.5	47.9	97.8
	Regional/provincial health office	0.0	55.0	50.0	
**List problematic data quality aspects**	Third level of referral	55.6	55.6	58.3	47.8
	Second level of referral	55.6	51.0	64.0	70.5
	First level of referral	47.6	40.7	40.0	69.7
	District/subnational health data office	38.9	60.6	59.7	87.0
	Regional/provincial health office	0.0	70.0	66.7	
**Describe plan for quality improvement**	Third level of referral	53.3	57.8	46.7	44.3
	Second level of referral	40.0	38.1	34.4	56.0
	First level of referral	42.9	31.7	30.7	58.8
	District/subnational health data office	23.3	54.5	42.5	82.6
	Regional/provincial health office	0.0	54.0	60.0	
**Provide chart finding at facility level**	District/subnational health data office	33.3	72.7	83.3	95.7
	Regional/provincial health office	0.0	80.0	80.0	
**Provide chart finding at community level**	District/subnational health data office	33.3	63.6	79.2	82.6
	Regional/provincial health office	0.0	50.0	80.0	
**Provide chart finding at district level**	District/subnational health data office	50.0	63.6	70.8	95.7
	Regional/provincial health office	0.0	70.0	100.0	
**Provide chart finding at facility level (1)**	Third level of referral	0.0	44.4	8.3	8.7
	Second level of referral	33.3	31.3	28.0	40.0
	First level of referral	14.3	33.3	26.7	60.6
**Provide chart finding at**	Third level of referral	0.0	22.2	0.0	13.0

**community level (1)**					
	Second level of referral	33.3	31.3	24.0	20.0
	First level of referral	0.0	27.8	20.0	42.4
**Provide chart finding at facility level (2)**	Third level of referral	0.0	33.3	0.0	8.7
	Second level of referral	66.7	9.4	28.0	22.9
	First level of referral	28.6	8.3	20.0	21.2
**Provide chart finding at community level (2)**	Third level of referral	0.0	11.1	0.0	8.7
	Second level of referral	66.7	12.5	28.0	22.9
	First level of referral	28.6	8.3	20.0	6.1

ANC – antenatal care, eRHIS – electronic routine health information system, KMC – kangaroo mother care, PRISM - performance of routine information system management

Table S19. Users' perspective regarding the eRHIS by facility type

Rating given by respondent	Facility type	Central African Republic								Ethiopia							
		Easy to use		Moderate		Difficult to use		Not assessed		Easy to use		Moderate		Difficult to use		Not assessed	
		N	%	N	%	N	%	N	%	N	%	N	%	N	%	N	%
		N = 21								N = 35							
	Third level of referral	4	100.0	0	0.0	0	0.0	0	0.0	2	66.7	1	33.3	0	0.0	0	0.0
	Second level of referral	0	0.0	0	0.0	0	0.0	3	100.0	5	50.0	3	30.0	2	20.0	0	0.0
	First level of referral	0	0.0	0	0.0	0	0.0	7	100.0	4	36.4	5	45.5	1	9.1	1	9.1
	District/subnational health data office	2	33.3	3	50.0	0	0.0	1	16.7	4	66.7	2	33.3	0	0.0	0	0.0
	Regional/provincial health office	1	100.0	0	0.0	0	0.0	0	0.0	4	80.0	1	20.0	0	0.0	0	0.0
		Tanzania								Uganda							
		Easy to use		Moderate		Difficult to use		Not assessed		Easy to use		Moderate		Difficult to use		Not assessed	
		N	%	N	%	N	%	N	%	N	%	N	%	N	%	N	%
		N = 46								N = 49							
	Third level of referral	2	40.0	3	60.0	0	0.0	0	0.0	5	100.0	0	0.0	0	0.0	0	0.0
	Second level of referral	4	28.6	6	42.9	3	21.4	1	7.1	6	50.0	6	50.0	0	0.0	0	0.0
	First level of referral	3	33.3	2	22.2	4	44.4	0	0.0	3	25.0	8	66.7	0	0.0	1	8.3
	District/subnational health data office	6	42.9	8	57.1	0	0.0	0	0.0	15	75.0	5	25.0	0	0.0	0	0.0
	Regional/provincial health office	2	50.0	2	50.0	0	0.0	0	0.0								

Opinion by respondent	Facility type	Central African Republic						Ethiopia					
		Yes		No		Not assessed		Yes		No		Not assessed	
		N	%	N	%	N	%	N	%	N	%	N	%
		N = 21						N = 35					
	Third level of referral	4	100.0	0	0.0	0	0.0	3	100.0	0	0.0	0	0.0
	Second level of referral	0	0.0	0	0.0	3	100.0	10	100.0	0	0.0	0	0.0
	First level of referral	0	0.0	0	0.0	7	100.0	9	81.8	0	0.0	2	18.2
	District/subnational health data office	5	83.3	0	0.0	1	16.7	4	66.7	1	16.7	1	16.7
	Regional/provincial health office	0	0.0	1	100.0	0	0.0	4	80.0	1	20.0		
		Tanzania						Uganda					
		Yes		No		Not assessed		Yes		No		Difficult to use	

		N	%	N	%	N	%	N	%	N	%	N	%
		N = 46						N = 49					
	Third level of referral	5	100.0	0	0.0	0	0.0	3	60.0	2	40.0	0	0.0
	Second level of referral	7	50.0	6	42.9	1	7.1	6	50.0	6	50.0	0	0.0
	First level of referral	5	55.6	4	44.4	0	0.0	6	50.0	5	41.7	1	8.3
	District/subnational health data office	13	92.9	1	7.1	0	0.0	10	50.0	10	50.0	0	0.0
	Regional/provincial health office	4	100.0	0	0.0	0	0.0						

**PRISM index**	**Facility type**	CAR			Ethiopia			Tanzania			Uganda		
**Rating given by respondent**		Easy to use	Moderate	Difficult to use	Easy to use	Moderate	Difficult to use	Easy to use	Moderate	Difficult to use	Easy to use	Moderate	Difficult to use
		%			%			%			%		
		N = 21			N = 35			N = 46			N = 49		
	Third level of referral	100.0	0.0	0.0	66.7	33.3	0.0	40.0	60.0	0.0	100.0	0.0	0.0
	Second level of referral	0.0	0.0	0.0	50.0	30.0	20.0	28.6	42.9	21.4	50.0	50.0	0.0
	First level of referral	0.0	0.0	0.0	36.4	45.5	9.1	33.3	22.2	44.4	25.0	66.7	0.0
	District/subnational health data office	33.3	50.0	0.0	66.7	33.3	0.0	42.9	57.1	0.0	75.0	25.0	0.0
	Regional/provincial health office	100.0	0.0	0.0	80.0	20.0	0.0	50.0	50.0	0.0			

**PRISM index**	**Facility type**	CAR		Ethiopia		Tanzania		Uganda	
**Opinion by respondent**		Yes	No	Yes	No	Yes	No	Yes	No
		%		%		%		%	
		N = 21		N = 35		N = 46		N = 49	
	Third level of referral	100.0	0.0	100.0	0.0	100.0	0.0	60.0	40.0
	Second level of referral	0.0	0.0	100.0	0.0	50.0	42.9	50.0	50.0
	First level of referral	0.0	0.0	81.8	0.0	55.6	44.4	50.0	41.7
	District/subnational health data office	83.3	0.0	66.7	16.7	92.9	7.1	50.0	50.0
	Regional/provincial health office	0.0	100.0	80.0	20.0	100.0	0.0		

In yellow are marked the indicators that contributed to the calculation via average of the PRISM indexes

eRHIS – electronic routine health information system, PRISM - performance of routine information system management

Table S20. Reporting capabilities by region

Assessing and generating	Central African Republic																							
**Facility type**	Bangui City Administration						Health region 1						Health region 2						Health region 7					
	Yes		No		NA		Yes		No		NA		Yes		No		NA		Yes		No		NA	
	N	%	N	%	N	%	N	%	N	%	N	%	N	%	N	%	N	%	N	%	N	%	N	%
**Track report completeness using eRHIS**	N = 21																							
Data offices	1	100	0	0	0	0	0	0	1	100	0	0	2	100	0	0	0	0	3	100	0	0	0	0
Facilities	1	33.3	0	0	2	66.7	0	0	0	0	3	100	0	0	0	0	4	100	0	0	0	0	4	100
	Ethiopia																							
	Addis Ababa City Administration						Amhara and Gambella						Oromia						South Ethiopia and Sidama					
	Yes		No		NA		Yes		No		NA		Yes		No		NA		Yes		No		NA	
	N	%	N	%	N	%	N	%	N	%	N	%	N	%	N	%	N	%	N	%	N	%	N	%
	N = 35																							
Data offices	2	100	0	0	0	0	2	100	0	0	0	0	3	100	0	0	0	0	4	100	0	0	0	0
Facilities	3	100	0	0	0	0	2	66.7	1	33.3	0	0	5	55.6	4	44.4	0	0	0	0	2	22.2	7	77.8
	Tanzania																							
	Dar es Salaam City Administration						Iringa						Shinyanga						Simiyu					
	Yes		No		NA		Yes		No		NA		Yes		No		NA		Yes		No		NA	
	N	%	N	%	N	%	N	%	N	%	N	%	N	%	N	%	N	%	N	%	N	%	N	%
	N = 46																							
Data offices	1	100	0	0	0	0	5	83.3	1	16.7	0	0	5	100	0	0	0	0	6	100	0	0	0	0
Facilities	2	100	0	0	0	0	6	60	3	30	1	10	8	100	0	0	0	0	8	100	0	0	0	0
	Uganda																							
	Kampala City Administration						Karamoja						Lango						West-Nile					
	Yes		No		NA		Yes		No		NA		Yes		No		NA		Yes		No		NA	
	N	%	N	%	N	%	N	%	N	%	N	%	N	%	N	%	N	%	N	%	N	%	N	%
	N = 49																							
Data offices	0	0	0	0	0	0	6	75	2	25	0	0	6	100	0	0	0	0	6	100	0	0	0	0
Facilities	0	0	0	0	1	100	6	75	1	12.5	1	12.5	9	100	0	0	0	0	11	100	0	0	0	0
**Capacity to generate summary reports**	Central African Republic																							
	Bangui City Administration						Health region 1						Health region 2						Health region 7					
**National/regional summary for a year**	Yes		No		NA		Yes		No		NA		Yes		No		NA		Yes		No		NA	
	N	%	N	%	N	%	N	%	N	%	N	%	N	%	N	%	N	%	N	%	N	%	N	%
	N = 21																							
Data offices	1	100	0	0	0	0	0	0	1	100	0	0	0	0	1	50	1	50	0	0	2	66.7	1	33.3
Facilities	0	0	0	0	3	0	0	0	1	33.3	2	66.7	0	0	0	0	4	100	0	0	0	0	4	100
	Ethiopia																							
**National/regional summary for a year**	Addis Ababa City Administration						Amhara and Gambella						Oromia						South Ethiopia and Sidama					
	Yes		No		NA		Yes		No		NA		Yes		No		NA		Yes		No		NA	
	N	%	N	%	N	%	N	%	N	%	N	%	N	%	N	%	N	%	N	%	N	%	N	%
	N = 35																							

Data offices	2	100	0	0	0	0	2	100	0	0	0	0	2	66.7	0	0	1	33.3	1	25	3	75	0	0
Facilities	3	100	0	0	0	0	0	0	0	0	3	100	0	0	0	0	9	100	0	0	0	0	9	100

**National/regional summary for a year**	Tanzania																							
	Dar es Salaam City Administration						Iringa						Shinyanga						Simiyu					
	Yes		No		NA		Yes		No		NA		Yes		No		NA		Yes		No		NA	
	N	%	N	%	N	%	N	%	N	%	N	%	N	%	N	%	N	%	N	%	N	%	N	%
	N = 46																							
Data offices	1	100	0	0	0	0	4	66.7	1	16.7	1	16.7	5	100	0	0	0	0	6	100	0	0	0	0
Facilities	2	100	0	0	0	0	3	30	6	60	1	10	8	100	0	0	0	0	8	100	0	0	0	0

**National/regional summary for a year**	Uganda																							
	Kampala City Administration						Karamoja						Lango						West-Nile					
	Yes		No		NA		Yes		No		NA		Yes		No		NA		Yes		No		NA	
	N	%	N	%	N	%	N	%	N	%	N	%	N	%	N	%	N	%	N	%	N	%	N	%
	N = 49																							
Data offices							4	50	4	50	0	0	2	33.3	3	50	1	16.7	6	100	0	0	0	0
Facilities	1	100	0	0	0	0	3	37.5	4	50	1	12.5	2	22.2	6	66.7	1	11.1	5	45.5	6	54.5	0	0

**District summary for a year**	Central African Republic																							
	Bangui City Administration						Health region 1						Health region 2						Health region 7					
	Yes		No		NA		Yes		No		NA		Yes		No		NA		Yes		No		NA	
	N	%	N	%	N	%	N	%	N	%	N	%	N	%	N	%	N	%	N	%	N	%	N	%
	N = 21																							
Data offices	1	100	0	0	0	0	0	0	1	100	0	0	1	50	1	50	0	0	0	0	3	100	0	0
Facilities	0	0	0	0	3	0	0	0	1	33.3	2	66.7	0	0	0	0	4	100	0	0	0	0	4	100

**District summary for a year**	Ethiopia																							
	Addis Ababa City Administration						Amhara and Gambella						Oromia						South Ethiopia and Sidama					
	Yes		No		NA		Yes		No		NA		Yes		No		NA		Yes		No		NA	
	N	%	N	%	N	%	N	%	N	%	N	%	N	%	N	%	N	%	N	%	N	%	N	%
	N = 35																							
Data offices	1	50	1	0	0	0	2	100	0	0		0	3	100	0	0	0	0	4	100	0	0	0	0
Facilities	3	100	0	0	0	0	0	0	1	33.3	2	66.7	1	11.1	0	0	8	88.9	2	22.2	0	0	7	77.8

**District summary for a year**	Tanzania																							
	Dar es Salaam City Administration						Iringa						Shinyanga						Simiyu					
	Yes		No		NA		Yes		No		NA		Yes		No		NA		Yes		No		NA	
	N	%	N	%	N	%	N	%	N	%	N	%	N	%	N	%	N	%	N	%	N	%	N	%
	N = 46																							
Data offices	1	100	0	0	0	0	6	100	0	0	0	0	5	100	0	0	0	0	6	100	0	0	0	0
Facilities	2	100	0	0	0	0	3	30	6	60	1	10	8	100	0	0	0	0	8	100	0	0	0	0

**District summary for a year**	Uganda																							
	Kampala City Administration						Karamoja						Lango						West-Nile					
	Yes		No		NA		Yes		No		NA		Yes		No		NA		Yes		No		NA	
	N	%	N	%	N	%	N	%	N	%	N	%	N	%	N	%	N	%	N	%	N	%	N	%
	N = 49																							

Data offices							8	100	0	0	0	0	6	100	0	0	0	0	6	100	0	0	0	0
Facilities	0	0	0	0	1	0	7	87.5	0	0	1	12.5	4	44.4	4	0	1	11.1	11	100	0	0	0	0
	Central African Republic																							
**Health facility summary for a year**	Bangui City Administration						Health region 1						Health region 2						Health region 7					
	Yes		No		NA		Yes		No		NA		Yes		No		NA		Yes		No		NA	
	N	%	N	%	N	%	N	%	N	%	N	%	N	%	N	%	N	%	N	%	N	%	N	%
	N =21																							
Data offices	1	100	0	0	0	0	1	100	0	0	0	0	2	100	0	0	0	0	3	100	0	0	0	0
Facilities	3	100	0	0	0	0	1	33.3	0	0	2	66.7	0	0	0	0	4	100	0	0	0	0	4	100
	Ethiopia																							
**Health facility summary for a year**	Addis Ababa City Administration						Amhara and Gambella						Oromia						South Ethiopia and Sidama					
	Yes		No		NA		Yes		No		NA		Yes		No		NA		Yes		No		NA	
	N	%	N	%	N	%	N	%	N	%	N	%	N	%	N	%	N	%	N	%	N	%	N	%
	N = 35																							
Data offices	2	100	0	0	0	0	2	100	0	0	0	0	3	100	0	0	0	0	3	75	1	25	0	0
Facilities	3	100	0	0	0	0	3	100	0	0	0	0	9	100	0	0	0	0	7	77.8	1	11.1	1	11.1
	Tanzania																							
**Health facility summary for a year**	Dar es Salaam City Administration						Iringa						Shinyanga						Simiyu					
	Yes		No		NA		Yes		No		NA		Yes		No		NA		Yes		No		NA	
	N	%	N	%	N	%	N	%	N	%	N	%	N	%	N	%	N	%	N	%	N	%	N	%
	N = 46																							
Data offices	1	100	0	0	0	0	6	100	0	0	0	0	5	100	0	0	0	0	6	100	0	0	0	0
Facilities	2	100	0	0	0	0	8	80	1	10	1	10	8	100	0	0	0	0	8	100	0	0	0	0
	Uganda																							
**Health facility summary for a year**	Kampala City Administration						Karamoja						Lango						West-Nile					
	Yes		No		NA		Yes		No		NA		Yes		No		NA		Yes		No		NA	
	N	%	N	%	N	%	N	%	N	%	N	%	N	%	N	%	N	%	N	%	N	%	N	%
	N = 49																							
Data offices							8	100	0	0	0	0	6	100	0	0	0	0	6	100	0	0	0	0
Facilities	1	100	0	0	0	0	7	87.5	0	0	1	12.5	9	100	0	0	0	0	11	100	0	0	0	0
	Central African Republic																							
**Community-level SDP summary for a year**	Bangui City Administration						Health region 1						Health region 2						Health region 7					
	Yes		No		NA		Yes		No		NA		Yes		No		NA		Yes		No		NA	
	N	%	N	%	N	%	N	%	N	%	N	%	N	%	N	%	N	%	N	%	N	%	N	%
	N = 21																							
Data offices	0	0	1	0	0	0	0	0	1	100	0	0	0	0	2	100	0	0	0	0	3	100	0	0
Facilities	0	0	0	0	3	0	1	33.3	0	0	2	66.7	0	0	0	0	4	100	0	0	0	0	4	100
	Ethiopia																							
**Community-level SDP summary for a year**	Addis Ababa City Administration						Amhara and Gambella						Oromia						South Ethiopia and Sidama					
	Yes		No		NA		Yes		No		NA		Yes		No		NA		Yes		No		NA	
	N	%	N	%	N	%	N	%	N	%	N	%	N	%	N	%	N	%	N	%	N	%	N	%
	N = 35																							
Data offices	1	50	0	0	1	0	2	100	0	0	0	0	2	66.7	0	0	1	33.3	3	75	1	25	0	0

Facilities	0	0	3	0	0	0	0	0	0	0	3	100	2	22.2	3	33.3	4	44.4	3	33.3	1	11.1	5	55.6
	Tanzania																							
**Community-level SDP summary for a year**	Dar es Salaam City Administration						Iringa						Shinyanga						Simiyu					
	Yes		No		NA		Yes		No		NA		Yes		No		NA		Yes		No		NA	
	N	%	N	%	N	%	N	%	N	%	N	%	N	%	N	%	N	%	N	%	N	%	N	%
	N = 46																							
Data offices	0	0	0	0	1	0	0	0	5	83.3	1	16.7	1	20	1	20	3	60	0	0	0	0	6	100
Facilities	1	50	1	0	0	0	0	0	9	90	1	10	0	0	0	0	8	100	2	25	0	0	6	75
	Uganda																							
**Community-level SDP summary for a year**	Kampala City Administration						Karamoja						Lango						West-Nile					
	Yes		No		NA		Yes		No		NA		Yes		No		NA		Yes		No		NA	
	N	%	N	%	N	%	N	%	N	%	N	%	N	%	N	%	N	%	N	%	N	%	N	%
	N = 49																							
Data offices							4	50	4	50	0	0	4	66.7	1	16.7	1	16.7	4	66.7	2	33.3	0	0
Facilities	1	100	0	0	0	0	4	50	3	37.5	1	12.5	3	33.3	5	55.6	1	11.1	4	36.4	7	63.6	0	0

eRHIS – electronic routine health information system, NA – not answered, PRISM - performance of routine information system management, SDP – self-determination program

Table S21. Calculation capabilities by region

Ability to calculate coverage indicators		Central African Republic																							
	**Facility type**	Bangui City Administration						Health region 1						Health region 2						Health region 7					
		Yes		No		NA		Yes		No		NA		Yes		No		NA		Yes		No		NA	
		N	%	N	%	N	%	N	%	N	%	N	%	N	%	N	%	N	%	N	%	N	%	N	%
		N = 21																							
**Neonatal mortality rate - National**	Data offices	1	100	0	0	0	0	0	0	1	100	0	0	0	0	1	50	1	50	0	0	2	66.7	1	33.3
	Facilities	0	0	0	0	3	100	0	0	1	33.3	2	66.7	0	0	0	0	4	100	0	0	0	0.0	4	100.0
**Neonatal mortality rate - Region**	Data offices	1	100	0	0	0	0	0	0	1	100.0	0	0.0	0	0	1	50	1	50	0	0	2	66.7	1	33.3
	Facilities	0	0	0	0	3	100	0	0	1	33.3	2	66.7	0	0	0	0	4	100	0	0	0	0.0	4	100.0
**Neonatal mortality rate - District**	Data offices	1	100	0	0	0	0	0	0	1	100.0	0	0.0	1	50	1	50	0	0	1	33.3	2	66.7	0	0.0
	Facilities	0	0	0	0	3	100	0	0	1	33.3	2	66.7	0	0	0	0	4	100	0	0	0	0.0	4	100.0
**Neonatal mortality rate - Health facility**	Data offices	1	100	0	0	0	0	0	0	1	100.0	0	0.0	2	100	0	0	0	0	3	100	0	0.0	0	0.0
	Facilities	3	100	0	0	0	0	1	33.3	0	0.0	2	66.7	0	0	0	0	4	100	0	0	0	0.0	4	100.0
**Neonatal mortality rate - Community-level SDP**	Data offices	0	0	1	100	0	0	0	0	1	100	0	0	0	0	2	100	0	0	0	0	2	66.7	1	33.3
	Facilities	0	0	0	0	3	100	0	0	1	33.3	2	66.7	0	0	0	0	4	100	0	0	0	0	4	100
		Ethiopia																							
		Addis Ababa City Administration						Amhara and Gambella						Oromia						South Ethiopia and Sidama					
		Yes		No		NA		Yes		No		NA		Yes		No		NA		Yes		No		NA	
		N	%	N	%	N	%	N	%	N	%	N	%	N	%	N	%	N	%	N	%	N	%	N	%
		N = 35																							
**Neonatal mortality rate - National**	Data offices	1	50	1	50	0	0	1	50	0	0	1	50	1	33.3	1	33.3	1	33.3	1	25	2	50	1	25
	Facilities	3	100	0	0	0	0	0	0	0	0	3	100	0	0	0	0	9	100	0	0	1	11.1	8	88.9
**Neonatal mortality rate - Region**	Data offices	2	100	0	0	0	0	2	100	0	0	0	0	1	33.3	1	33.3	1	33.3	2	50	2	50	0	0
	Facilities	3	100	0	0	0	0	0	0	0	0	3	100	0	0	0	0	9	100	0	0	1	11.1	8	88.9
**Neonatal mortality rate - District**	Data offices	2	100	0	0	0	0	2	100	0	0	0	0	2	66.7	1	33.3	0	0.0	4	100	0	0	0	0
	Facilities	3	100	0	0	0	0	0	0	0	0	3	100	1	11.1	0	0	8	88.9	0	0	3	33.3	6	66.7

**Neonatal mortality rate - Health facility**	Data offices	2	100	0	0	0	0	2	100	0	0	0	0	2	66.7	1	33.3	0	0.0	3	75	1	25	0	0
	Facilities	3	100	0	0	0	0	3	100	0	0	0	0	6	66.7	3	33.3	0	0	2	22	6	66.7	1	11.1
**Neonatal mortality rate - Community-level SDP**	Data offices	1	50	0	0	1	50	2	100	0	0	0	0	1	25.0	2	50.0	1	25	1	25	2	50.0	1	25.0
	Facilities	0	0	3	100	0	0	0	0	0	0	3	100	1	11.1	3	33.3	5	55.6	0	0	3	6.0	6	66.7
		Tanzania																							
		Dar es Salaam City Administration						Iringa						Shinyanga						Simiyu					
		Yes		No		NA		Yes		No		NA		Yes		No		NA		Yes		No		NA	
		N	%	N	%	N	%	N	%	N	%	N	%	N	%	N	%	N	%	N	%	N	%	N	%
		N = 46																							
**Neonatal mortality rate - National**	Data offices	1	100	0	0	0	0	0	0	6	100	0	0	2	40	3	60	0	0	3	50	1	16.7	2	33.3
	Facilities	1	50	1	50	0	0	0	0	9	90	1	10	0	0	8	100	0	0	5	62.5	3	37.5	0	0
**Neonatal mortality rate - Region**	Data offices	1	100	0	0	0	0	0	0	6	100	0	0	2	40	3	60	0	0	3	50	1	16.7	2	33.3
	Facilities	1	50	1	50	0	0	0	0	9	90	1	10	0	0	8	100	0	0	5	62.5	3	37.5	0	0
**Neonatal mortality rate - District**	Data offices	1	100	0	0	0	0	1	16.7	5	83.3	0	0	2	40	3	60	0	0	6	100	0	0.0	0	0.0
	Facilities	1	50	1	50	0	0	0	0	9	90	1	10	0	0	8	100	0	0	5	62.5	3	37.5	0	0
**Neonatal mortality rate - Health facility**	Data offices	1	100	0	0	0	0	1	16.7	5	83.3	0	0	2	40	3	60	0	0	6	100	0	0.0	0	0.0
	Facilities	1	50	1	50	0	0	3	30	6	60	1	10	1	12.5	7	87.5	0	0	6	75	2	25	0	0
**Neonatal mortality rate - Community-level SDP**	Data offices	0	0	0	0	1	100	0	0	6	100	0	0	0	0	0	0	5	100	0	0	0	0.0	6	100.0
	Facilities	0	0	2	100	0	0	0	0	9	90	1	10	0	0	0	0	8	100	0	0	1	12.5	7	87.5
		Uganda																							
		Kampala City Administration						Karamoja						Lango						West-Nile					
		Yes		No		NA		Yes		No		NA		Yes		No		NA		Yes		No		NA	
		N	%	N	%	N	%	N	%	N	%	N	%	N	%	N	%	N	%	N	%	N	%	N	%
		N = 49																							
**Neonatal mortality rate - National**	Data offices							3	37.5	5	62.5	0	0	0	0	4	66.7	2	33.3	6	100	0	0	0	0
	Facilities	0	0	0	0	1	100	1	12.5	6	75	1	12.5	1	11.1	6	66.7	2	22.2	3	27.3	8	72.7	0	0
**Neonatal mortality rate - Region**	Data offices							4	50	4	50	0	0	3	50	1	16.7	2	33.3	6	100	0	0	0	0
	Facilities	0	0	0	0	1	100	2	25	5	62.5	1	12.5	2	22.2	6	66.7	1	11.1	5	45.5	6	54.5	0	0

**Neonatal mortality rate - District**	Data offices							8	100	0	0	0	0	6	100	0	0.0	0	0.0	6	100	0	0	0	0
	Facilities	0	0	0	0	1	100	6	75	1	12.5	1	12.5	5	55.6	3	33.3	1	11.1	8	72.7	3	27.3	0	0
**Neonatal mortality rate - Health facility**	Data offices							8	100	0	0	0	0	6	100	0	0.0	0	0.0	6	100	0	0	0	0
	Facilities	1	100	0	0	0	0	6	75	1	12.5	1	12.5	9	100.0	0	0.0	0	0.0	9	81.8	2	18.2	0	0
**Neonatal mortality rate - Community-level SDP**	Data offices							3	37.5	5	62.5	0	0	0	0	6	100.0	0	0.0	3	50	2	33.3	1	16.7
	Facilities	0	0	0	0	1	100	3	37.5	4	50	1	12.5	3	33.3	5	55.6	1	11.1	5	45.5	6	54.5	0	0

NA – not answered, SDP – self determination program

Table S22. Data analysis capabilities by region

Percentage of staff demonstrating the use of data analysis features	Facility type	Central African Republic																							
		Bangui City Administration						Health region 1						Health region 2						Health region 7					
		Yes		No		NA		Yes		No		NA		Yes		No		NA		Yes		No		NA	
		N	%	N	%	N	%	N	%	N	%	N	%	N	%	N	%	N	%	N	%	N	%	N	%
		N = 21																							
**Major causes neonatal mortality**	Data offices	0	0	1	100	0	0	0	0	1	100	0	0	0	0	2	100	0	0	0	0	3	100	0	0
	Facilities	1	33.3	2	66.7	0	0	0	0	1	33.3	2	66.7	0	0	0	0	4	100	0	0	0	0	4	100
		Ethiopia																							
		Addis Ababa City Administration						Amhara and Gambella						Oromia						South Ethiopia and Sidama					
		Yes		No		NA		Yes		No		NA		Yes		No		NA		Yes		No		NA	
		N	%	N	%	N	%	N	%	N	%	N	%	N	%	N	%	N	%	N	%	N	%	N	%
		N = 35																							
	Data offices	0	0	2	100	0	0	0	0	2	100	0	0	0	0	3	100	0	0	0	0	4	100	0	0
	Facilities	2	66.7	1	33.3	0	0	2	66.7	1	33.3	0	0	1	11.1	8	88.9	0	0	0	0	7	77.8	2	22.2
		Tanzania																							
		Dar es Salaam City Administration						Iringa						Shinyanga						Simiyu					
		Yes		No		NA		Yes		No		NA		Yes		No		NA		Yes		No		NA	
		N	%	N	%	N	%	N	%	N	%	N	%	N	%	N	%	N	%	N	%	N	%	N	%
		N = 46																							
	Data offices	1	100	0	0	0	0	6	100	0	0	0	0	3	60	2	40	0	0	1	16.7	5	83.3	0	0
	Facilities	2	100	0	0	0	0	3	30	6	60	1	10	0	0	8	100	0	0	2	25	6	75	0	0
		Uganda																							
		Kampala City Administration						Karamoja						Lango						West-Nile					
		Yes		No		NA		Yes		No		NA		Yes		No		NA		Yes		No		NA	
		N	%	N	%	N	%	N	%	N	%	N	%	N	%	N	%	N	%	N	%	N	%	N	%
		N = 49																							
	Data offices							6	75	2	25	0	0	5	83.3	1	16.7	0	0	6	100	0	0	0	0
	Facilities	1	100	0	0	0	0	6	75	1	12.5	1	12.5	5	55.6	4	44.4	0	0	9	81.8	2	18.2	0	0
**Major causes neonatal morbidity**		Central African Republic																							
		Bangui City Administration						Health region 1						Health region 2						Health region 7					
		Yes		No		NA		Yes		No		NA		Yes		No		NA		Yes		No		NA	
		N	%	N	%	N	%	N	%	N	%	N	%	N	%	N	%	N	%	N	%	N	%	N	%
		N = 21																							
	Data offices	0	0	1	100	0	0	0	0	1	100	0	0	0	0	2	100	0	0	0	0	3	100	0	0
	Facilities	0	0	2	66.7	1	33.3	0	0	1	33.3	2	66.7	0	0	0	0	4	100	0	0	0	0	4	100
		Ethiopia																							
		Addis Ababa City Administration						Amhara and Gambella						Oromia						South Ethiopia and Sidama					
		Yes		No		NA		Yes		No		NA		Yes		No		NA		Yes		No		NA	

	N	%	N	%	N	%	N	%	N	%	N	%	N	%	N	%	N	%	N	%	N	%	N	%
	N = 35																							
Data offices	0	0	2	100	0	0	0	0	2	100	0	0	0	0	3	100	0	0	2	50	2	50	0	0
Facilities	2	66.7	1	33.3	0	0.0	2	66.7	1	33.3	0	0.0	1	11.1	8	88.9	0	0	1	11.1	6	66.7	2	22.2
	Tanzania																							
	Dar es Salaam City Administration						Iringa						Shinyanga						Simiyu					
	Yes		No		NA		Yes		No		NA		Yes		No		NA		Yes		No		NA	
	N	%	N	%	N	%	N	%	N	%	N	%	N	%	N	%	N	%	N	%	N	%	N	%
	N = 46																							
Data offices	1	100	0	0	0	0	6	100	0	0	0	0	1	20	3	60	1	20	1	16.7	5	83.3	0	0
Facilities	2	100	0	0.0	0	0.0	2	20.0	7	70.0	1	10.0	0	0.0	8	100	0	0	2	25.0	6	75.0	0	0.0
	Uganda																							
	Kampala City Administration						Karamoja						Lango						West-Nile					
	Yes		No		NA		Yes		No		NA		Yes		No		NA		Yes		No		NA	
	N	%	N	%	N	%	N	%	N	%	N	%	N	%	N	%	N	%	N	%	N	%	N	%
	N = 49																							
Data offices							7	87.5	1	12.5	0	0	6	100	0	0	0	0	6	100	0	0	0	0
Facilities	1	100	0	0.0	0	0.0	6	75.0	1	12.5	1	12.5	4	44.4	5	55.6	0	0	8	72.7	3	27.3	0	0.0
**Data disaggregation**	Central African Republic																							
	Bangui City Administration						Health region 1						Health region 2						Health region 7					
	Yes		No		NA		Yes		No		NA		Yes		No		NA		Yes		No		NA	
	N	%	N	%	N	%	N	%	N	%	N	%	N	%	N	%	N	%	N	%	N	%	N	%
	N = 21																							
Data offices	1	100	0	0	0	0	0	0	1	100	0	0	0	0	2	100	0	0	1	33.3	2	66.7	0	0
Facilities	2	66.7	1	33.3	0	0.0	0	0.0	0	0.0	3	100	0	0.0	0	0.0	4	100	0	0.0	0	0.0	4	100
	Ethiopia																							
	Addis Ababa City Administration						Amhara and Gambella						Oromia						South Ethiopia and Sidama					
	Yes		No		NA		Yes		No		NA		Yes		No		NA		Yes		No		NA	
	N	%	N	%	N	%	N	%	N	%	N	%	N	%	N	%	N	%	N	%	N	%	N	%
	N = 35																							
Data offices	0	0	2	100		0	0	0	2	100	0	0	0	0	3	100	0	0	0	0	4	100	0	0
Facilities	2	66.7	1	33.3	0	0.0	0	0.0	3	100	0	0.0	0	0.0	9	100	0	0	0	0.0	7	77.8	2	22.2
	Tanzania																							
	Dar es Salaam City Administration						Iringa						Shinyanga						Simiyu					
	Yes		No		NA		Yes		No		NA		Yes		No		NA		Yes		No		NA	
	N	%	N	%	N	%	N	%	N	%	N	%	N	%	N	%	N	%	N	%	N	%	N	%
	N = 46																							
Data offices	0	0	1	100	0	0	0	0	6	100	0	0	3	60	2	40	0	0	5	83.3	1	16.7	0	0
Facilities	1	50.0	1	50.0	0	0.0	6	60.0	3	30.0	1	10.0	0	0.0	8	100	0	0	3	37.5	5	62.5	0	0.0
	Uganda																							
	Kampala City Administration						Karamoja						Lango						West-Nile					
	Yes		No		NA		Yes		No		NA		Yes		No		NA		Yes		No		NA	
	N	%	N	%	N	%	N	%	N	%	N	%	N	%	N	%	N	%	N	%	N	%	N	%
	N = 49																							
Data offices							6	75	2	25	0	0	4	66.7	2	33.3	0	0	5	83.3	1	16.7	0	0

	Facilities	1	100	0	0.0	0	0.0	4	50.0	3	37.5	1	12.5	5	55.6	4	44.4	0	0	4	36.4	7	63.6	0	0.0

Table S23. Visualization capabilities by region

**Percentage of staff able to use the data visualization features of the eRHIS to analyse and present data in graphs and maps**	Central African Republic																							
**Indicator 1 - Time trend graphs**	Bangui City Administration						Health region 1						Health region 2						Health region 7					
	Yes		No		NA		Yes		No		NA		Yes		No		NA		Yes		No		NA	
	N	%	N	%	N	%	N	%	N	%	N	%	N	%	N	%	N	%	N	%	N	%	N	%
	N = 21																							
	1	100	0	0	0	0	0	0	1	0	0	0	0	0	2	0	0	0	3	100	0	0	0	0
	3	100	0	0	0	0	0	0	0	0	3	100	0	0	0	0	4	100	0	0	0	0	4	100
	Ethiopia																							
	Addis Ababa City Administration						Amhara and Gambella						Oromia						South Ethiopia and Sidama					
	Yes		No		NA		Yes		No		NA		Yes		No		NA		Yes		No		NA	
	N	%	N	%	N	%	N	%	N	%	N	%	N	%	N	%	N	%	N	%	N	%	N	%
	N = 35																							
	2.0	100	0.0	0.0	0.0	0.0	2.0	100	0.0	0.0	0.0	0.0	2.0	66.7	1.0	0.0	0.0	0.0	1.0	25.0	3.0	0.0	0.0	0.0
	3.0	100	0.0	0.0	0.0	0.0	2.0	66.7	1.0	0.0	0.0	0.0	6.0	66.7	3.0	0.0	0.0	0.0	2.0	22.2	6.0	0.0	1.0	11.1
	Tanzania																							
	Dar es Salaam City Administration						Iringa						Shinyanga						Simiyu					
	Yes		No		NA		Yes		No		NA		Yes		No		NA		Yes		No		NA	
	N	%	N	%	N	%	N	%	N	%	N	%	N	%	N	%	N	%	N	%	N	%	N	%
	N = 46																							
	1	100	0	0	0	0	0	0	6	0	0	0	3	60	2	0	0	0	6	100	0	0	0	0
	1	50	1	0	0	0	4	40	5	0	1	10	0	0	8	0	0	0	6	75	2	0	0	0
	Uganda																							
	Kampala City Administration						Karamoja						Lango						West-Nile					
	Yes		No		NA		Yes		No		NA		Yes		No		NA		Yes		No		NA	
	N	%	N	%	N	%	N	%	N	%	N	%	N	%	N	%	N	%	N	%	N	%	N	%
	N = 49																							
							8	100	0	0	0	0	6	100	0	0	0	0	6	100	0	0	0	0
	1	100	0	0	0	0	6	75	1	0	1	12.5	6	66.7	3	0	0	0	10	90.9	1	0	0	0
**Indicator 1 - Bar graphs for comparing facilities, districts, or regions**	Central African Republic																							
	Bangui City Administration						Health region 1						Health region 2						Health region 7					
	Yes		No		NA		Yes		No		NA		Yes		No		NA		Yes		No		NA	
	N	%	N	%	N	%	N	%	N	%	N	%	N	%	N	%	N	%	N	%	N	%	N	%
	N = 21																							
	1	100	0	0	0	0	0	0	1	0	0	0	0	0	1	0	1	50	0	0	2	0	0	0
	0	0	0	0	3	100	0	0	0	0	3	100	0	0	0	0	4	100	0	0	0	0	4	100
	Ethiopia																							
	Addis Ababa City Administration						Amhara and Gambella						Oromia						South Ethiopia and Sidama					
	Yes		No		NA		Yes		No		NA		Yes		No		NA		Yes		No		NA	
	N	%	N	%	N	%	N	%	N	%	N	%	N	%	N	%	N	%	N	%	N	%	N	%

	N = 35																							
	2.0	100	0.0	0.0	0.0	0.0	2.0	100	0.0	0.0	0.0	0.0	2.0	66.7	1.0	0.0	0.0	0.0	1.0	25.0	3.0	0.0	0.0	0.0
	2.0	66.7	1.0	0.0	0.0	0.0	2.0	66.7	1.0	0.0	0.0	0.0	6.0	66.7	3.0	0.0	0.0	0.0	1.0	11.1	7.0	0.0	1.0	11.1
	Tanzania																							
	Dar es Salaam City Administration						Iringa						Shinyanga						Simiyu					
	Yes		No		NA		Yes		No		NA		Yes		No		NA		Yes		No		NA	
	N	%	N	%	N	%	N	%	N	%	N	%	N	%	N	%	N	%	N	%	N	%	N	%
	N = 46																							
	1	100	0	0	0	0	0	0	6	0	0	0	3	60	2	0	0	0	6	100	0	0	0	0
	1	50	1	0	0	0	5	50	4	0	1	10	0	0	8	0	0	0	5	62.5	3	0	0	0
	Uganda																							
	Kampala City Administration						Karamoja						Lango						West-Nile					
	Yes		No		NA		Yes		No		NA		Yes		No		NA		Yes		No		NA	
	N	%	N	%	N	%	N	%	N	%	N	%	N	%	N	%	N	%	N	%	N	%	N	%
	N = 49																							
							8	100	0	0	0	0	6	100	0	0	0	0	6	100	0	0	0	0
	0	0	0	0	1	100	6	75	1	0	1	12.5	6	66.7	3	0	0	0	10	90.9	1	0	0	0
**Indicator 1 - Thematic maps, by region, district, or health facility**	Central African Republic																							
	Bangui City Administration						Health region 1						Health region 2						Health region 7					
	Yes		No		NA		Yes		No		NA		Yes		No		NA		Yes		No		NA	
	N	%	N	%	N	%	N	%	N	%	N	%	N	%	N	%	N	%	N	%	N	%	N	%
	N= 21																							
	1.0	100	0.0	0.0	0.0	0.0	0.0	0.0	1.0	0.0	0.0	0.0	0.0	0.0	1.0	0.0	1.0	50.0	1.0	33.3	2.0	0.0	0.0	0.0
	0.0	0.0	0.0	0.0	3.0	100	0.0	0.0	0.0	0.0	3.0	100	0.0	0.0	0.0	0.0	2.0	100	0.0	0.0	0.0	0.0	4.0	100
	Ethiopia																							
	Addis Ababa City Administration						Amhara and Gambella						Oromia						South Ethiopia and Sidama					
	Yes		No		NA		Yes		No		NA		Yes		No		NA		Yes		No		NA	
	N	%	N	%	N	%	N	%	N	%	N	%	N	%	N	%	N	%	N	%	N	%	N	%
	N = 35																							
	1.0	50.0	0.0	0.0	1.0	50.0	2.0	100	0.0	0.0	0.0	0.0	2.0	66.7	0.0	0.0	1.0	33.3	1.0	25.0	3.0	0.0	0.0	0.0
	1.0	33.3	2.0	0.0	0.0	0.0	2.0	66.7	1.0	0.0	0.0	0.0	6.0	66.7	3.0	0.0	0.0	0.0	0.0	0.0	8.0	0.0	1.0	11.1
	Tanzania																							
	Dar es Salaam City Administration						Iringa						Shinyanga						Simiyu					
	Yes		No		NA		Yes		No		NA		Yes		No		NA		Yes		No		NA	
	N	%	N	%	N	%	N	%	N	%	N	%	N	%	N	%	N	%	N	%	N	%	N	%
	N = 46																							
	1	100	0	0	0	0	0	0	6	0	0	0	2	40	3	0	0	0	6	100	0	0	0	0
	1	50	1	0	0	0	2	20	7	0	1	10	0	0	7	0	1	12.5	6	75	2	0	0	0
	Uganda																							
	Kampala City Administration						Karamoja						Lango						West-Nile					
	Yes		No		NA		Yes		No		NA		Yes		No		NA		Yes		No		NA	
	N	%	N	%	N	%	N	%	N	%	N	%	N	%	N	%	N	%	N	%	N	%	N	%
	N = 49																							
							5	62.5	3	0	0	0	3	50	3	0	0	0	6	100	0	0	0	0

	0	0	0	0	1	100	4	50	3	0	1	12.5	4	44.4	5	0	0	0	6	54.5	5	0	0	0

eRHIS – electronic routine health information system, NA – not answered

Table S24. Users' perspective by region

Rating given by respondent	Facility type	Central African Republic							
		Bangui City Administration		Health region 1		Health region 2		Health region 7	
		N	%	N	%	N	%	N	%
		N = 21							
**Data offices**	Easy to use	1	100.0	0	0	1	50	1	33.3
	Moderate	0	0.0	1	100	0	0	2	66.7
	Difficult to use	0	0.0	0	0	0	0	0	0
	Not assessed	0	0.0	0	0	1	50	0	0
**Facilities**	Easy to use	3	100.0	1	33.3	0	0.0	0	0.0
	Moderate	0	0.0	0	0.0	0	0.0	0	0.0
	Difficult to use	0	0.0	0	0.0	0	0.0	0	0.0
	Not assessed	0	0.0	2	66.7	4	100.0	4	100.0
		Ethiopia							
		Addis Ababa City Administration		Amhara and Gambella		Oromia		South Ethiopia and Sidama	
		N	%	N	%	N	%	N	%
		N = 35							
**Data offices**	Easy to use	1	50.0	2	100	3	100	2	50.0
	Moderate	1	50.0	0	0	0	0	2	50.0
	Difficult to use	0	0.0	0	0	0	0	0	0
	Not assessed	0	0.0	0	0	0	0	0	0
**Facilities**	Easy to use	1	33.3	1	33.3	5	55.6	4	44.4
	Moderate	1	33.3	2	66.7	3	33.3	3	33.3
	Difficult to use	1	33.3	0	0.0	1	11.1	1	11.1
	Not assessed	0	0.0	0	0.0	0	0.0	1	11.1
		Tanzania							
		Dar es Salaam City Administration		Iringa		Shinyanga		Simiyu	
		N	%	N	%	N	%	N	%
		N = 46							
**Data offices**	Easy to use	1	100.0	0	0	3	60	4	66.7
	Moderate	0	0.0	6	100	2	40	2	33.3
	Difficult to use	0	0.0	0	0	0	0	0	0
	Not assessed	0	0.0	0	0	0	0	0	0
**Facilities**	Easy to use	0	0.0	5	50.0	0	0.0	4	50.0
	Moderate	2	100.0	1	10.0	4	50.0	4	50.0
	Difficult to use	0	0.0	3	30.0	4	50.0	0	0.0
	Not assessed	0	0.0	1	10.0	0	0.0	0	0.0
		Uganda							
		Kampala City Administration		Karamoja		Lango		West-Nile	
		N	%	N	%	N	%	N	%
		N = 49							
**Data offices**	Easy to use	0	0.0	6	75	4	66.7	5	83.3
	Moderate	0	0.0	2	25	2	33.3	1	16.7
	Difficult to use	0	0.0	0	0	0	0	0	0

	Not assessed	0	0.0	0	0	0	0	0	0
**Facilities**	Easy to use	1	100.0	5	62.5	3	33.3	5	45.5
	Moderate	0	0.0	2	25.0	6	66.7	6	54.5
	Difficult to use	0	0.0	0	0.0	0	0.0	0	0.0
	Not assessed	0	0.0	1	12.5	0	0.0	0	0.0
**Opinion by respondent**		Central African Republic							
		Bangui City Administration		Health region 1		Health region 2		Health region 7	
		N	%	N	%	N	%	N	%
		N = 21							
**Data offices**	Yes	0	0.0	1	100	1	50.0	3	100.0
	No	1	100.0	0	0	0	0.0	0	0.0
	Not assessed	0	0.0	0	0	1	50	0	0
**Facilities**	Yes	3	100.0	1	33.3	0	0	0	0
	No	0	0.0	0	0.0	0	0.0	0	0.0
	Not assessed	0	0.0	2	66.7	4	100.0	4	100.0
		Ethiopia							
		Addis Ababa City Administration		Amhara and Gambella		Oromia		South Ethiopia and Sidama	
		N	%	N	%	N	%	N	%
		N = 35							
**Data offices**	Yes	2	100.0	2	100	1	33.3	3	75.0
	No	0	0.0	0	0	2	66.7	0	0.0
	Not assessed	0	0.0	0	0	0	0	1	25
**Facilities**	Yes	3	100.0	3	100	9	100	7	77.8
	No	0	0.0	0	0.0	0	0.0	0	0.0
	Not assessed	0	0.0	0	0.0	0	0.0	2	22.2
		Tanzania							
		Dar es Salaam City Administration		Iringa		Shinyanga		Simiyu	
		N	%	N	%	N	%	N	%
		N = 46							
**Data offices**	Yes	1	100.0	6	100	4	80.0	6	100.0
	No	0	0.0	0	0	1	20.0	0	0.0
	Not assessed	0	0.0	0	0	0	0	0	0
**Facilities**	Yes	2	100.0	8	80	2	25.00	5	62.5
	No	0	0.0	1	10.0	6	75.0	3	37.5
	Not assessed	0	0.0	1	10.0	0	0.0	0	0.0
		Uganda							
		Kampala City Administration		Karamoja		Lango		West-Nile	
		N	%	N	%	N	%	N	%
		N = 49							
**Data offices**	Yes			4	50	2	33.3	4	66.7
	No			4	50	4	66.7	2	33.3
	Not assessed			0	0	0	0	0	0
**Facilities**	Yes	0	0.0	5	62.5	2	22.22	8	72.7
	No	1	100.0	2	25.0	7	77.8	3	27.3
	Not assessed	0	0.0	1	12.5	0	0.0	0	0.0

Table S25. Open question

Question: Describe any improvement you would like to see in the eRHIS					
**Country**	**Comments (free text answers) ORIGINAL**	**Comments (free text answers) EXTRACTED**	**Major themes**	**Main themes**	**Sub-themes**
Central African Republic	Désagrégation par sexe, âge, niveau d'étude, statut socio-économique, provenance etc - Disaggregation by sex, age, level of study, socio-economic status, origin etc		Improve technical aspects	Improve functionalities for data quality checks, and data analysis	Data disaggregation
Central African Republic	Désagréger par sexe dans le DHIS2 - Disaggregate by sex in DHIS2 Uniformiser les données collectées du registre et celles compilées dans le DHIS2 - Standardize the data collected from the register and those compiled in the DHIS2	Désagréger par sexe dans le DHIS2 - Disaggregate by sex in DHIS2	Improve technical aspects	Improve functionalities for data quality checks, and data analysis	Data disaggregation
		Uniformiser les données collectées du registre et celles compilées dans le DHIS2 - Standardize the data collected from the register and those compiled in the DHIS2	Improve technical aspects	Improve functionalities for data quality checks, and data analysis	Data standardization
Central African Republic	Développer spécifiquement une application WEB pour la gestion des collectes des données. - Specifically develop a WEB application for managing data collection.		Improve technical aspects	Technical/software improvement	Software development
Central African Republic	Fiabilité des données - Data reliability		Improve technical aspects	Improve functionalities for data quality checks, and data analysis	Data quality
Central African Republic	Personnel formé et recyclé - Trained and retrained staff		Capacity strengthening	Training improvement	Staff training
Central African Republic	Introduction dans le formulaire les causes des décès uniquement de la maternité - Introduction in the form of the causes of death only in the maternity ward		Improve technical aspects	Technical/software improvement	Software development
Central African Republic	Introduire dans le logiciel DHIS2 d'autres indicateurs comme le genre, statut socioéconomique des parents, causes de mortalité, provenance...etc. - Introduce other indicators into the DHIS2 software such as gender, socio-economic status of parents, causes of mortality, origin, etc.		Improve technical aspects	Technical/software improvement	Software development
Central African Republic	Mettre en place le logiciel DHIS2 - Set up the DHIS2 software		Improve technical aspects	Technical/software improvement	Improve software usability
Central African Republic	Permettre au fichier excel de produire automatiquement des graphes de tendance - Allow the excel file to automatically produce trend graphs		Improve technical aspects	Improve functionalities for data quality checks, and data analysis	Improve graphs generation
Central African Republic	Paramétrer les données démographiques dans le logiciel pour faciliter le calcul des pourcentages - Configure demographic data in the software to facilitate the calculation of percentages		Improve technical aspects	Improve functionalities for data quality	Improve indicators calculation

				checks, and data analysis	
Central African Republic	Travailler en collaboration avec le major de service de maternité pour améliorer l'utilisation du registre. - Work in collaboration with the maternity service major to improve the use of the register.		Improve technical aspects	Technical/software improvement	Send direct feedback
Ethiopia	After entering and submitting all the data that is required it usually says the data is incomplete and it doesn't show which data are missing.		Improve technical aspects	Technical/software improvement	Software bug fixes
Ethiopia	Analysis of indicators need improvement in the software after importing data there is difficulty to analyse indicators it will not allow us to analyse the data immediately there is some delay.		Improve technical aspects	Technical/software improvement	Improve software capacity
Ethiopia	Automation of DHIS with other systems in the hospital		Improve technical aspects	Technical/software improvement	Integration of DHIS with other data systems
Ethiopia	Being able to calculate indicators. Doesn't give us a map of which specific area of the catchment has the most referals. Challenge in target setting using the electronic system (we largely use Excel or PowerPoint)	Being able to calculate indicators.	Improve technical aspects	Improve functionalities for data quality checks, and data analysis	Improve indicators calculation
		Doesn't give us a map of which specific area of the catchment has the most referals.	Improve technical aspects	Improve functionalities for data quality checks, and data analysis	Improve cartographs generation
		Challenge in target setting using the electronic system (we largely use Excel or PowerPoint)	Improve technical aspects	Technical/software improvement	Improve functionality to calculate targets
Ethiopia	Custome age disaggregation should be allowed , sometimes after editing data on dhis 2 it doesn't save the data and it displays the unedited data , if you edit data it will change the timeliness of the report to zero and the down arrow of the keyboard doesn't work forcing us to use the mouse which increases our workload.	Custome age disaggregation should be allowed	Improve technical aspects	Improve functionalities for data quality checks, and data analysis	Data disaggregation
		sometimes after editing data on dhis 2 it doesn't save the data and it displays the unedited data ,	Improve technical aspects	Technical/software improvement	Software bug fixes
		if you edit data it will change the timeliness of the report to zero and the down arrow of the keyboard doesn't work forcing us to use the mouse which increases our workload.	Improve technical aspects	Technical/software improvement	Improve software usability
Ethiopia	DHIS2 should be able to calculate low Birthweight rate		Improve technical aspects	Improve functionalities for data quality checks, and data analysis	Improve indicators calculation (low birthweight)
Ethiopia	Data visualisation should be user friendly and it should be capable of calculating top ten causes of neonatal Mortality.	Data visualisation should be user friendly	Improve technical aspects	Improve functionalities for data quality checks, and data analysis	Improve graphs generation

		and it should be capable of calculating top ten causes of neonatal Mortality.	Improve technical aspects	Improve functionalities for data quality checks, and data analysis	Improve indicators calculation (neonatal mortality)
Ethiopia	Dhis 2 Should be capable of calculating timeliness, the down arrow on the keyboard is not recognised by dhis 2 this forced us to use mouse so it is difficult to enter or edit data and neonatal data disaggregation by sex should be functional.	Dhis 2 Should be capable of calculating timeliness	Improve technical aspects	Improve functionalities for data quality checks, and data analysis	Improve indicators calculation
		, the down arrow on the keyboard is not recognised by dhis 2 this forced us to use mouse so it is difficult to enter or edit data	Improve technical aspects	Technical/software improvement	Improve software usability
		and neonatal data disaggregation by sex should be functional.	Improve technical aspects	Improve functionalities for data quality checks, and data analysis	Data disaggregation
Ethiopia	Dhis 2 should be capable to check completeness of the report , it should be capable of calculating top cause of morbidity and mortality for neonates and it should be capable of calculating indicators.	Dhis 2 should be capable to check completeness of the report	Improve technical aspects	Improve functionalities for data quality checks, and data analysis	Data quality
		, it should be capable of calculating top cause of morbidity and mortality for neonates and it should be capable of calculating indicators.	Improve technical aspects	Improve functionalities for data quality checks, and data analysis	Improve indicators calculation (neonatal morbidity and mortality)
Ethiopia	Dhis2 doesn't calculate some of the neonatal indicators even though the data is available and no clear source document for reportable data elements like neonatal asphyxia and when the dhis 2 software undergoes update we must be briefed.	Dhis2 doesn't calculate some of the neonatal indicators even though the data is available and no clear source document for reportable data elements like neonatal asphyxia	Improve technical aspects	Technical/software improvement	Software bug fixes
		and when the dhis 2 software undergoes update we must be briefed.	Improve technical aspects	Technical/software improvement	Improve software usability
Ethiopia	During data analysis and generating report dhis 2 has some problems it displays wrong data that can underestimate our performance, some of the disease types are not included in the dhis 2 disease list and the down arrow of the keyboard doesn't work forcing us to use mouse for data entry that has significant impact on the work load. The above problems should be fixed.	During data analysis and generating report dhis 2 has some problems it displays wrong data that can underestimate our performance	Improve technical aspects	Technical/software improvement	Software bug fixes
		some of the disease types are not included in the dhis 2 disease list	Improve technical aspects	Technical/software improvement	Software bug fixes
		and the down arrow of the keyboard doesn't work forcing us to use mouse for data entry that has significant impact on the work load. The above problems should be fixed.	Improve technical aspects	Technical/software improvement	Improve software usability
Ethiopia	If EDHIS2 generate low birth weight,KMC, Morbidity, Sepsis and early intiation of breastfeeding		Improve technical aspects	Improve functionalities for data quality checks, and data analysis	Improve indicators calculation (newborn indicators)

Ethiopia	If the revised EDHIS2, backup and show the past year and month report,eg it doesn't show the 2021,2020 and 2022 newborn and stillbirth data element report.		Improve technical aspects	Technical/software improvement	Software development
Ethiopia	In the dhis 2 Labeling of wards should be with their standard name and the server speed needs major upgrade during data entry and reporting period the server is very slow and majority of the time we are forced to wait for the pages to load.	In the dhis 2 Labeling of wards should be with their standard name	Improve technical aspects	Improve functionalities for data quality checks, and data analysis	Data standardization
		and the server speed needs major upgrade during data entry and reporting period the server is very slow and majority of the time we are forced to wait for the pages to load.	Improve technical aspects	Technical/software improvement	Improve software capacity
Ethiopia	It should be capable of calculating indicators		Improve technical aspects	Improve functionalities for data quality checks, and data analysis	Improve indicators calculation
Ethiopia	Missing		No suggestion given	No suggestion given	No suggestion given
Ethiopia	No age category for neonates and it should be disaggregated by age but it is not. Data generation is sometimes inconsistent	No age category for neonates and it should be disaggregated by age but it is not.	Improve technical aspects	Improve functionalities for data quality checks, and data analysis	Data disaggregation
		Data generation is sometimes inconsistent	Improve technical aspects	Improve functionalities for data quality checks, and data analysis	Data quality
Ethiopia	Some of the data elements in the dhis 2 needs to be clarified.		Improve technical aspects	Technical/software improvement	Improve software usability
Ethiopia	Technical error of the DHIS 2 (It makes errors which result in discrepancies)		Improve technical aspects	Technical/software improvement	Software bug fixes
Ethiopia	The ECV 11 classification disease list is not fully entered into DHIS so the data coming from the wards is not added		Improve technical aspects	Technical/software improvement	Software bug fixes
Ethiopia	The ability to work offline (as there are other areas working offline) the Internet connection is slow.		Improve technical aspects	Technical/software improvement	Ability to work offline
Ethiopia	The data elements that are found on monthly service delivery report format and the registration book format do not match with each other and it's very difficult to generate report from the registration book	The data elements that are found on monthly service delivery report format and the registration book format do not match with each other	Improve technical aspects	Technical/software improvement	Software bug fixes
		and it's very difficult to generate report from the registration book	Improve technical aspects	Technical/software improvement	Improve software usability
Ethiopia	The disease codes that was given to us were wrong and when we try to enter data using the code we were unable to find the diseases on the dhis 2.		Improve technical aspects	Technical/software improvement	Software bug fixes

Ethiopia	The indicators in the dhis 2 should be revised for example low Birthweight rate should be included in neonatal data set		Improve technical aspects	Improve functionalities for data quality checks, and data analysis	Improve indicators calculation (low birthweight)
Ethiopia	The quality tool should be user friendly.		Improve technical aspects	Technical/software improvement	Improve software usability
Ethiopia	The speed of the server should be improved, data analysis should be user friendly and after data entry when generating report sometimes it don't match with the data entered.	The speed of the server should be improved	Improve technical aspects	Technical/software improvement	Improve software capacity
		data analysis should be user friendly	Improve technical aspects	Technical/software improvement	Improve software usability
		and after data entry when generating report sometimes it don't match with the data entered.	Improve technical aspects	Technical/software improvement	Software bug fixes
Ethiopia	There is an issue with doubling the counts when generating reports using DHIS2		Improve technical aspects	Technical/software improvement	Software bug fixes
Ethiopia	To differentiate between early and late Neonatal death.		Improve technical aspects	Improve functionalities for data quality checks, and data analysis	Improve indicators calculation (neonatal deaths)
Ethiopia	When generating report it should be capable of giving summarised report rather than only allowing as to see the report divided by thematic areas and dhis 2 should be capable of importing data from other sources.	When generating report it should be capable of giving summarised report rather than only allowing as to see the report divided by thematic areas	Improve technical aspects	Technical/software improvement	Software development
		and dhis 2 should be capable of importing data from other sources.	Improve technical aspects	Technical/software improvement	Integration of DHIS with other data systems
Ethiopia	after entering the data on dhis 2 when we try to generate report sometimes it shows wrong data or it doesn't display the result at all.		Improve technical aspects	Technical/software improvement	Software bug fixes
Tanzania	A PLACE TO REPORT NEONATAL DEATH E.G 0-7 DAYS		Improve technical aspects	Improve functionalities for data quality checks, and data analysis	Improve indicators calculation (neonatal deaths)
Tanzania	ALL INDICATORS SHOULD BE DEFINED CLEARLY IN SYSTEM AND HAVE UNIQUE FORM FOR CAPTURING DATA	ALL INDICATORS SHOULD BE DEFINED CLEARLY IN SYSTEM	Improve technical aspects	Technical/software improvement	Improve software usability
		AND HAVE UNIQUE FORM FOR CAPTURING DATA	Improve technical aspects	Technical/software improvement	Software development
Tanzania	BE ABLE TO WORK OFFLINE		Improve technical aspects	Technical/software improvement	Ability to work offline

Tanzania	BEABLE TO WORK OFFLINE		Improve technical aspects	Technical/software improvement	Ability to work offline
Tanzania	CREATING SPECIFIC FORM FOR CAPTURING NEWBORN INDICATORS		Improve technical aspects	Technical/software improvement	Software development
Tanzania	CREATION OF DATA SET FOR NEWBORN DATA		Improve technical aspects	Improve functionalities for data quality checks, and data analysis	Improve indicators calculation (newborn)
Tanzania	DEATH ARE BEING CAPTURED IN BOTH L&D AND EVENT CAPTURE HENCE THERE IS A NEED TO MERGE THE SYSTEM, TO DIGITILISE THE DATA COLLECTING TOOLS	DEATH ARE BEING CAPTURED IN BOTH L&D AND EVENT CAPTURE HENCE THERE IS A NEED TO MERGE THE SYSTEM	Improve technical aspects	Improve functionalities for data quality checks, and data analysis	Integration of DHIS with other data systems
		, TO DIGITILISE THE DATA COLLECTING TOOLS	Improve technical aspects	Technical/software improvement	Improve digitalization
Tanzania	IMPLEMENTATION OF OFFLINE BASED SYSTEM(IF ONLINE BASED SYSTEM AVAILABILITY OF INTERNET SHOULD BE FREQUENTLY)		Improve technical aspects	Technical/software improvement	Ability to work offline
Tanzania	IMPROVEMENT OF NEONATAL DATA ARRANGEMENT IN DHIS2, DATA SHOULD HAVE INDEPENDENT FORM FOR ALL INDICATORS		Improve technical aspects	Technical/software improvement	Software development
Tanzania	INACCURATE IN CALCULATION OF TOTAL THROUGH PIVOT TABLE		Improve technical aspects	Technical/software improvement	Software bug fixes
Tanzania	IT SHOULD BE MORE USER FRIENDLY BECAUSE YOU CANNOT SWITCH PAGES EASILY. IT TAKES A LONG TO LOAD	IT SHOULD BE MORE USER FRIENDLY BECAUSE YOU CANNOT SWITCH PAGES EASILY.	Improve technical aspects	Technical/software improvement	Improve software usability
		IT TAKES A LONG TO LOAD	Improve technical aspects	Technical/software improvement	Improve software capacity
Tanzania	NETWORK PROBLEMS TO BE SOLVED		Improve technical aspects	Technical/software improvement	Improve software capacity
Tanzania	NO A PLACE OF RECORDING CAUSE OF DEATH SPECIFIC FOR NEWBORNS ESPECIALLY WHEN A BABY IS STILLBIRTH		Improve technical aspects	Technical/software improvement	Software development
Tanzania	REPORTING NEONATAL DEATH EXAMPLE 0-7 DAYS		Improve technical aspects	Improve functionalities for data quality checks, and data analysis	Improve indicators calculation (neonatal deaths)
Tanzania	SEX DISAGGREGATED OF SOME NEONATAL INDICATORS LIKE STILLBIRTH		Improve technical aspects	Improve functionalities for data quality checks, and data analysis	Data disaggregation

Tanzania	SIMPLIFIED SYSTEM		Improve technical aspects	Technical/software improvement	Improve software usability
Tanzania	SPECIAL FORM FOR CAPTURING NEONATES DATA		Improve technical aspects	Technical/software improvement	Software development
Tanzania	SPECIFIC UNIQUE FORM FOR SPECIFIC INDICATOR OF NEONATES SHOULD BE INTRODUCED		Improve technical aspects	Technical/software improvement	Software development
Tanzania	THE SYSTEM TO BE ABLE TO CALCULATE PERCENTAGE COMPARING DATA FROM THE INTERACTIVE REPORT AND PIVOT TABLE ARE DIFFERENT.	THE SYSTEM TO BE ABLE TO CALCULATE PERCENTAGE	Improve technical aspects	Improve functionalities for data quality checks, and data analysis	Improve indicators calculation
Tanzania		COMPARING DATA FROM THE INTERACTIVE REPORT AND PIVOT TABLE ARE DIFFERENT.	Improve technical aspects	Technical/software improvement	Software bug fixes
Tanzania	THE SYSTEM TO BE FRIENDLY USED		Improve technical aspects	Technical/software improvement	Improve software usability
Tanzania	THE UPDATES DONE IN THE SYSTEM SHOULD BE SAME AS IN THE FORMS AND VICE VERSA		Improve technical aspects	Technical/software improvement	Improve software usability
Tanzania	THEY SHOULD ADD A PLACE TO RECORD SEPSIS		Improve technical aspects	Technical/software improvement	Software development
Tanzania	TO BE TRAINED ON DATA ENTRY AND DATA ANALYSIS		Improve technical aspects	Training improvement	Staff training
Tanzania	TO HAVE A SYSTEM THT DOES NOT DEPEND ON DATA TO ACCESS		Improve technical aspects	Technical/software improvement	Ability to work offline
Tanzania	TO HAVE A TOOL FOR RECORDING NEONATAL DATA INTO THE SYSTEM		Improve technical aspects	Technical/software improvement	Software devevlopment
Tanzania	TO HAVE MORE TRAININGS ON INDICATORS OF NEW BORN AND STILL BIRTH BECAUSE WE ARE THE ONES WHO DO DATA ENTRY IN THE SYSTEM		Capacity strengthening	Training improvement	Staff training
Tanzania	TO HAVE ONE REPORTING TOOL THAT IS SIMILAR TO THE SOURCE DOCUMENT		Improve technical aspects	Technical/software improvement	Software devevlopment
Tanzania	TO IMPROVE THE DATA COLLECTION TOOLS BECAUSE IF YOU IMPROVE DATA COLLECTION TOOLS YOU WILL IMPROVE THE SYSTEM		Improve technical aspects	Technical/software improvement	Software bug fixes
Tanzania	TO LEAVE BLANK THE CAUSES OF NEONATAL MORTALITY TO ALLOW THE USER TO TYPE IN THE CAUSE		Improve technical aspects	Technical/software improvement	Software bug fixes
Tanzania		TRAINING OF STAFF	Capacity strengthening	Training improvement	Staff training

	TRAINING OF STAFF AND TRANSFORMATION OF PAPER BASED TO ELECTRONIC SYSTEMS TO DECREASE DESCREPANCY	AND TRANSFORMATION OF PAPER BASED TO ELECTRONIC SYSTEMS TO DECREASE DESCREPANCY	Improve technical aspects	Technical/software improvement	Improve digitalization
Tanzania	WHEN THEY UPDATE THE SYSTEM SHOULD ALSO UPDATE IN THE DATA COLLECTION TOOLS		Improve technical aspects	Technical/software improvement	Improve software usability
Uganda	Additional of catchment population estimates per facility in the system		Improve technical aspects	Technical/software improvement	Software development
Uganda	DHIS2 system is specific on the date when the death occured for macerated still birth yet the mid wife may not be able to know, so that should be improved.		Improve technical aspects	Technical/software improvement	Software bug fixes
Uganda	Every staff having the basis knowledge on how to capture data and interpret it .the tools should be made user friendly .	Every staff having the basis knowledge on how to capture data and interpret it.	Capacity strengthening	Training improvement	Staff training
Uganda		the tools should be made user friendly .	Improve technical aspects	Technical/software improvement	Improve software usability
Uganda	I would like the system to send feedback directly to all the people using the HMIS reporting tools e.g the midwives.		Improve technical aspects	Technical/software improvement	Send direct feedback
Uganda	I would like the system to send feedback directly to the midwives.		Improve technical aspects	Technical/software improvement	Send direct feedback
Uganda	If one wants to generate a specific data of a certain village it doesn't allow,if it could allow Data from communities so that we improve on performance.		Improve technical aspects	Technical/software improvement	Send direct feedback
Uganda	If we could have electronic medical record system,not only restricted to the ART clinic which will be accessible or cutting across all entry points.	If we could have electronic medical record system,not only restricted to the ART clinic which will be accessible or cutting across all entry points.	Improve technical aspects	Technical/software improvement	Software development
Uganda	There is need for capacity building for lower level staff in data capturing and analysis. Employ people whoa re competent with IT related issues.	There is need for capacity building for lower level staff in data capturing and analysis.	Capacity strengthening	Training improvement	Staff training
		Employ people whoa re competent with IT related issues.	Improve availability of resources	Increase human resources	
Uganda	More quality checks on new born data		Improve technical aspects	Improve functionalities for data quality checks, and data analysis	Data quality
Uganda	More training on DHIS2 specifically on data entry extration and analysis; training on WHO data quality check.		Capacity strengthening	Training improvement	Staff training
Uganda	NICU needs it's own designed DHIS2 for collecting data majorly inpatient.		Improve technical aspects	Technical/software improvement	Software development
Uganda	Please refer to DQ_111.1.2 tool 2a Amudat district		Improve technical aspects	Improve functionalities for data quality checks, and data analysis	Data disaggregation

Uganda	Refer to ESF_110.1.1 ARUA city		Improve technical aspects	Improve functionalities for data quality checks, and data analysis	Improve indicators calculation
Uganda	Refer to OBAT_112.1.2 nakapiripirit District.		Capacity strengthening	Training improvement	Staff training
Uganda	Routen support supervision and trainings on new born indicators		Capacity strengthening	Training improvement	Staff training
Uganda	Scaling up the pilot MPDRS to health center III facilities		Improve technical aspects	Technical/software improvement	Send direct feedback
Uganda	Skills of the user need to be improved because it's a robust system. We are restricted to the facility only		Capacity strengthening	Training improvement	Staff training
Uganda	Sometimes we get a challenge when network jumps.<br><br>We have a challenge on entry of perinatal reviews because it has alot of information to be entered into the system.		Improve availability of resources	Technical/software improvement	Improve software capacity
Uganda	Staff at the entry data points should be trained and more human resource is needed.	Staff at the entry data points should be trained	Capacity strengthening	Training improvement	Staff training
Uganda		and more human resource is needed.	Improve availability of resources	Increase human resources	
Uganda	The aspects of having sex disaggregation; if we could have a detailed application software to only capture maternital and newborn data right from the time the mother was in ANC, to delivery then outcomes after delivery.	The aspects of having sex disaggregation;	Improve technical aspects	Improve functionalities for data quality checks, and data analysis	Data disaggregation
		if we could have a detailed application software to only capture maternital and newborn data right from the time the mother was in ANC, to delivery then outcomes after delivery.	Improve technical aspects	Improve functionalities for data quality checks, and data analysis	Improve indicators calculation (maternity and newborn)
Uganda	The system has limitations in terms of generating indicators,it is good at capturing raw data but it is not yet fully developed when it comes to indicators. There is need to make it zero rated because at the moment you cannot access it without internet which disturbs most times because of interruptions in the connection.	The system has limitations in terms of generating indicators,it is good at capturing raw data but it is not yet fully developed when it comes to indicators.	Improve technical aspects	Improve functionalities for data quality checks, and data analysis	Improve indicators calculation
		There is need to make it zero rated because at the moment you cannot access it without internet which disturbs most times because of interruptions in the connection.	Improve technical aspects	Technical/software improvement	Ability to work offline
Uganda	There is need to introduce a software which captures only maternity data on all indicators. Reports from facilities should be reported electronically on a daily basis because for example if there is a stock out of drugs in the middle of the months,you have to wait until the end of months to report on that ,you spend two weeks when the ministry does not know that you ran out of essential drugs .	There is need to introduce a software which captures only maternity data on all indicators.	Improve technical aspects	Improve functionalities for data quality checks, and data analysis	Improve indicators calculation (maternity)
		Reports from facilities should be reported electronically on a daily basis because for example if there is a stock out of drugs in the middle of the months,you have to wait until the end of months to	Improve technical aspects	Technical/software improvement	Software development

		report on that ,you spend two weeks when the ministry does not know that you ran out of essential drugs .			
Uganda	Timely mentorship on system updates in case of any		Capacity strengthening	Training improvement	Staff training
Uganda		Trainings should be done	Capacity strengthening	Training improvement	Staff training
Uganda	Trainings should be done and supply of computers in lower facilities	and supply of computers in lower facilities	Improve availability of resources	Increase physical resources	Increase available computers
Uganda	We need more training on neonatal data in DHIS2<br><br>Generally Refresher trainings in DHIS2 since most of the data staff are not trained		Capacity strengthening	Training improvement	Staff training

ANC – antenatal care, EDHIS – electronic district health information system, DHIS - district health information system, eRHIS – electronic routine health information system, KMC – kangaroo mother care, MPDRS - maternal and perinatal death surveillance and response

Table S26. Significant examples of comments for each major team emerged in the thematic analysis

<table>
<tr><th>Major themes</th><th>Main themes</th><th>Sub-themes</th><th>N of Comments</th><th>%</th></tr>
<tr><td>Improve technical aspects</td><td>Technical/software improvement</td><td>• Software development
• Integration of DHIS with other data systems
• Send direct feedback
• Software bug fixes
• Improve software usability
• Ability to work offline
• Improve software capacity
• Improve digitalization</td><td>73</td><td>57.5</td></tr>
<tr><td colspan="5">Quotations:</td></tr>
<tr><td colspan="5">• “Simplified system”
• “DHIS2 doesn't calculate some of the neonatal indicators even though the data is available and no clear source document for reportable data elements like neonatal asphyxia and when the DHIS 2 software undergoes update we must be briefed.”
• “During data analysis and generating report DHIS 2 has some problems it displays wrong data that can underestimate our performance, some of the disease types are not included in the DHIS 2 disease list and the down arrow of the keyboard doesn't work forcing us to use mouse for data entry that has significant impact on the workload. The above problems should be fixed.”</td></tr>
<tr><td>Improve technical aspects</td><td>Improve functionalities for data quality checks, and data analysis</td><td>• Data disaggregation
• Data quality
• Improve graph generation
• Improve cartographs generation
• Data standardization
• Improve indicators calculation
• Improve functionality to calculate targets</td><td>36</td><td>28.3</td></tr>
<tr><td colspan="5">Quotations:</td></tr>
<tr><td colspan="5">• “Being able to calculate indicators. Doesn't give us a map of which specific area of the catchment has the most referrals. Challenge in target setting using the electronic system (we largely use Excel or PowerPoint)”
• “Data visualization should be user friendly and it should be capable of calculating top ten causes of neonatal Mortality.”
• “More quality checks on newborn data”</td></tr>
<tr><td>Capacity strengthening</td><td>Training improvement</td><td>• Staff training</td><td>14</td><td>11</td></tr>
<tr><td colspan="5">Quotation:</td></tr>
<tr><td colspan="5">• “Staff at the entry data points should be trained…”
• “Timely mentorship on system updates in case of any”
• “Trainings should be done…”</td></tr>
<tr><td>Improve availability of resources</td><td>Increase human resources</td><td></td><td>2</td><td>1.6</td></tr>
<tr><td colspan="5">Quotations:</td></tr>
<tr><td colspan="5">• “…and more human resource is needed.”
• “Employ people who are competent with IT related issues.”</td></tr>
<tr><td>Improve availability of resources</td><td>Increase physical resources</td><td>• Increase available computers</td><td>1</td><td>0.8</td></tr>
<tr><td colspan="5">Quotation:</td></tr>
<tr><td colspan="5">• “…and supply of computers in lower facilities”</td></tr>
</table>

No suggestion given	1	0.8
Total	127	100

FIGURES

Figure S1. PRISM conceptual framework

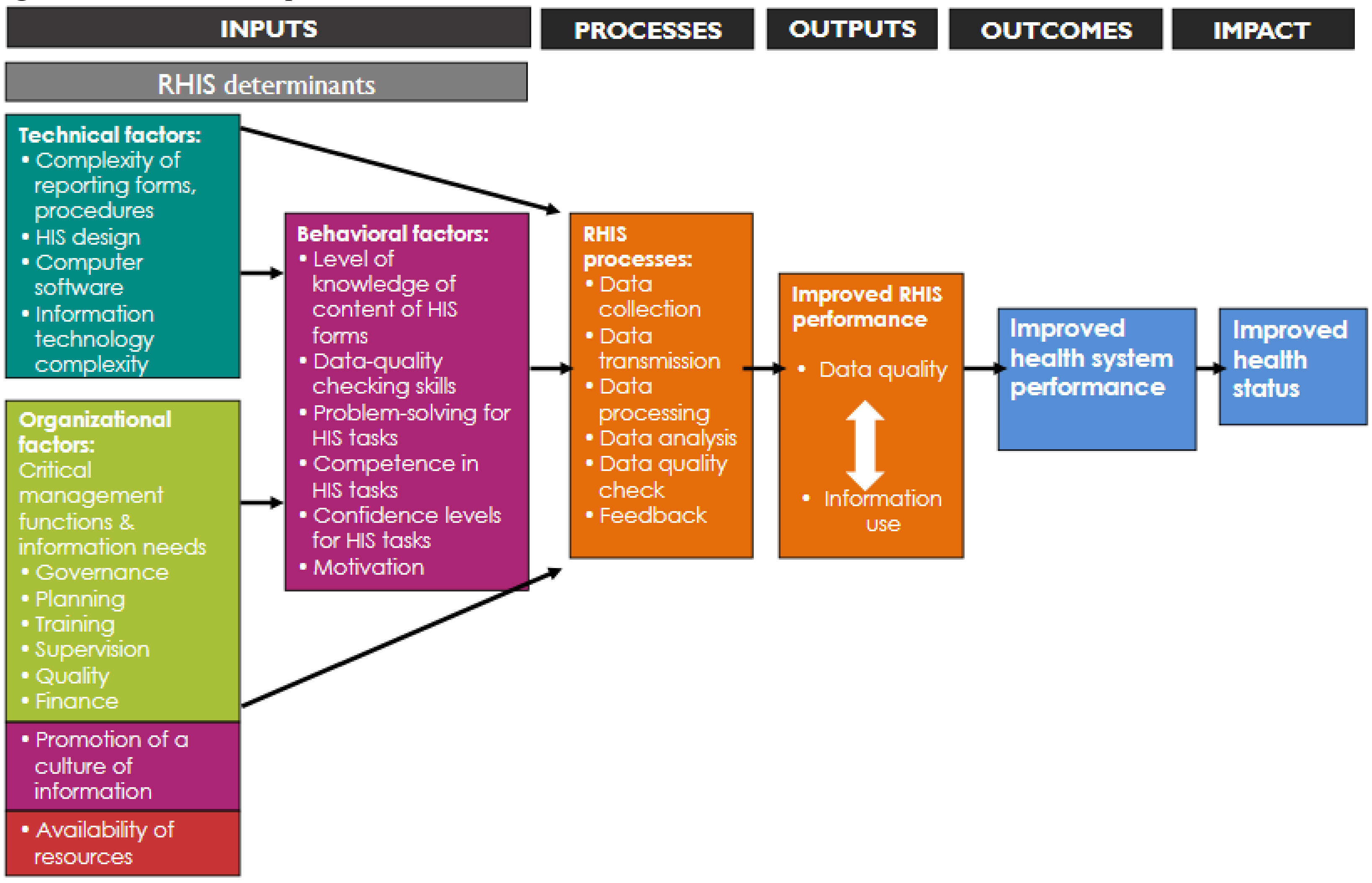

Aqil A, Lippeveld T, Hozumi D. PRISM framework: a paradigm shift for designing, strengthening and evaluating routine health information systems. Health Policy Plan. 2009;24:217–28. doi:10.1093/heapol/czp010

HIS – health information system, PRISM - performance of routine information system management, RHIS – routine health information system

Figure S2.1 Capabilities of the eRHIS users in Central African Republic, stratified by facility type (N = 21 sites)

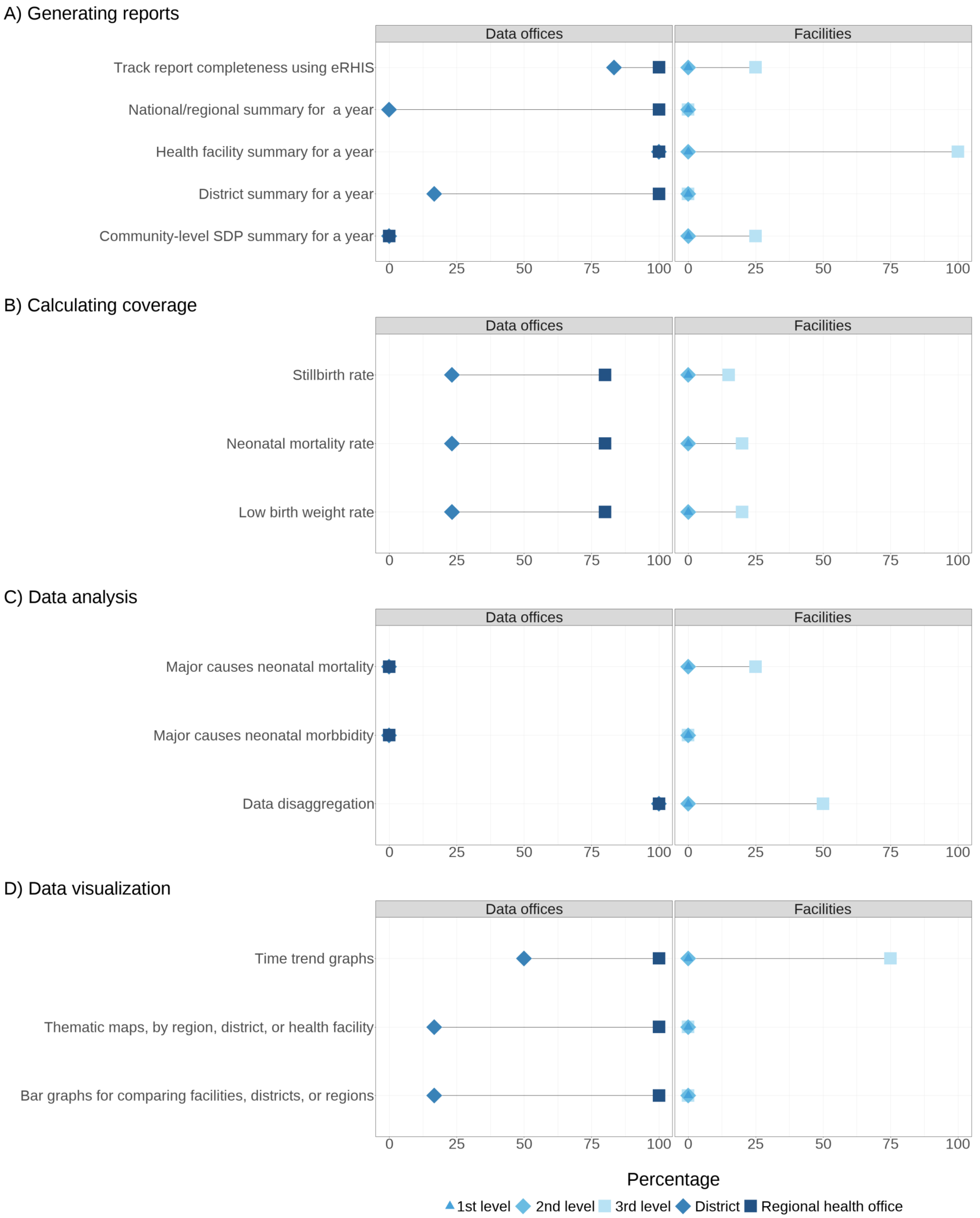

In the image each dot represents the value for one country. When two values overlap, one dot may not be visible.
CAR - Central African Republic, SDP - service delivery point

Figure S2.2. Capabilities of the eRHIS users in Ethiopia, stratified by facility type (N = 35 sites)

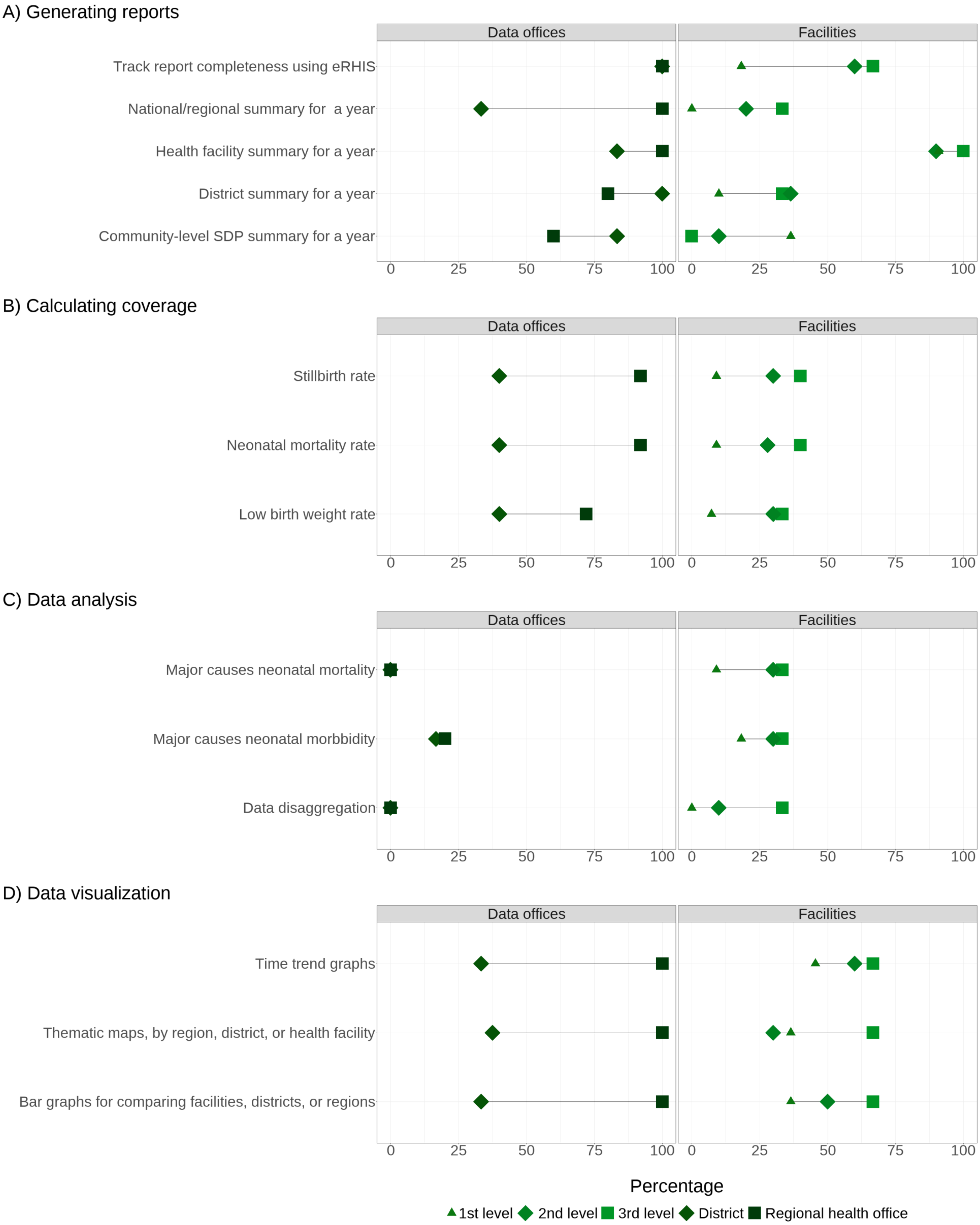

In the image each dot represents the value for one country. When two values overlap, one dot may not be visible.
CAR - Central African Republic, SDP - service delivery point

Figure S2.3. Capabilities of the eRHIS users in Tanzania, stratified by facility type (N = 46 sites)

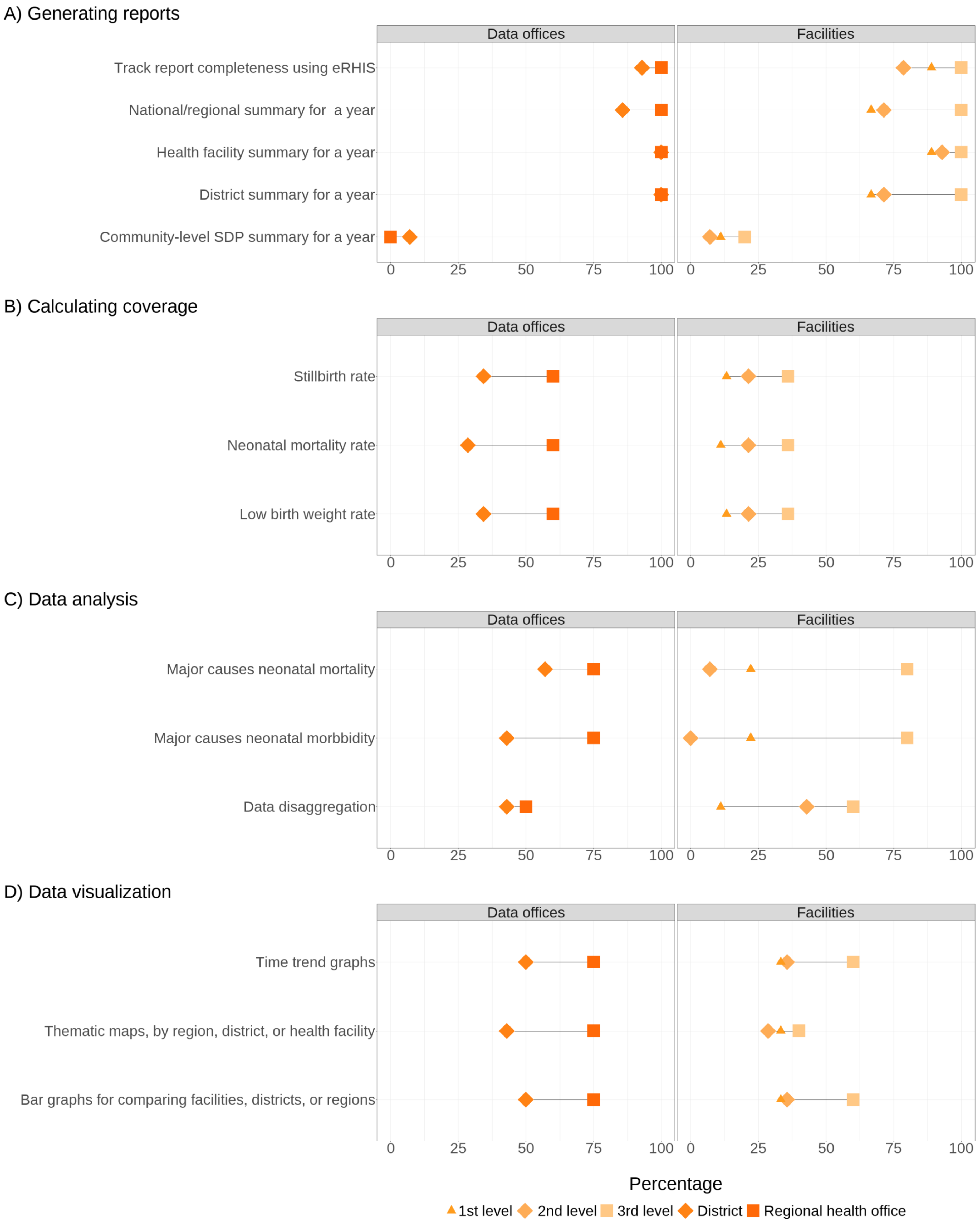

In the image each dot represents the value for one country. When two values overlap, one dot may not be visible.
CAR - Central African Republic, SDP - service delivery point

Figure S2.4. Capabilities of the eRHIS users in Uganda, stratified by facility type (N = 49 sites)

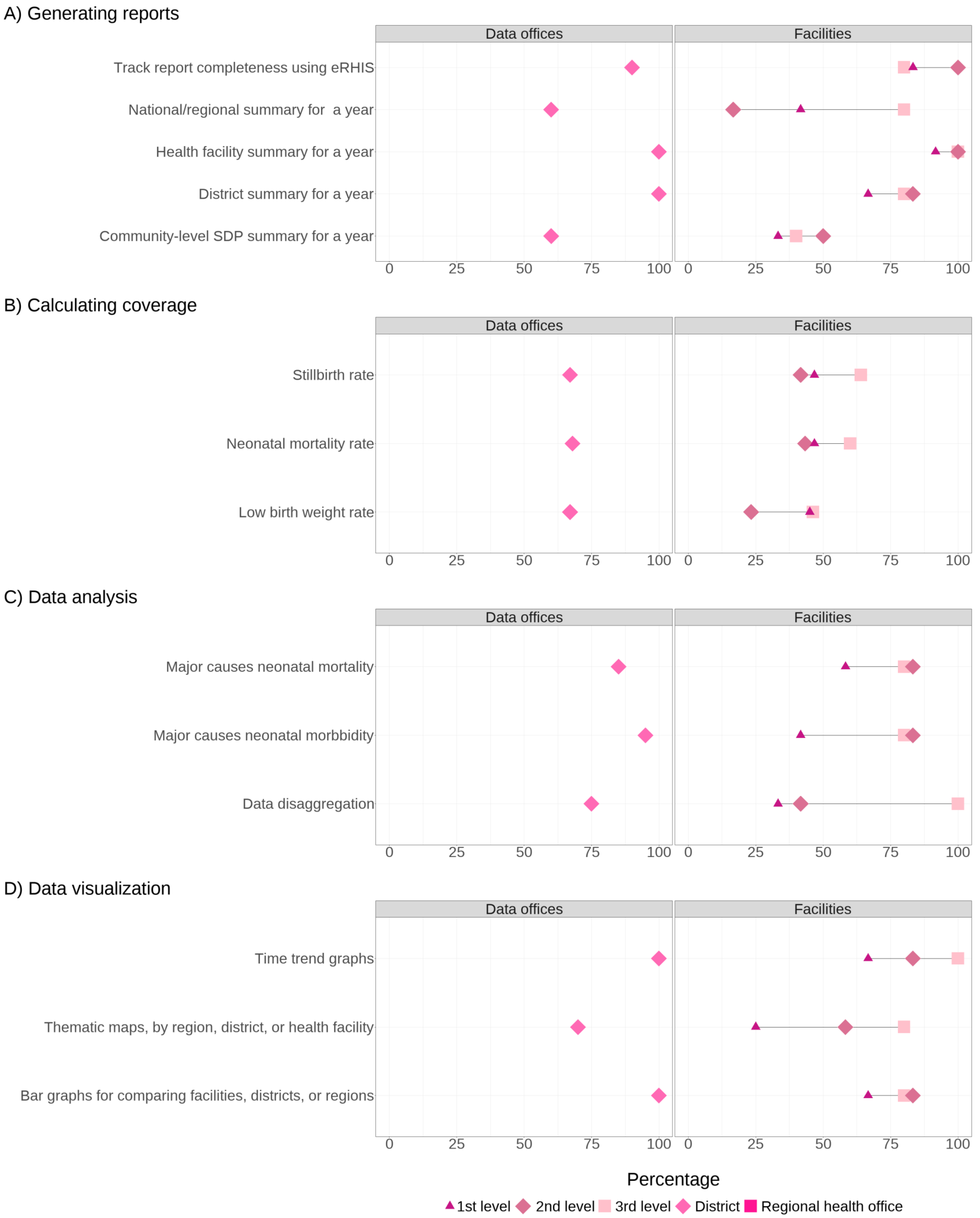

In the image each dot represents the value for one country. When two values overlap, one dot may not be visible.
CAR - Central African Republic, SDP - service delivery point

Figure S3.1. Practical skills in Central African Republic, stratified by facility type (N = 40 respondents)

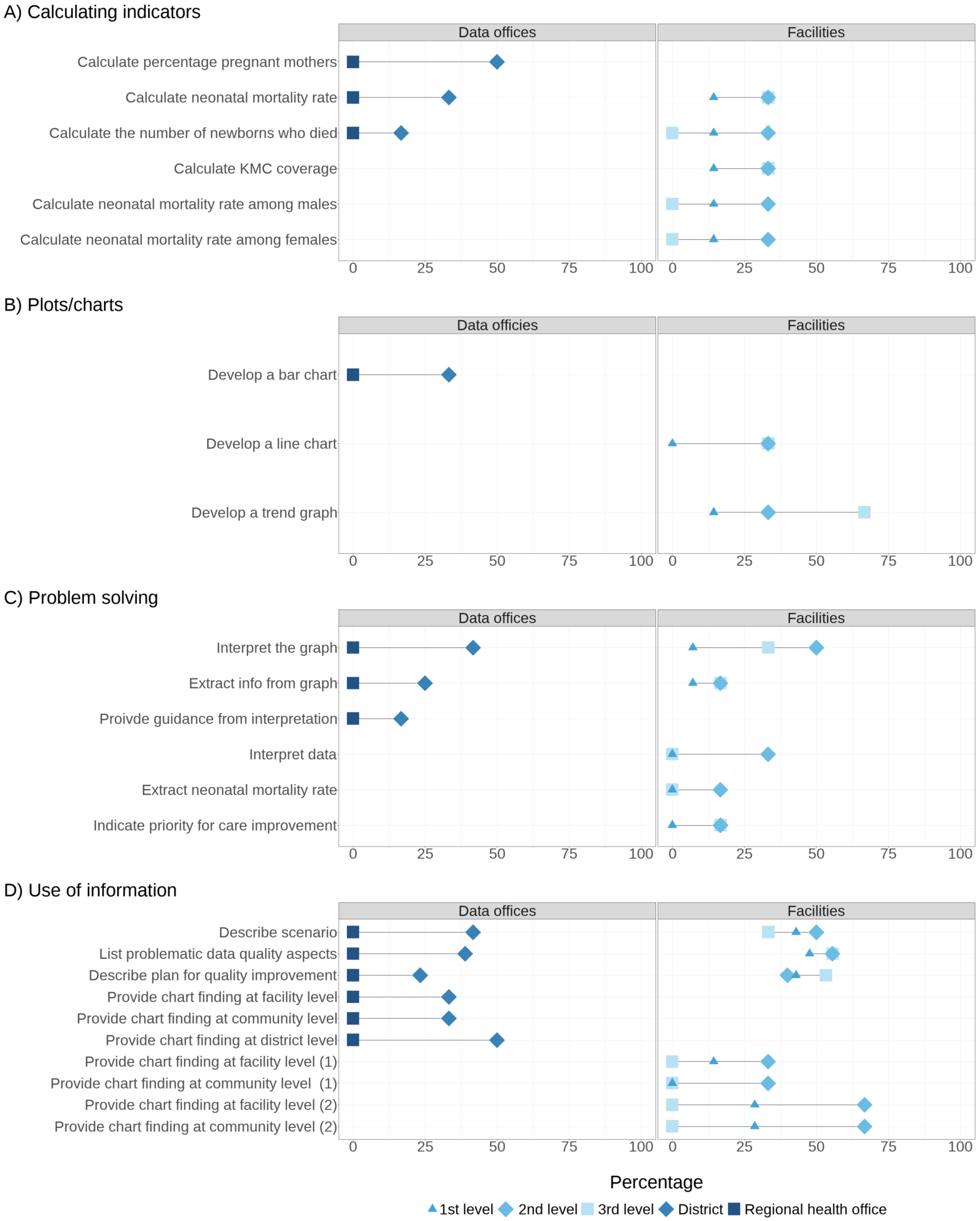

‘Provide chart finding at facility level’ and ‘Provide chart finding at community level’ are divided based on the pre-defined scenario the set of questions refer to. An empty space indicates that the specific indicator was collected only at a specific site level. In the image each dot represents the value for one country. When two values overlap, one dot may not be visible.
KMC - kangaroo mother care

Figure S3.2. Practical skills in Ethiopia, stratified by facility type (N = 99 respondents)

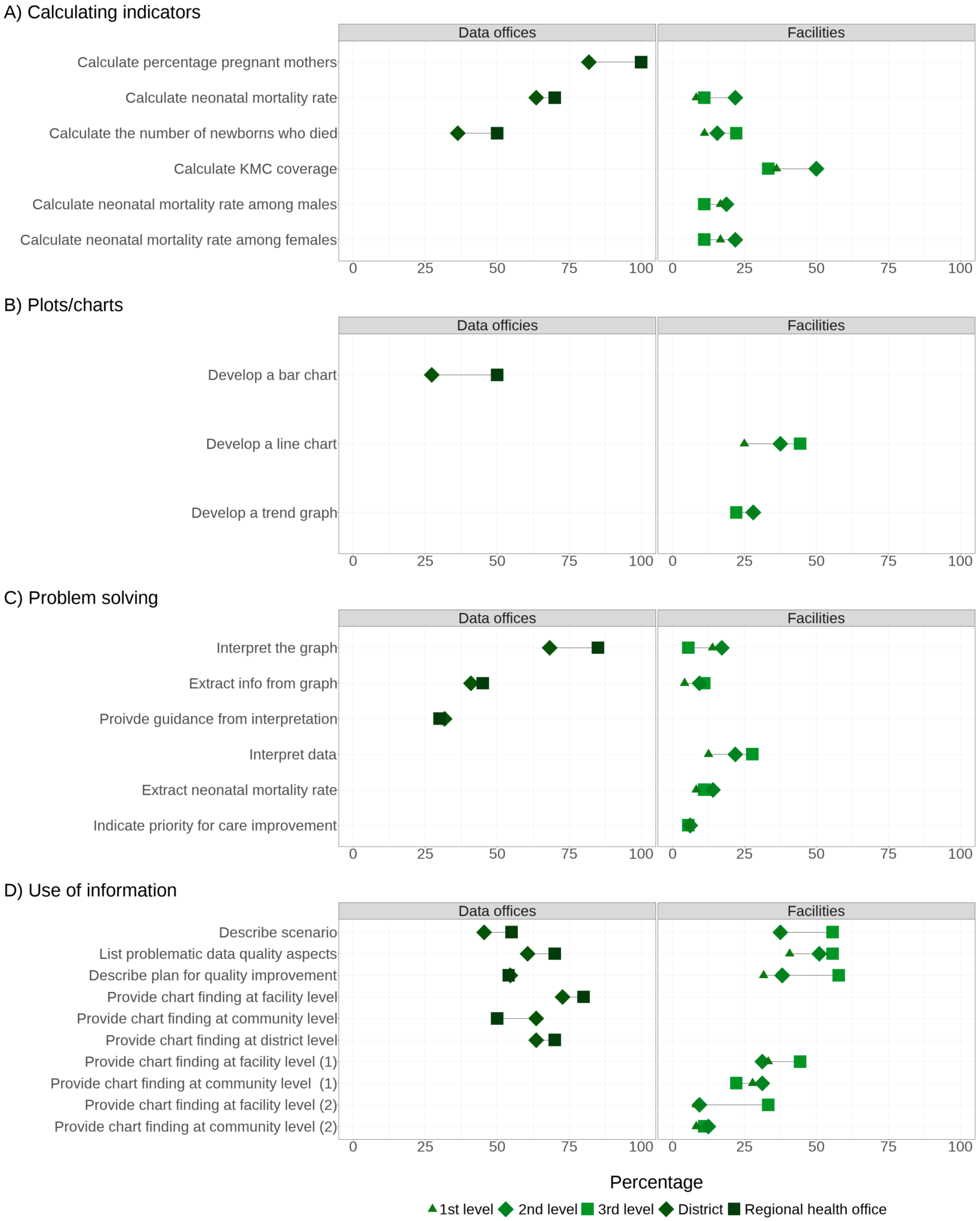

'Provide chart finding at facility level' and 'Provide chart finding at community level' are divided based on the pre-defined scenario the set of questions refer to. An empty space indicates that the specific indicator was collected only at a specific site level. In the image each dot represents the value for one country. When two values overlap, one dot may not be visible.
KMC - kangaroo mother care
.

Figure S3.3. Practical skills in Tanzania, stratified by facility type (N = 90 respondents)

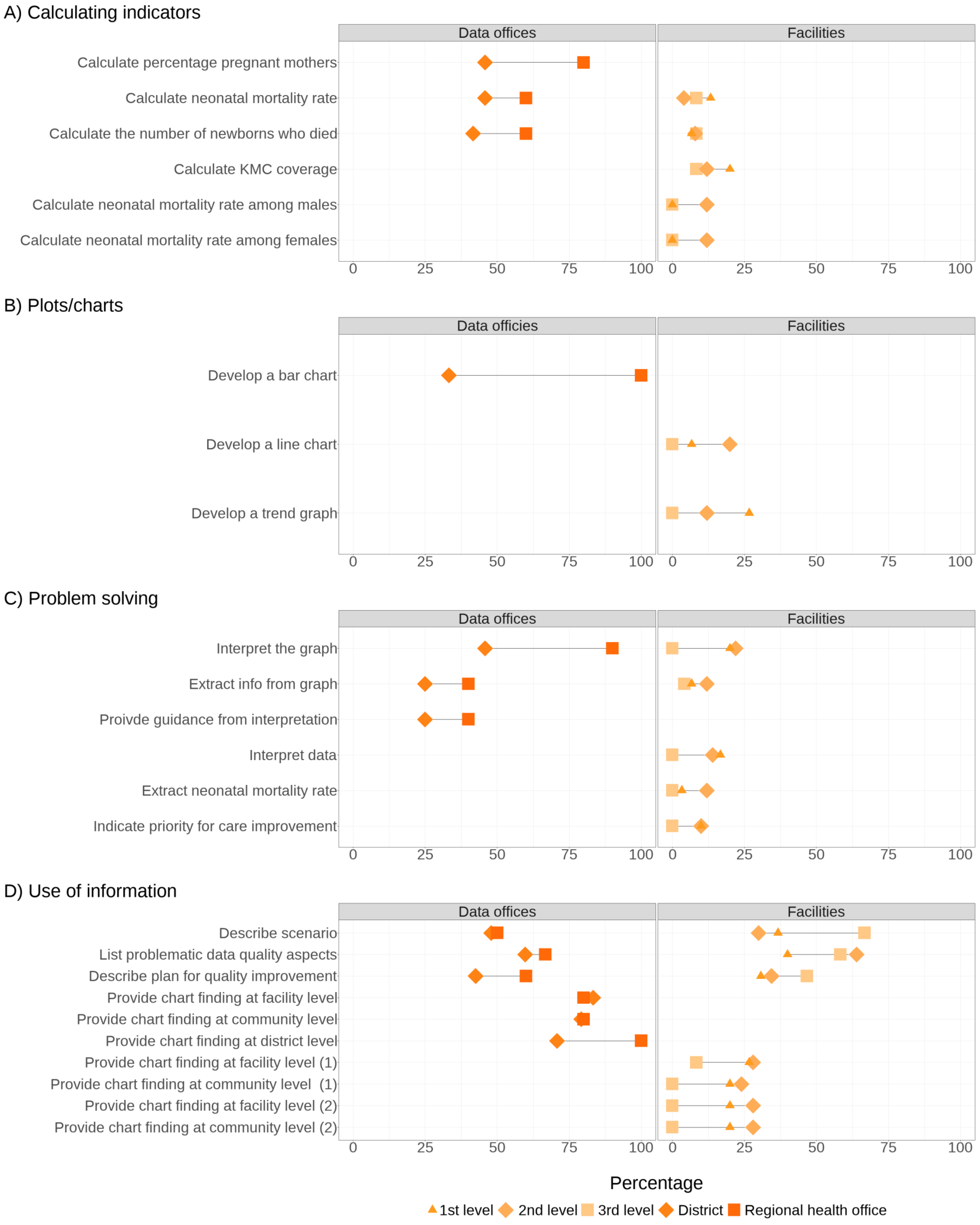

'Provide chart finding at facility level' and 'Provide chart finding at community level' are divided based on the pre-defined scenario the set of questions refer to. An empty space indicates that the specific indicator was collected only at a specific site level. In the image each dot represents the value for one country. When two values overlap, one dot may not be visible.
KMC - kangaroo mother care

Figure S3.4. Practical skills in Uganda, stratified by facility type (N = 120 respondents)

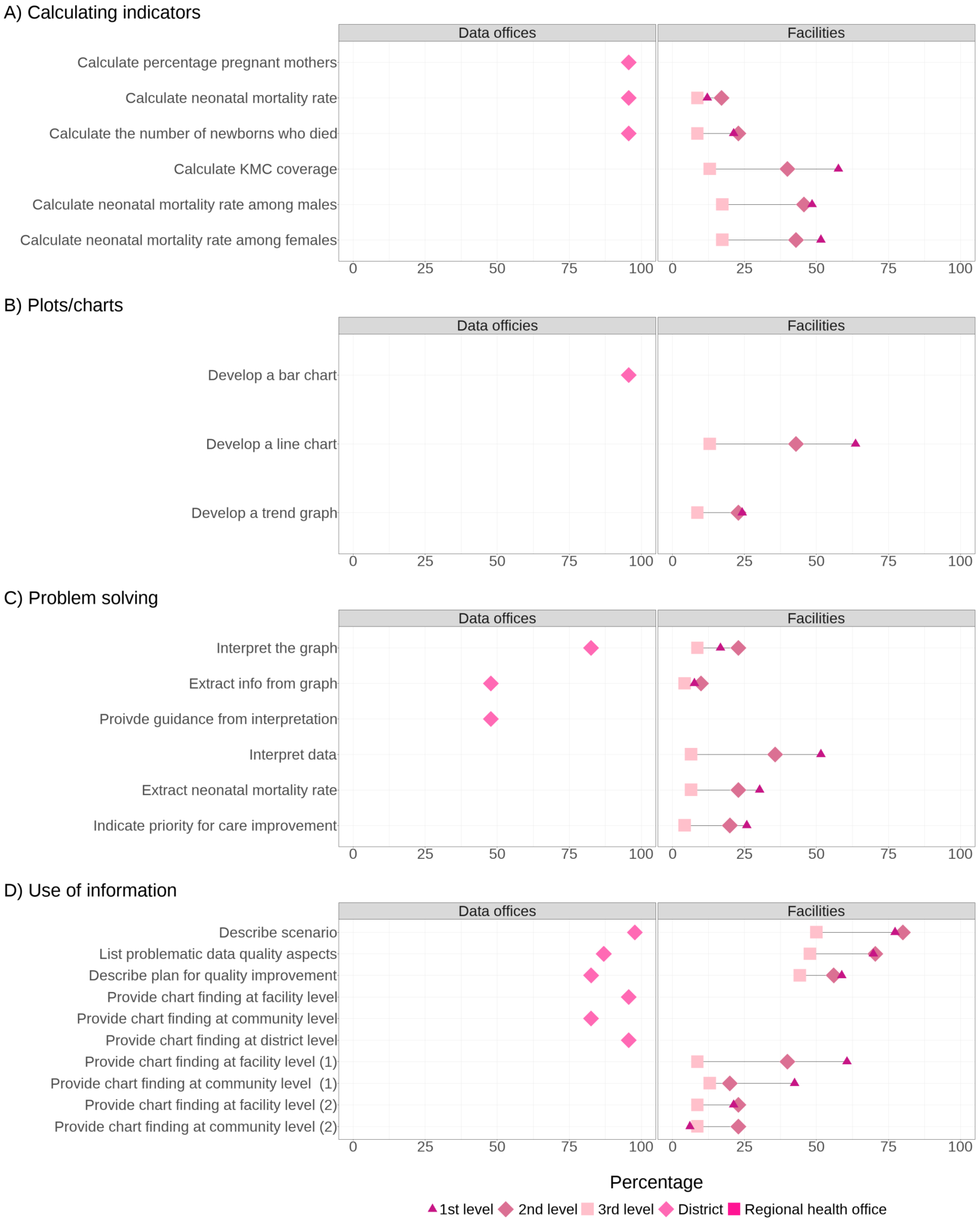

‘Provide chart finding at facility level’ and ‘Provide chart finding at community level’ are divided based on the pre-defined scenario the set of questions refer to. An empty space indicates that the specific indicator was collected only at a specific site level. In the image each dot represents the value for one country. When two values overlap, one dot may not be visible.
KMC - kangaroo mother care

Figure S4.1. Users' perspective regarding the eRHIS in Central African Republic, stratified by facility type (N = 21 respondents)

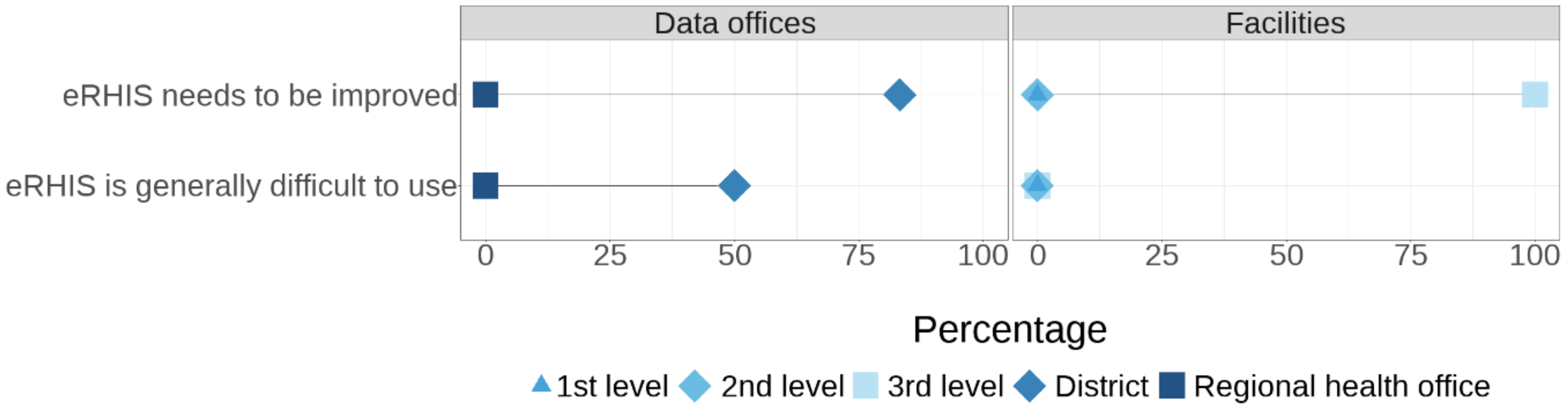

In the image each dot represents the value for one country. When two values overlap, one dot may not be visible
eRHIS = electronic Routine Health Information System

Figure S4.2. Users' perspective regarding the eRHIS in Ethiopia, stratified by facility type (N = 35 sites)

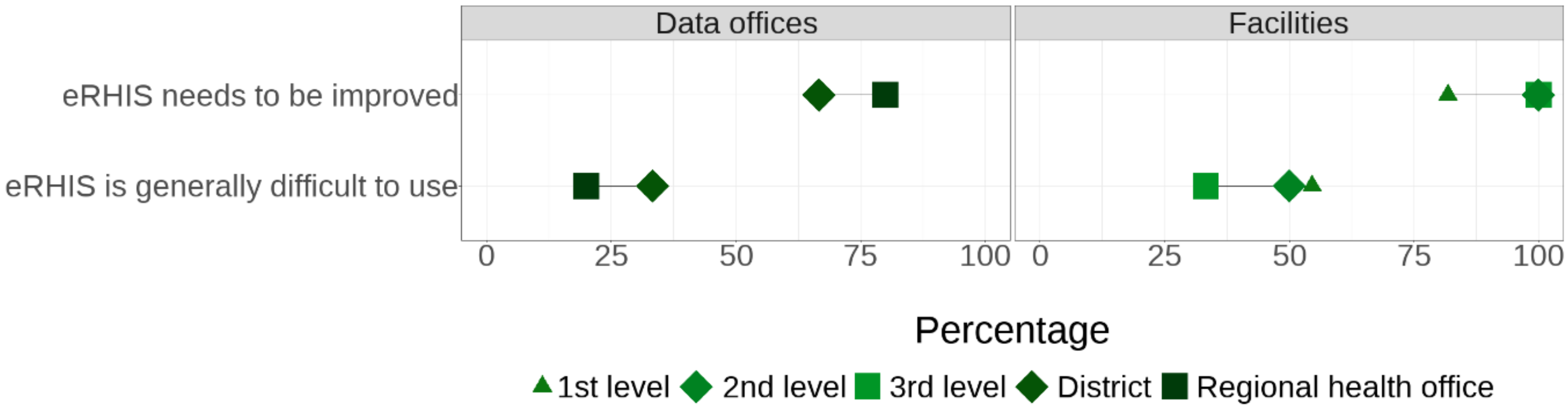

In the image each dot represents the value for one country. When two values overlap, one dot may not be visible
eRHIS = electronic Routine Health Information System

Figure S4.3. Users' perspective regarding the eRHIS in Tanzania, stratified by facility type (N = 46 sites)

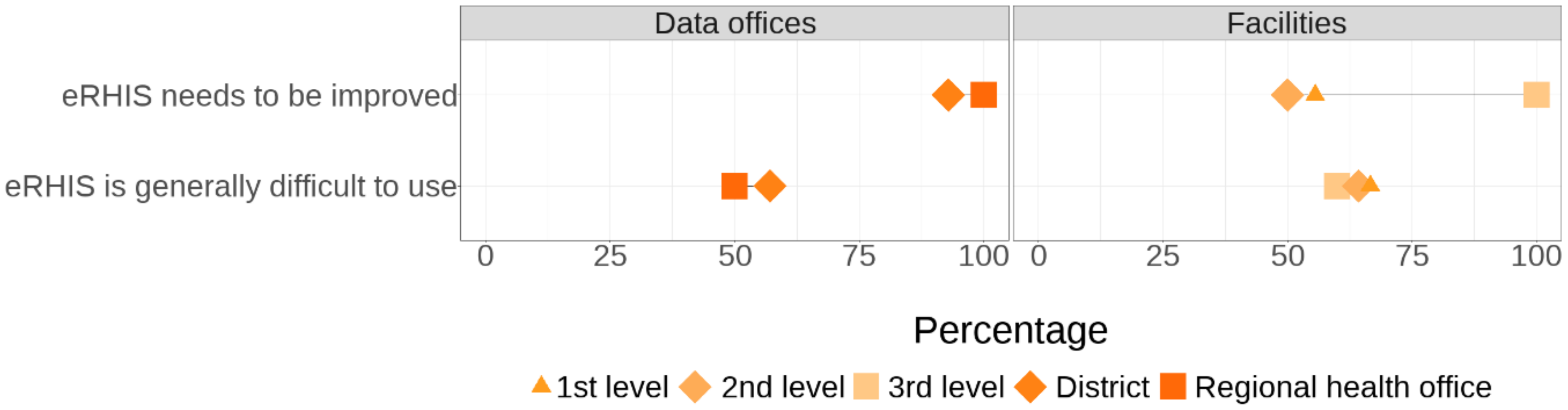

In the image each dot represents the value for one country. When two values overlap, one dot may not be visible
eRHIS = electronic Routine Health Information System

Figure S4.4. Users' perspective regarding the eRHIS in Uganda, stratified by facility type (N = 49 sites)

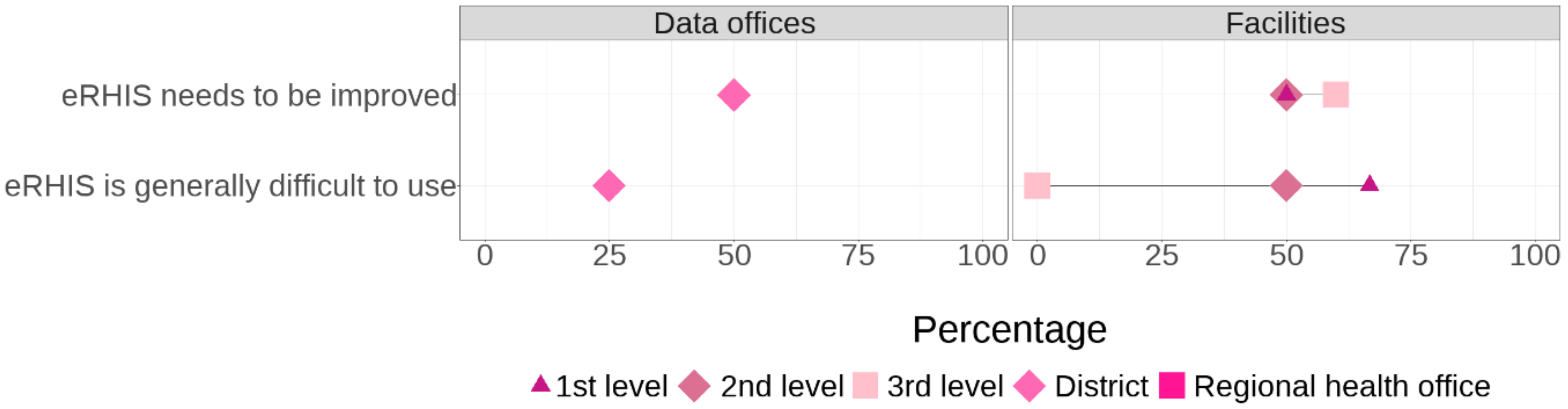

In the image each dot represents the value for one country. When two values overlap, one dot may not be visible
eRHIS = electronic Routine Health Information System
